# The influence of the polymerization approach on the catalytic performance of novel porous poly (ionic liquid)s for green synthesis of pharmaceutical spiro-4-thiazolidinones†

**DOI:** 10.1039/d0ra08647a

**Published:** 2020-12-15

**Authors:** Zahra Elyasi, Javad Safaei Ghomi, Gholam Reza Najafi, Mohammad Reza Zand Monfared

**Affiliations:** Department of Chemistry, Qom Branch, Islamic Azad University Post Box: 37491-13191 Qom I. R. Iran safaei@kashanu.ac.ir +98 31 55552935 +98 31 55912385; Department of Organic Chemistry, Faculty of Chemistry, University of Kashan Iran

## Abstract

Although poly (ionic liquids) (PILs) have attracted great research interest owing to their various applications, the performance of nanoporous PILs has been rarely developed in the catalysis field. To this end, a micro–mesoporous PIL with acid–base bifunctional active sites was designed and fabricated by two different polymerization protocols including hydrothermal and classical precipitation polymerization in this paper. Based on our observations, hydrothermal conditions (high temperature and pressure) enabled the proposed sonocatalyst to possess a great porous structure with a high specific surface area (*S*_BET_: 315 m^2^ g^−1^) and thermal stability (around 450 °C for 45% weight loss) through strengthening cross-linking. In a comparative study, the preferred nanoporous PIL was selected and utilized as the sonocatalyst in a multicomponent reaction of isatins, primary amines, and thioglycolic acid. In the following, a variety of new and known pharmaceutical spiro-4-thiazolidinone derivatives were synthesized at room temperature and obtained excellent yields (>90%) within short reaction times (4–12 min) owing to the substantial synergistic effect between ultrasound irradiation and magnetically separable catalyst.

## Abstract 2

Sustainable synthesize of a new mesoporous poly (ionic liquid) as acid–base bifunctional catalyst for environmental being preparation of monospiro derivatives has been developed.

## Graphical abstract

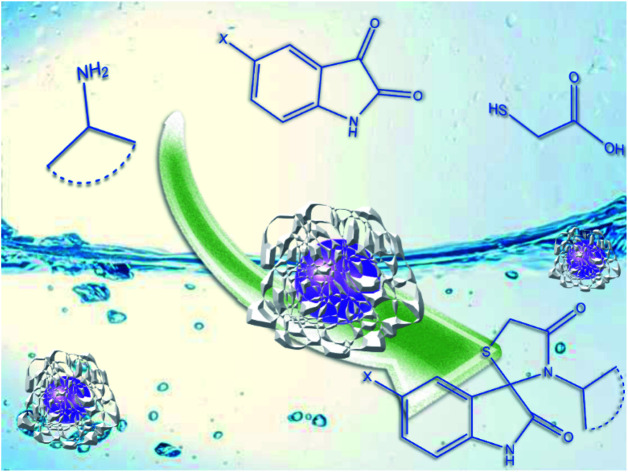

## 1. Introduction

**Fig. 1:**
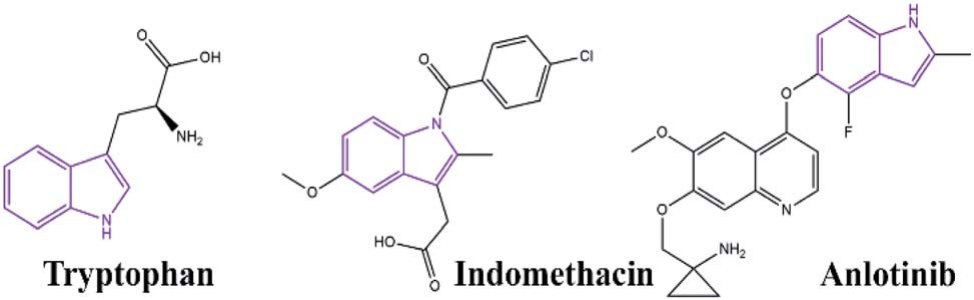
Indole scaffold in pharmacologically and biologically compounds.

The indole scaffold is known as one of the most important structures in heterocyclic chemistry owing to its widespread pharmacological and bioavailability applications, such as anti-inflammatory, antimicrobial, anti-hypertensive, anti-cancer, and anti-diabetic (Fig. 1).^1–6^ In addition, 4-thiazolidinone derivatives are privileged heterocyclic compounds with significant potential pharmaceutical performance, especially in the design of various anticancer agents.^7–9^ Therefore, several methods have been investigated for the synthesis of spiro[indole-thiazolidinones] compounds by reacting isatins with mercaptoacetic acid and amines under various conditions.^10–13^ However, many reported methods suffer from drawbacks, such as longer reaction times, high reaction temperature, use of flammable solvents, tedious workup, lower yields of the products, and expensive catalysts. Therefore, introducing an environmentally benign and straightforward synthetic strategy for the preparation of spiro[indolethiazolidinones] is an essential goal.

Radical-initiated polymerization processes, including dispersion, suspension, and emulsion polymerization, are traditional protocols for the construction of synthetic polymers owing to the polymer materials separation and ease of heat removal.^14–16^

In these heterogeneous polymerizations, polymeric surfactants or emulsifiers are used to stabilize the latex particles or monomer droplets. In recent decades, the precipitation polymerization has developed and in which any added surfactant or stabilizer is absent compared to traditional polymerization protocols.^17^ However, a low monomer concentration (<5 wt%)leads to heavy consumption of solvent and low relative yield (<50%). Recently, several new methods have been investigated to overcome these limitations, including solvothermal precipitation polymerization, redox-initiated precipitation polymerization, and photoinitiated precipitation polymerization.^18–22^ Ionic liquids bearing polymerizable groups could be polymerized under different conditions to form a new class of polyelectrolytes, which are named poly (ionic liquids) (PILs).^23,24^ They are able to provide the properties of both the ionic liquids and conventional polymers. Therefore, they opened up a wide window to various range of applications, such as catalysts, surface active materials, fuel cells, and membranes.^25–29^ Recently, the interest in their applications and synthesis kept growing, particularly those made from *N*-vinyl imidazolium-based monomers.^30^ Typically, imidazolium-based PILs are great metal-free catalysts due to their remarkable properties like good stability, tunable structure, and low vapor pressure. However, many PILs catalysts, reported in the literature, have low active site concentrations and poor porosities.^31,32^ Therefore, there is a challenge to synthesize porous PILs with a large amount of porosity and high specific surface areas. On the other hand, the stability of the porous materials is an important prerequisite for their various applications. The broad application rang of zeolites is attributed to their great hydrothermal and thermal stabilities.^33,34^ Although Metal Organic Frameworks (MOFs) have uniform pore sizes (due to their crystallinity) compared to zeolites, their applications are limited by their sensitivity to moisture, particularly in the presence of base or acid chemicals.^35,36^ While, porous ionic polymers (PIPs) containing chemically stable covalent bonds exhibited high hydrothermal, chemical, and thermal stabilities. Therefore, PIPs could be selected as efficient heterogeneous catalysts owing to their excellent stabilities which can facilitate the reuse and recovery of catalysts.^37,38^ Mesoporous PILs (as the main branch of the PIPs category) could be fabricated to possess intrinsic networks with exchangeable anions and porous structure among proper polymerization processes.^39,40^ Immobilization of PILs onto the magnetic supports is another practical procedure for improving the reusability and stability.^41^ In continuation of our investigations about introducing synthetic methodologies in the presence of efficient nanocatalyst,^42–46^ a new type of porous poly ionic liquid has been fabricated under two comparative polymerization methods. We designed and synthesized a new hypercrosslinked PIL immobilized on vinyl functionalized Co_3_O_4_ nanoparticles as a high effective catalyst for green preparation of spiro[indole-thiazolidinone] derivatives.

## 2. Results and discussion

Proving appropriate reaction conditions for NPs' surfaces is known as one of their most important modification. Having high magnetic behavior can also increase their importance due to the fact that they are generally absorbed to the magnet along with the magnetically stirring. In order to overcome this challenge, Co_3_O_4_ NPs were selected as the magnetic core instead of Fe_3_O_4_ NPs. In addition, the surface of MNPs has been modified by MPS before the polymerization process which caused grafting of polymeric networks on MNPs by covalent bonding. On the other hand, two different methods were applied to study the effect of polymerization protocol on the morphology and efficiency of the prepared catalyst. Based on the observations, prepared porous PIL is highly sensitive to the polymerization process. The catalyst obtained from the hydrothermal precipitation polymerization is in the class of nanoscale materials with high porosity structure. The high specific surface area in combination with porous morphology, and thermal stability supply catalysis application. Finally, the preferred catalyst was used to facilitate the one-pot synthesis of spiro-2,3-disubstituted-4-thiazolidinones in an aqueous system under ultrasound condition.

### 2.1. Structural and textural analysis of Co_3_O_4_@crosslinked p[AVIM]Br nanocatalyst

**Fig. 2:**
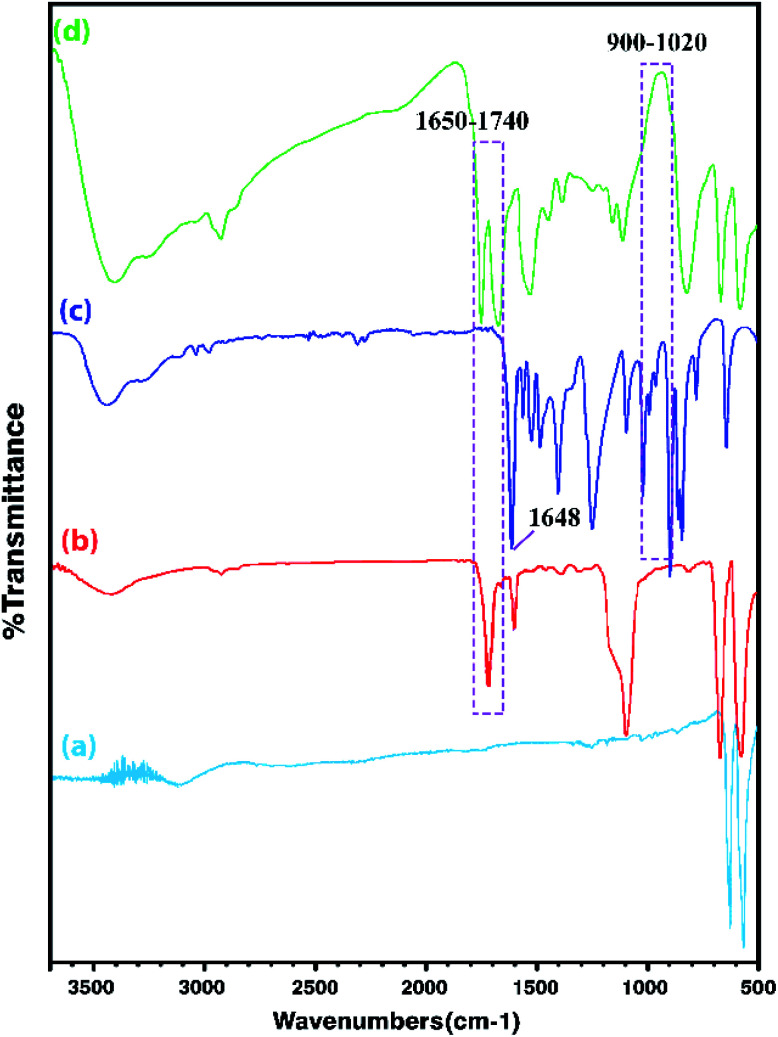
The FT-IR spectra of (a) Co_3_O_4_, (b) Co_3_O_4_@MPS, (c) ionic monomer, and (d) crosslinked Co_3_O_4_@p[AVIM]Br.

In order to obtain more accurate information, the FT-IR analysis of the Co_3_O_4_ NPs, Co_3_O_4_@MPS, [AVIM]Br monomers, and Co_3_O_4_@crosslinked p[AVIM]Br was performed. As shown in (Fig. 2a), the strong peaks at 655 and 575 cm^−1^ are attributed to the characteristic peaks of spinel Co_3_O_4_. The peaks at 655 cm^−1^ belong to the stretching vibration of metal–O in which M is Co^2+^. While the absorption band at 575 cm^−1^ can be attributed to the metal–O in which M is Co^3+^. Coating of MPS onto the Co_3_O_4_ NPs surface is also confirmed by peaks at 1100, 1540, and 1740 cm^−1^ which respectively belong to stretching vibrations of Si–O, C=C, and steric C=O (Fig. 2b). As it can be seen in (Fig. 2c), the stretching and bending vibration of the vinyl group of the imidazole ring have respectively appeared at 1648 cm^−1^ and 932–1020 cm^−1^. These mentioned peaks are not seen in the Co_3_O_4_@crosslinked p[AVIM]Br spectrum confirming polymerization (Fig. 2d).

Moreover, the characteristic peaks of N–C–N at 660 cm^−1^ and 1228 cm^−1^ are retained after the polymerization process in the spectrum as well. The imidazole H–C–C & H–C–N bending vibration also appears at 1168 cm^−1^ while the peak at 824 cm^−1^ corresponds to the in-plane imidazole ring bending.^47^ Notably, these mentioned peaks were observed in both IL monomer and PILs spectra. The doublet around 2850–3000 cm^−1^ corresponds to symmetric *ν*_s_(CH_2_) and asymmetric *ν*_as_(CH_2_) of the IL monomers. The broad peak at about 3300 cm^−1^ to 3530 cm^−1^ also belongs to the quaternary nitrogen of imidazolium with bromide.^47^ As shown in Fig. 2d, the observed strong peak at 1670 cm^−1^ is attributed to the characteristic peak of carbonyl groups of *N*,*N*′-methylenebisacrylamide.

**Fig. 3:**
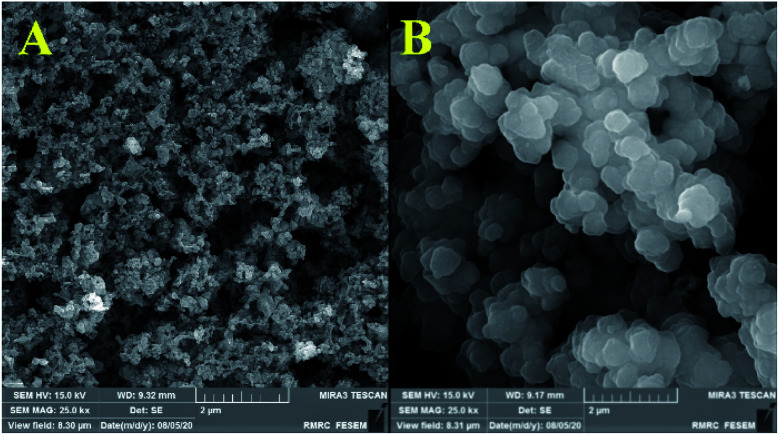
Comparative FESEM images of Co_3_O_4_@p[AVIM]Br, under (A) hydrothermal treated, and (B) classical precipitation polymerization in 2 μm.

**Fig. 4:**
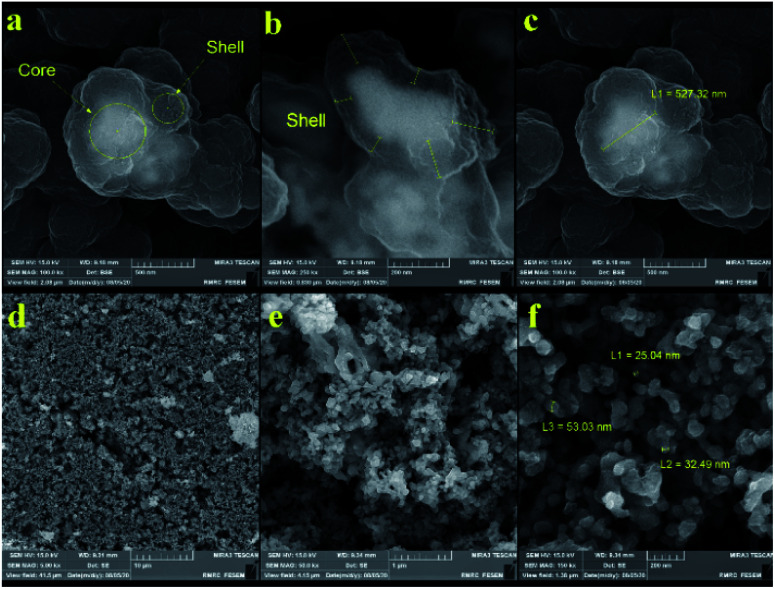
FE-SEM images of sonocatalyst obtained through (a–c) classical precipitation, and (d–f) hydrothermal precipitation polymerization.

Fig. 3 and 4 show the FE-SEM images for porous PILs synthesized by hydrothermal precipitation and classical precipitation polymerization process. As it can be seen, an obvious porous and crosslinked polymeric network was formed in both methods. As shown in Fig. 3, autoclaving temperature and pressure have a significant effect on the morphology in contrast. Based on the observations, hydrothermal conditions (high temperature and pressure) enabled the PIL to possess great porous structure through strengthening crosslinking.

Actually, the diameter of particles increased as the temperature decreased. Increasing the temperature from 80 °C (reflux) to 150 °C (autoclave) leads to the formation of a crosslinked magnetic core and polymeric shell structures with 25–50 nm (Fig. 4f). While classical precipitation polymerization provides the magnetic core diameter of ∼527 nm (Fig. 4c). Notably, the growth of particles is occurred due to their paramagnetic behavior and lack of enough energy force (temperature and pressure) for good dispersion. Moreover, the uniform porous structure of the Co_3_O_4_@crosslinked p[AVIM]Br was determined through Fig. 4d.

The porous structure is a key characteristics factor in the world of catalysts which affects their chemical and physical properties. As shown in FE-SEM images, the type of polymerization process extremely changed their morphology and structure which could have significant effects on the surface properties of the catalyst.

**Fig. 5:**
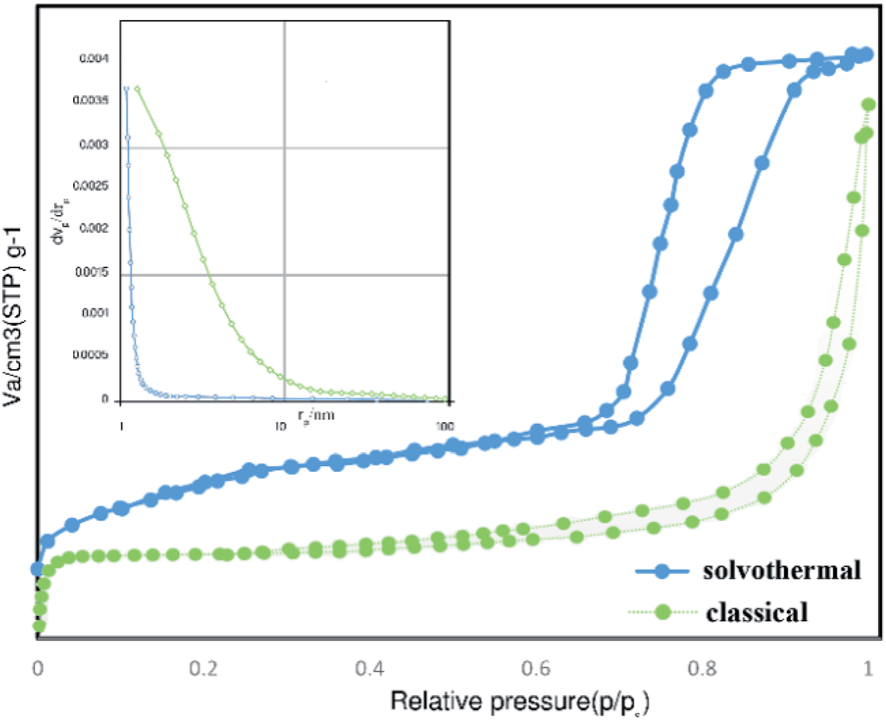
Pore size distributions and nitrogen adsorption desorption isotherms of Co_3_O_4_@p[AVIM]Br catalyst.

The N_2_ isotherm of the hydrothermal sample illustrated the type IV behavior with an obvious H2 type hysteresis loop in the pressure ranges from 0.65 to 1.00, implying the presence of microporous and mesoporous. Moreover, the classical sample of Co_3_O_4_@crosslinked p[VIM]Br showed the type IV isotherm with a distinctive H1 type hysteresis loop in the relative pressure ranges from 0.4 to 1.00, confirming the characteristic mesoporous structure. The specific surface area of prepared PIL through classical precipitation polymerization (∼110.0 m^2^ g^−1^) was less than one-third compared to the one that was obtained through hydrothermal precipitation polymerization (∼315.0 m^2^ g^−1^). Moreover, their pore size distributions and total pore volumes were quite different as demonstrated in Table 1. As it can be seen in Fig. 5, the absence of a step change in the pore size distribution of samples revealed that the distribution is non-zero <60 nm. Therefore, there are many pores in the network in which N_2_ gas is not condensed at the highest pressure. For the PIL obtained through classical precipitation polymerization, most pores were over 10 nm with a wide range distribution. Hydrothermal condition provided a higher degree of cross linking that led to more porous structure and smaller pore size distribution.

**Table 1:** Textural property of the Co_3_O_4_@crosslinked p[AVIM]Br

Entry	PIL	*S*_BET_ a (m^2^ g^−1^)	*V*_P_ b (cm^3^ g^−1^)	*D*_av_ c (nm)
1	Hydrothermal precipitation polymerization	315	0.29	3.1
2	Classical precipitation polymerization	110	0.37	10.7

a BET surface area.

b Total pore volume.

c Average pore size.

Thus, fabricated Co_3_O_4_@crosslinked p[AVIM]Br through hydrothermal polymerization resulted in a much higher proper pores structure and specific surface area which not only stem from the co-existence of random micro and mesoporous but also can greatly enhance their catalytic activity in organic synthesis.

**Fig. 6:**
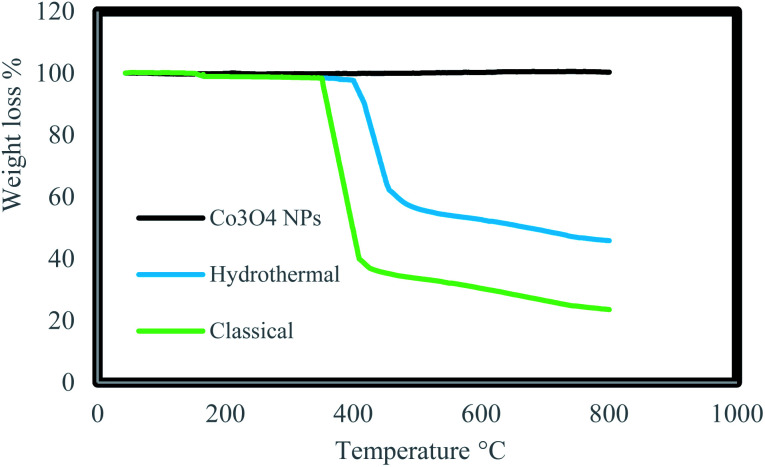
TGA analysis of Co_3_O_4_ NPs, prepared Co_3_O_4_@p[AVIM]Br under hydrothermal and classical precipitation methods.

Thermo Gravimetric Analysis (TGA) of the Co_3_O_4_@crosslinked p[AVIM]Br was separately investigated for Co_3_O_4_@crosslinked p[AVIM]Br catalyst under hydrothermal precipitation and classical precipitation polymerization methods to determine the effect of polymerization approach on thermal stability. The initial weight loss at low temperature (<200 °C) is attributed to the evaporation of physically adsorbed water owing to the hydrophilic nature of ionic liquid units (Fig. 6). The onset thermal degradation temperature (*T*_d_) value for Co_3_O_4_@crosslinked p[AVIM]Br was about 420 °C under hydrothermal condition. The hydrothermal treated Co_3_O_4_@p[AVIM]Br showed a drastic weight loss of 45% between the temperature 420–500 °C which can be due to the decomposition of the major bond between cross-linking and ionic monomers. Co_3_O_4_@p[AVIM]Br shows a fast weight loss up to 380 °C (70% weight loss) indicating the rapid decomposition of structure through classical method. It was assumed that a difference in the network forces (different degree of crosslinking) or morphology may explain the difference obtained in the thermal stability of samples. Thus, fabricated Co_3_O_4_@crosslinked p[AVIM]Br through hydrothermal polymerization illustrated more thermal stability.

**Fig. 7:**
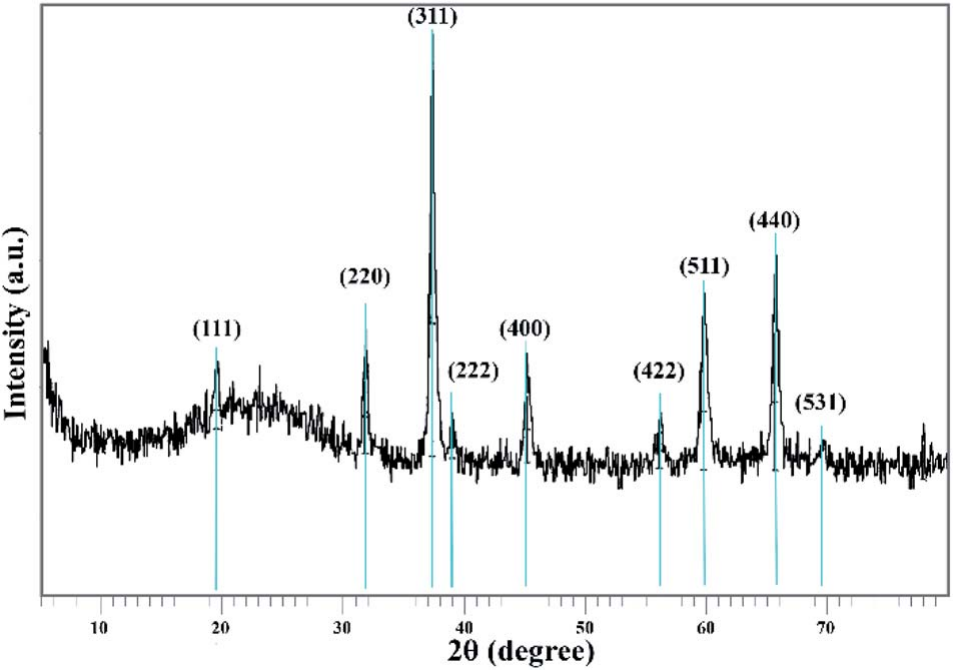
XRD pattern of sonocatalyst.

The Co_3_O_4_@crosslinked p[AVIM]Br structure was investigated by the measurements of powder X-Ray Diffraction (XRD) (Fig. 7). The XRD pattern of the proposed catalyst clearly illustrated 9 reflection peaks at 2*θ* = 19.45, 31.701, 37.263, 38.97, 45.174, 56.11, 59.773, 65.629, and 69.41. All of the appeared peaks were consistent with the peak positions of face-centered cubic Co_3_O_4_ (fcc, *Fd*3*m*, JCPDS card no. 01-080-1533). A broad peak around 20° belongs to the amorphous silica around the Co_3_O_4_ core. These experimental results showed that the modifications did not cause a phase change in the Co_3_O_4_ particles.

Energy-Dispersive X-ray spectroscopy (EDX) analysis and elemental mapping images of Co_3_O_4_@crosslinked p[AVIM]Br are presented in (Fig. S2†). In the EDX spectrum of the prepared PIL, expected elements (C, O, N, Co, Si, Br) have appeared in their regions. Elemental mapping results clearly determined that all the related elements, from PILs, are well distributed throughout the Co_3_O_4_@crosslinked p[AVIM]Br slices.

Vibrating sample magnetometer was investigated to confirm the magnetic behavior of the catalyst at room temperature. According to the linear magnetization diagram (Fig. S3†), the prepared catalyst has paramagnetic behavior. The magnetization was 0.48 emu g^−1^ at 14 000 Oe. It is worth mentioning that the final catalyst magnetization is enough and therefore it can be separated from the reaction mixture using an external magnet.

### 2.2. Measurement of catalytic activity of MNP@crosslinked p[AVIM]Br through the one-pot synthesis of spiro[indoline-3,2′-thiazolidinones]

In order to screen the catalytic activity of the prepared Co_3_O_4_@crosslinked p[AVIM]Br, a one-pot multicomponent reaction between isatin, aniline, and thioglycolic acid was first chosen as a model reaction. The reaction was optimized under various conditions and the experimental results are summarized in Table 2. Initially, the model reaction was carried out in H_2_O as the greenest solvent in the presence of different catalysis. Based on the data, the reaction, which was done without any catalyst, resulted in trace yield (Table 2, entry 1). According to the empirical results, although the Co_3_O_4_@crosslinked p[AVIM]Br obtained through classical precipitation polymerization was efficient (Table 2, entry 4), the hydrothermal Co_3_O_4_@crosslinked p[AVIM]Br was the best one (Table 2, entry 3). The significant effect of hydrothermal precipitation polymerization on the textural and structural properties (surface area, porosity, particle size, thermal stability) led to greater efficiency of Co_3_O_4_@crosslinked p[AVIM]Br. To adjust the amount of catalyst, different experiments were conducted. Accordingly, by increasing the amount of sonocatalyst from 5 to 10 mol%, the yield was increased from 80 to 94% while more nanocatalyst amount (15 mol%) did not improve the reaction yield (Table 2, entry 6). In order to determine the effect of ultrasonic agitation, the reaction was carried out in H_2_O as solvent by mechanical stirring under silent condition (Table 2, entry 7). The obtained result showed that the reaction yield is lower than sonication within a longer time. As could be guessed, there is a meaningful synergistic effect among catalyst (active catalytic sites) and US waves. Actually, collapsing the cavitation bubbles provided localized ‘‘hot spots” with high pressures that were responsible for the required bonding energy and molecular fragmentation. From the physical aspect, US irradiation facilitated the dispersion of the nanocatalyst in the media and therefore higher active surface area was generated. All of these phenomena can cause a reaction to take place rapidly. Next, the effect of solvents was variably investigated in the synthesis of (4a) as a model compound. Ethanol, acetonitrile, methanol, and THF afforded medium yields and more times (Table 2, entries 8, 10, 12, and 14). When the reaction was done in dioxane, toluene and DMF, the product yields were respectively about 55, 60, and 62%. Based on the results, H_2_O is an optimum solvent for the preparation of 4a. As a result, the use of 10 mol% of the hydrothermal-treated Co_3_O_4_@crosslinked p[AVIM]Br catalyst in H_2_O as solvent under US condition can be considered as the optimal condition (40 kHz, 100 W).

**Table 2:** Screening of different condition for the synthesis of desired product 4a^a^

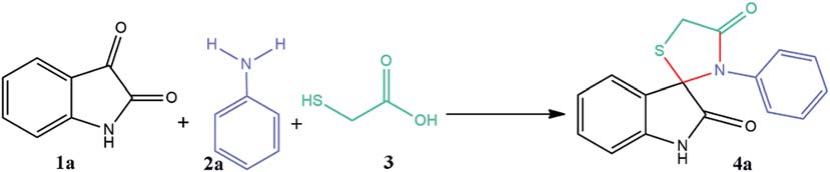					
Entry	Solvent	Catalyst	Condition	Time (min)	Yield%^b^
1	H_2_O	—	US	20	18
2	H_2_O	Co_3_O_4_ NPs (10 mol%)	US	8	48
**3**	**H**_**2**_ **O**	**(10 mol%)**	**US**	**6**	**94**
4	H_2_O	Classical-Co_3_O_4_@p[AVIM]Br	US	15	88
5	H_2_O	(5 mol%)	US	5	80
6	H_2_O	(15 mol%)	US	5	93
7	H_2_O	(10 mol%)	Δ	90	92
8	EtOH	(10 mol%)	US	10	90
9	EtOH/H_2_O	(10 mol%)	US	10	92
10	CH_3_CN	(10 mol%)	US	10	89
11	CH_2_Cl_2_	(10 mol%)	US	15	85
12	THF	(10 mol%)	US	30	80
13	Toluene	(10 mol%)	US	30	60
14	MeOH	(10 mol%)	US	10	91
15	DMF	(10 mol%)	US	20	62
16	Dioxane	(10 mol%)	US	20	55

a Reaction conditions: isatin (1 mmol), aniline (1 mmol) and thioglycolic acid (1 mmol) in the presence of hydrothermal treated MNP@crosslinked p[AVIM]Br.

b Isolated yield.

Subsequently, to demonstrate the scope and applicability of this catalyst in the one-pot three-component synthesis of spiro-2,3-disubstituted-4-thiazolidinone derivatives, the reaction was extended to other substituted isatins, and various primary amines (Tables 3 and 4). All the prepared compounds have been characterized by IR, ^1^HNMR, ^13^CNMR, and elemental analysis. The above results clearly showed that the present catalytic procedure is extendable to a great diversity of substrates to make a variety-oriented library of spiro-4-thiazolidinone. Changing the electronic structure of the substituents (C5 of isatins) was also acceptable with high yields. An aryl amine including electron-donating substituents (methoxy, methyl, and hydroxy) reacted well with thioglycolic acid and isatin derivatives generated the desired spiro products in excellent yields whereas the amines have less reactive electron withdrawing substituents, such as (chloro, and fluoro) that were found to be sluggish good yields. While the reactions of various isatins and thioglycolic acid with benzylamine were afforded the products in higher yields.

**Table 3:** Scope of catalytic activity of hydrothermal treated Co_3_O_4_@crosslinked p[AVIM]Br for synthesis of spiro-4-thiazolidinones^a^

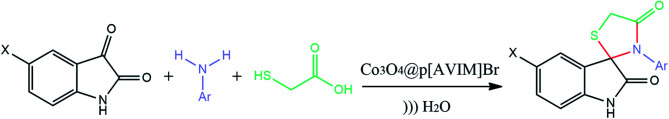					
Entry	X	Amine	Product	Time (min)	Yield%^b^
1	H	Aniline	4a	6	94
2	H	4-Chloroaniline	4b	8	92
3	H	4-Methoxyaniline	4c	6	96 NEW
4	H	Benzylamine	4d	7	96
5	H	4-Fluoroaniline	4e	10	92
6	H	2-Chloro-5-(trifluoromethyl)aniline	4f	6	98
7	H	4-Aminophenol	4g	7	96
8	Cl	Aniline	4h	4	97
9	Cl	4-Methylaniline	4i	4	95
10	Cl	Benzylamine	4j	7	96
11	Cl	4-Methoxyaniline	4k	4	98 NEW
12	Br	Aniline	4l	6	95
13	Br	Benzylamine	4m	6	95
14	Me	Aniline	4n	10	92
15	Me	4-Methylaniline	4o	9	93
16	Me	4-Fluoroaniline	4p	12	90

a Reactions conditions: isatin (1 mmol), amine (1 mmol) and thioglycolic acid (1 mmol), in the presence of hydrothermal treated Co_3_O_4_@crosslinked p[AVIM]Br (0.01 g).

b Isolated yield.

**Table 4:** Scope of catalytic activity of hydrothermal treated Co_3_O_4_@crosslinked p[AVIM]Br for synthesis of spiro-4-thiazolidinones^a^

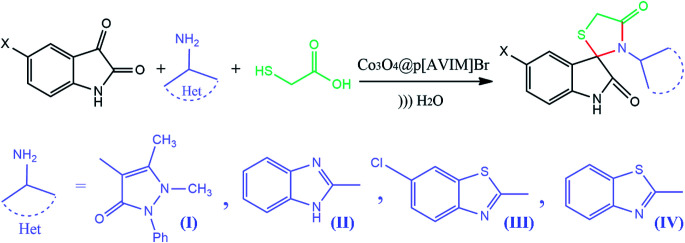					
Entry	X	Amine	Product	Time (min)	Yield%^b^
1	H	(I)	4q	5	92
2	Br	(I)	4r	5	96
3	Me	(I)	4s	8	93
4	H	(II)	4t	6	95
5	Cl	(II)	4u	98	7
6	Me	(II)	4v	10	91
7	H	(IV)	4w	6	95
8	Cl	(IV)	4x	5	98
9	Me	(IV)	4y	8	92
10	H	(III)	4z	7	96

a Reactions conditions: isatin (1 mmol), amine (1 mmol) and thioglycolic acid (1 mmol), in the presence of hydrothermal treated Co_3_O_4_@crosslinked p[AVIM]Br (0.01 g).

b Isolated yield.

In addition, a comparative study was performed between synthesized Co_3_O_4_@p[AVIM]Br and previously reported catalyst for the synthesis of spiro-2,3-disubstituted-4-thiazolidinone derivatives. As shown in Table 5, the highest yield and shortest reaction times belong to prepared porous PIL under mild and green conditions.

**Table 5:** Comparative study between reported catalyst and Co_3_O_4_@crosslinked p[AVIM]Br

Product	Catalyst^ref.^	Condition	Yield%	Time
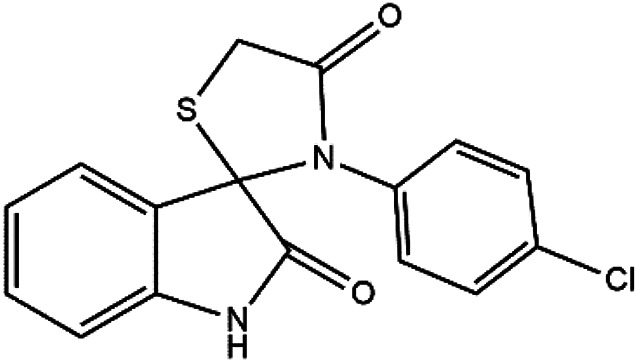	Acetic acid^48^	Benzene/reflux	75%	10 h
	Co_3_O_4_@p[AVIM]Br	US/water	92%	8 min
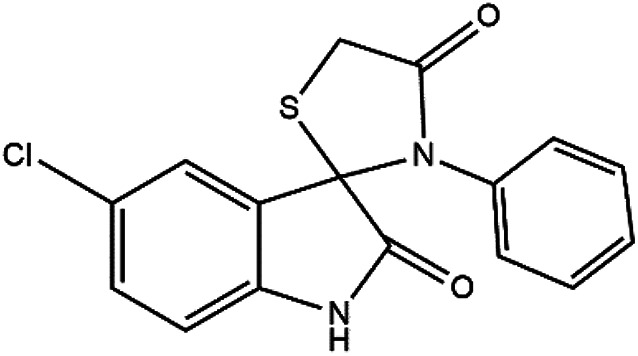	DBSA^10^	Room temperature/water	81%	30 h
	Co_3_O_4_@p[AVIM]Br	US/water	97%	4 min
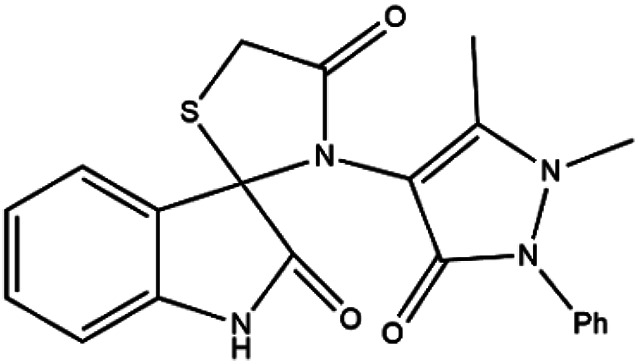	CTAB^49^	US/water	90	45 min
	Co_3_O_4_@p[AVIM]Br	US/water	92	5 min

**Scheme 1:**
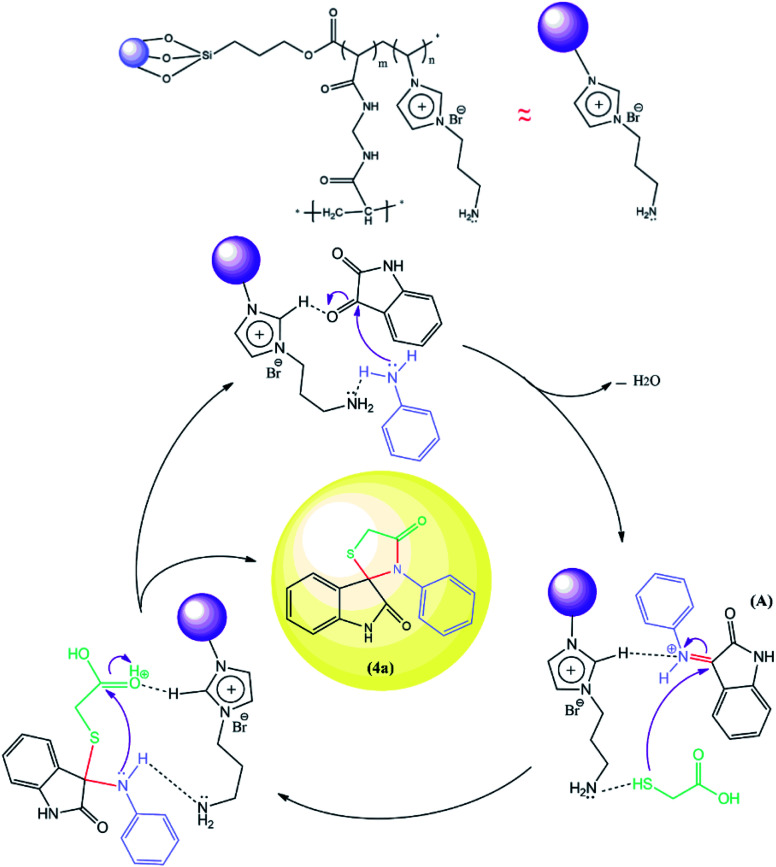
The catalytic cycle for the synthesis of spiro-4-thiazolidinones (4a).

The proposed reaction pathway mechanism for the preparation of spiro[indoline-3,2′-thiazolidinones] in the presence of prepared PIL is shown in Scheme 1. Herein, we introduce a new porous PIL including two different catalytic natures. Applied PIL including different acidic and basic active sites, can activate reaction *via* different routes by noncovalent interactions between two ionic groups of opposite charge (approaching isatin and aniline to initiate reaction). On the other hand, hydrogen bonding (a branch of noncovalent interactions) makes carbonyl group of isatin more susceptible to aniline nucleophilic addition. According to literatures,^50,51^ at the initial step of the catalytic pathway, the sonocatalyst facilitated the condensation of primary amine and isatin for the generation of the protonated Schiff base A through dual acid-basic sites. Then, imine (A) reacts with thioglycolic acid to generate the carbon–sulfur bond. The gem-diol compound generates through an intramolecular cyclization, and the desired product (4a) was obtained after the release of a water molecule. Furthermore, ultrasound irradiations have a significant role in multicomponent reactions. In general, asymmetric collapse form micro liquid jet with high speed enhanced the heat and mass transfer among the pores in the presence of ultrasound waves. This liquid jets activated the heterogeneous magnetic catalyst and improved the mass transfer by the disruption of the interfacial boundary layers.

**Fig. 8:**
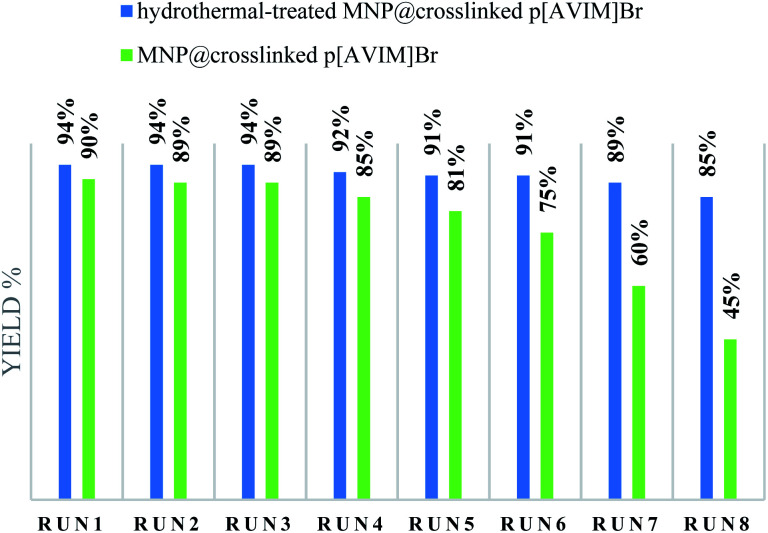
Recyclability of sonocatalyst.

**Fig. 9:**
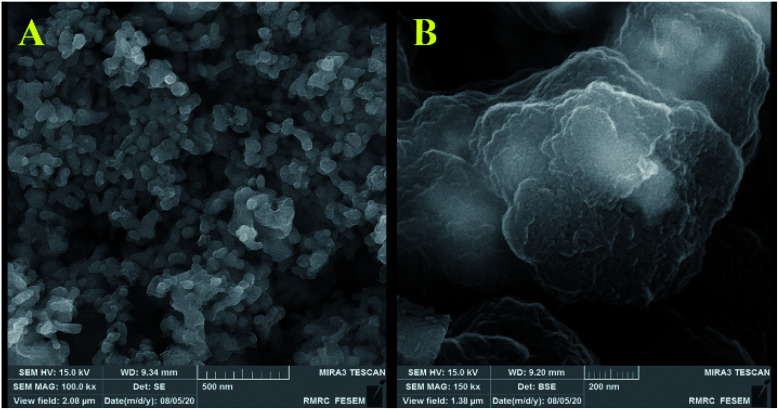
FE-SEM images of the recovered sonocatalyst after eight reaction run under the optimum reaction condition; (A) hydrothermal, and (B) classical precipitation polymerization.

Recoverability is known as one of the most important properties of the fabricated catalyst. At the end of the reaction, the nanocatalyst was magnetically separated and then the catalyst was washed three times with ethanol and H_2_O, dried 24 h, and reused directly with fresh substrates. It was shown that the hydrothermal treated Co_3_O_4_@crosslinked p[AVIM]Br could be recovered from the eight runs without any noticeable loss of its activity (Fig. 8). Next, to explore the probable change of sonocatalyst morphology, the FESEM images of the recovered Co_3_O_4_@crosslinked p[AVIM]Br after eight reaction runs were recorded for two different polymerization methods (Fig. 9). As shown, recycling did not significant change the morphology of hydrothermal treated sample (Fig. 9A), so the propose catalyst has high chemical stability.

## 3. Experimental

### 3.1. Chemicals and apparatus

1-Vinylimidazole (Alfa Aesar, 99%), 3-bromopropylamine hydrobromide (99%), ethyl acetate (ACS reagent, ≥99.5%), and 4,4′-azobis(4-cyanopentanoic acid) (≥98%) were obtained from Aldrich Chemical. All other chemical compounds were chosen from Merck Chemical. Fourier transform infrared spectra were recorded on an ABB Bomem MB-100 FTIR spectrometer. The XRD pattern was recorded on a Brucker AXS D8 diffractometer (Cu-Kα radiation). Thermo Gravimetric Analysis (TGA) was performed using a TGA Q 50 analyzer under the nitrogen atmosphere. The pore size distribution curves and nitrogen sorption isotherms were checked on a BELSORP-MINI analyzer while the samples were degassed at 473 K for 3 h. The specific surface area of samples was determined by Brunauer–Emmett–Teller (BET) calculation. Pore size distributions were estimated using Barrett–Joyner–Halenda (BJH) method. FE-SEM images were prepared with MIRA3TESCAN-XMU. Elemental analyses (EDS) were obtained on a PerkinElmer analyzer. Vibrating Sample Magnetometer (VSM) was made by MDK-I. R. Iran. NMR spectra were recorded on a Bruker 300 MHz spectrometer with TMS as an internal standard and DMSO-d^6^ as solvent.

### 3.2. Synthesis of vinyl functionalized Co_3_O_4_ NPs

Co_3_O_4_ NPs were prepared based on our previously reported procedure.^52^ Cobalt(ii) nitrate hexahydrate (8.60 g) was dispersed in ethanol (100 mL) at room temperature for 30 min. Then, oxalic acid (2.14 g) was swiftly added and the mixture was stirred for 90 min at 40 °C. The obtained pink precipitate was calcined at 400 °C for 2 h. A mixture of Co_3_O_4_ MNP (1 g) in 50 mL ethanol was prepared and the ammonia solution (2 mL) was then added to it. Next, 3-(trimethoxysilyl)propyl methacrylate (MPS) (10 mmol) was slowly added and the solution was stirred under reflux condition for 24 h. Finally, the MPS coated Co_3_O_4_ NPs (MNP@MPS) were separated by an external magnet and washed with ethanol (3 × 30 mL) to remove the unreacted compounds and then was dried overnight at 45 °C.

### 3.3. Synthesis of the ionic monomer

1-Aminopropyl-3-vinylimidazolium bromide ([AVIM]Br) was prepared under ultrasound condition for the first time. Briefly, 1-vinyl imidazole (3 mmol) and 10 mL ethanol were added to a two-necked flask equipped and then 3-bromopropylamine hydrobromide (3 mmol) was added into the flask and sonicated under nitrogen atmosphere. Finally, [AVIM]Br·HBr (5 mmol) was dissolved in the water (10 mL) and then equimolar sodium hydroxide was added and sonicated for 10 min at room temperature to form the isolated [AVIM]Br. The chemical structure of the product was determined using the FTIR, ^1^H NMR, and ^13^C NMR analysis ([AVIM]Br yield = 90%). White solid, yield: 90%; IR (CHCl_3_) *ν*_max_: 3440, 2930, 2845, 1648, 1228, 1168, 1020, 932, 824, 660 cm^−1^, ^1^H NMR (300 MHz, DMSO-*d*_6_) *δ* (ppm): 7.95 (s, 1H), 7.54 (s, 1H), 7.01 (s, 1H), 5.50–5.77 (d, 2H), 5.18 (s, 1H), 4.84–4.80 (t, 1H), 3.60–3.5 (t, 2H), 2.90–2.84 (t, 2H), 2.10–2.05 (m, 2H). ^13^C NMR (100 MHz, DMSO-d_6_) *δ* (ppm): 139.7, 124.5, 114.0, 107.5, 35.0, 33.3, 29.3.

### 3.4. Fabrication of porous poly ionic liquid through the precipitation polymerizations

Co_3_O_4_@MPS (0.5 g), 1-aminoethyl-3-vinylimidazolium bromide (0.45 g, 0.005 mol) and *N*,*N*′-methylenebis acrylamide (0.46 g, 0.003 mol) as crosslinker were dissolved into 10 mL dry methanol. The preparation procedure was followed by the addition of ACPA (1% mol, 0.029 g) which was refluxed for 3 h. Next, the obtained gray powder was washed with water (2 × 20) and dried at 40 °C for about 18 h.

### 3.5. Fabrication of porous poly ionic liquid through the hydrothermal precipitation polymerization

**Scheme 2:**
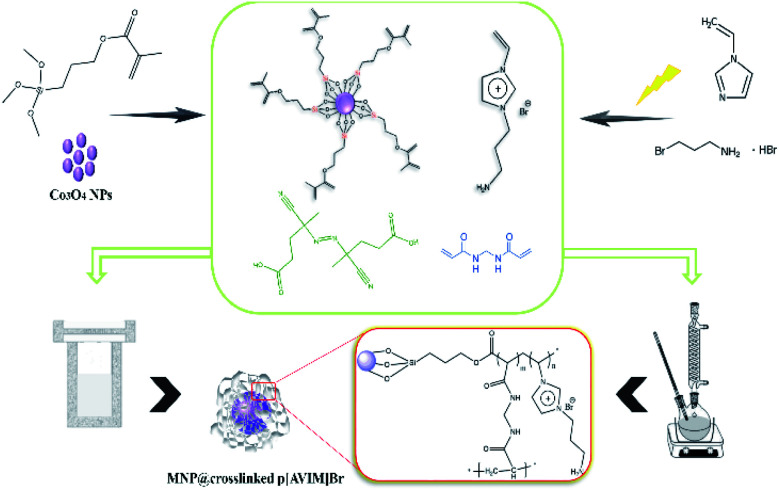
Schematic representation of the fabrication of Co_3_O_4_@crosslinked p[AVIM]Br under two different polymerization approach.

As a typical procedure, Co_3_O_4_@MPS (0.5 g), 1-aminoethyl-3-vinylimidazolium bromide (0.45 g, 0.005 mol), *N*,*N*′-methylenebis acrylamide (0.46 g, 0.003 mol), and ACPA (1% mol, 0.029 g) were mixed in 10 mL deionized water. After vigorous stirring at ambient temperature for 2 h, the mixture was transferred into steel autoclave at 100 °C for 24 h. Finally, the dark monolithic solid was collected and kept in a vacuum drying oven at 40 °C for 12 h. The specific fabrication rout of porous PIL is shown in Scheme 2.

### 3.6. General preparation of spiro-4-thiazolidinones

A mixture of isatin (1 mmol), thioglycolic acid (1 mmol), various primary amines (1 mmol), and synthesized Co_3_O_4_@crosslinked p[AVIM]Br as catalyst were dissolved in water and sonicated in an ultrasonic bath at 25 °C for appropriate times. The completion of the reaction was monitored by TLC and the catalyst was then magnetically separated. Finally, the crude precipitate was filtered and then recrystallized from EtOH to obtain a pure solid product. The products were characterized by ^1^H NMR, ^13^C NMR, IR, and elemental analysis (spectral data of compounds mentioned in ESI†).

### 3.7. Spectral data of the all compounds

#### 3′-Phenyl-spiro[3*H*-indole-3,2′-thiazolidine]-2,4′-(1*H*)-dione (4a)

Mp 233 °C (MeOH). ^1^H NMR (300 MHz, DMSO-d_6_) *δ*/ppm: 3.46 (d, 2H, CH_2_), 6.34–7.60 (m, 9H, Ar–H), 11.16 (s, 1H, NH). ^13^C NMR (100 MHz, DMSO-d_6_) *δ*/ppm: 172.1, 167.1, 142.4, 136.0, 134.0, 126.1, 125.2, 123.5, 119.7, 116.8, 108.7, 70.9, 31.2. Anal. calcd for C_16_H_12_N_2_O_2_S, % C, 64.85; H, 4.08; N, 9.45. Found, %: C, 64.84; H, 4.05; N, 9.43.

#### 3′-(4-Chlorophenyl)-spiro[3*H*-indole-3,2′-thiazolidine]-2,4′-(1*H*)-dione (4b)

Mp 170–173 °C (MeOH). ^1^H NMR (300 MHz, DMSO-d_6_) *δ*/ppm: 2.48 (2H, CH_2_), 6.75–7.22 (m, 8H, Ar–H), 10.38 (s, 1H, NH). ^13^C NMR (100 MHz, DMSO-d_6_) *δ*/ppm: 173.8, 170.9, 145.9, 133.9, 124.6, 123.9, 122.2, 120.0, 115.2, 113.5, 111.1, 71.8, 31.2. Anal. calcd for C_16_H_11_ClN_2_O_2_S, % C, 58.09; H, 3.35; N, 8.47. Found, %: C, 58.08; H, 3.33; N, 8.50.

#### 3′-(4-Methoxyphenyl)-spiro[3*H*-indole-3,2′-thiazolidine] 2,4′-(1*H*)-dione (4c)

Mp 168–170 °C. IR (CHCl_3_) *ν*_max_: 3280, 2833, 1738, 1612, 1500, 1462, 1333, 752 cm^−1^, ^1^H NMR (300 MHz, DMSO-d_6_) *δ*/ppm: 2.48 (s, 3H, CH_3_), 3.75–3.81 (q, 2H, CH_2_), 6.74–7.35 (m, 8H, Ar–H), 10.92 (s, 1H, NH). ^13^C NMR (100 MHz, DMSO-d_6_) *δ*/ppm: 176.83, 163.86, 156.92, 145.94, 133.90, 126.96, 123.93, 122.26, 120.37, 116.47, 115.20, 113.94, 87.96, 55.76, 32.96. Anal. calcd for C_17_H_14_N_2_O_3_S, % C, 62.56; H, 4.32; N, 8.58. Found, %: C, 62.57; H, 4.35; N, 8.60.

#### 3′-Benzyl-spiro[indoline-3,2′-thiazolidine]-2,4′-dione (4d)

Mp 180–182 °C (MeOH). ^1^H NMR (300 MHz, DMSO-d_6_) *δ*/ppm: 2.41–2.48 (2H, CH_2_), 3.71 (2H, CH_2_), 6.71–7.63 (m, 9H, Ar–H), 10.25 (s, 1H, NH). ^13^C NMR (100 MHz, DMSO-d_6_) *δ*/ppm: 177.6, 170.6, 144.3, 140.4, 134.6, 127.6, 125.4, 119.9, 115.1, 111.9, 75.3, 55.7, 22.2. Anal. calcd for C_17_H_14_N_2_O_2_S, % C, 65.79; H, 4.55; N, 9.03. Found, %: C, 65.78; H, 4.51; N, 9.01.

#### 3′-(4-Fluorophenyl)-spiro[3*H*-indole-3,2′-thiazolidine]-2,4′-(1*H*) dione (4e)

Mp 245–244 °C (MeOH). ^1^H NMR (300 MHz, DMSO-d_6_) *δ*/ppm: 3.78 (d, 1H, CH), 4.39 (d, 1H, CH), 6.69–7.56 (m, 8H, Ar–H), 9.97 (s, 1H, NH). 178.4, 167.9, 155.4, 151.2, 148.4, 140.5, 133.3, 132.4, 130.6, 126.8, 123.2, 122.6, 118.1, 116.5, 72.7, 36.5. Anal. calcd for C_16_H_11_FN_2_O_2_S, %: C, 61.14; H, 3.53; N, 8.91. Found, %: C, 61.11; H, 3.51; N, 8.90.

#### 3′-(5-Trifluoromethyl-2-chlorophenyl)-spiro-[3*H*-indole-3,2′-thiazolidine]-2,4′-(1*H*)-dione (4f)

Mp 145 °C (MeOH). ^1^H NMR (300 MHz, DMSO-d_6_) *δ*/ppm: 3.87 (d, 2H, CH_2_), 6.78–7.31 (m, 7H, Ar–H), 9.19 (s, 1H, NH). ^13^C NMR (75.47 MHz, DMSO-d_6_) *δ*/ppm: 179.9, 174.4, 146.0, 135.1, 134.1, 130.5, 129.2, 125.7, 125.0, 122.5, 120.4, 117.9, 113.5, 112.7, 100.0, 70.2, 21.0. Anal. calcd for C_17_H_10_ClF_3_N_2_O_2_S; % C, 51.20; H, 2.53; N, 7.02. Found C, 51.17; H, 2.52; N, 7.04.

#### 3′-(4-Hydroxyphenyl)-spiro[3*H*-indole-3,2′-thiazolidine]-2,4′-(1*H*)-dione (4g)

Mp > 230 °C (MeOH). ^1^H NMR (300 MHz, DMSO-d_6_) *δ*/ppm: 3.56 (2H, CH_2_), 6.51–7.63 (m, 8H, Ar–H), 9.04 (s, 1H, OH), 10.21 (s, 1H, NH). ^13^C NMR (100 MHz, DMSO-d_6_) *δ*/ppm: 180.1, 168.2, 162.2, 159.5, 143.2, 138.3, 136.3, 127.5, 124.9, 122.7, 112.8, 109.8, 108.9, 78.4, 33.0. Anal. calcd for C_16_H_12_N_2_O_3_S, % C, 61.53; H, 3.87; N, 8.97. Found, %: C, 61.55; H, 3.85; N, 9.00.

#### 5-Chloro-3′-phenylspiro[indoline-3,2′-thiazolidine]-2,4′-dione (4h)

Mp 306 °C (MeOH). ^1^H NMR (300 MHz, DMSO-d_6_) *δ*/ppm: 4.03 (d, 2H, CH_2_), 6.76–7.69 (m, 8H, Ar–H), 10.90 (s, 1H, NH). ^13^C NMR (100 MHz, DMSO-d_6_) *δ*/ppm: 176.2, 171.9, 140.4, 136.2, 131.3, 129.6, 128.7, 128.2, 127.2, 127.0, 126.6, 112.4, 69.6, 32.5. Anal. calcd for C_16_H_11_ClN_2_O_2_S, % C, 58.09; H, 3.35; N, 8.47. Found, %: C, 58.05; H, 3.35; N, 8.42.

#### 3′-(4-Methyl)-5-chlorospiro[3*H*-indole-3,2′-thiazolidine]-2,4′-(1*H*)-dione (4i)

Mp 185–186 °C (MeOH). ^1^H NMR (300 MHz, DMSO-d_6_) *δ*/ppm: 2.35 (s, 3H, CH_3_), 3.30 (d, 2H, CH_2_), 6.38–7.40 (m, 7H, Ar–H), 11.07 (s, 1H, NH). ^13^C NMR (100 MHz, DMSO-d_6_) *δ*/ppm: 179.5, 161.7, 159.0, 152.9, 149.5, 143.3, 137.7, 127.6, 125.0, 123.5, 111.1, 109.7, 76.3, 50.8, 32.1. Anal. calcd for C_17_H_13_ClN_2_O_2_S, % C, 59.21; H, 3.80; N, 8.12. Found, %: C, 59.25; H, 3.82; N, 8.12.

#### 3′-Benzyl-5-chlorospiro[indoline-3,2′-thiazolidine]-2,4′-dione (4j)

Mp 186–188 °C (MeOH). ^1^H NMR (300 MHz, DMSO-d_6_) *δ*/ppm: 3.31 (d, 2H, benzyl CH_2_), 4.00 (d, 2H, CH_2_), 6.71–8.00 (m, 8H, Ar–H), 10.54 (s, 1H, NH). ^13^C NMR (100 MHz, DMSO-d_6_) *δ*/ppm: 175.8, 172.1, 142.0, 135.5, 131.3, 128.08, 128.0, 127.4, 126.3, 123.6, 122.3, 110.6, 68.5, 46.1, 32.1. Anal. calcd for C_17_H_13_ClN_2_O_2_S, % C, 59.21; H, 3.80; N, 8.12. Found, %: C, 59.20; H, 3.78; N, 8.11.

#### 3′-(4-Methoxyphenyl)-5-chloro-3′-phenylspiro[3*H*-indole-3,2′-thiazolidine]-2,4′-(1*H*)-dione (4k)

Mp 186–188 °C (MeOH). IR (CHCl_3_) *ν*_max_: 3448, 3224, 2931, 2835, 1736, 1608, 1597, 1504, 1462, 1292, 1254, 1034, 837 cm^−1^, ^1^H NMR (300 MHz, DMSO-d_6_) *δ*/ppm: 2.48 (s, 3H, CH_3_), 3.79 (q, 2H, CH_2_), 6.83–7.41 (m, 7H, Ar–H), 11.08 (s, 1H, NH). ^13^C NMR (100 MHz, DMSO-d_6_) *δ*/ppm: 173.8, 163.8, 157.9, 145.9, 133.9, 124.6, 123.9, 122.2, 120.0, 115.2, 113.9, 87.9, 55.8, 32.5. Anal. calcd for C_17_H_13_ClN_2_O_3_S, % C, 56.59; H, 3.63; N, 7.76. Found, %: C, 56.57; H, 3.62; N, 7.74.

#### 5-Bromo-3′-phenylspiro[indoline-3,2′-thiazolidine]-2,4′-dione (4l)

Mp 306 °C (MeOH). ^1^H NMR (300 MHz, DMSO-d_6_) *δ*/ppm: 3.8 (s, 2H, CH_2_), 6.92–8.16 (m, 8H, Ar–H), 11.17 (s, 1H, NH). ^13^C NMR (100 MHz, DMSO-d_6_) *δ*/ppm: 179.6, 174.4, 154.9, 140.4, 135.0, 134.6, 129.6, 125.2, 119.4, 114.1, 111.2, 110.9, 72.1, 38.5. Anal. calcd for C_16_H_11_BrN_2_O_2_S, % C, 51.21; H, 2.95; N, 7.47. Found, %: C, 51.25; H, 2.93; N, 7.42.

#### 3′-Benzyl-5-bromospiro[indoline-3,2′-thiazolidine]-2,4′-dione (4m)

Mp 190–192 °C (MeOH). ^1^H NMR (300 MHz, DMSO-d_6_) *δ*/ppm: 3.31 (d, 2H, benzyl CH_2_), 4.00 (d, 2H, CH_2_), 6.71–8.00 (m, 8H, Ar–H), 10.54 (s, 1H, NH). ^13^C NMR (100 MHz, DMSO-d_6_) *δ*/ppm: 178.5, 175.3, 152.3, 148.0, 146.3, 136.2, 135.9, 130.9, 125.3, 124.2, 115.0, 108.8, 80.6, 38.9, 31.1. Anal. calcd for C_17_H_13_ClN_2_O_2_S, % C, 59.21; H, 3.80; N, 8.12. Found, %: C, 59.20; H, 3.78; N, 8.11.

#### 5-Methyl-3′-phenylspiro[indoline-3,2′-thiazolidine]-2,4′-dione (4n)

Mp > 300 °C (MeOH). ^1^H NMR (300 MHz, DMSO-d_6_) *δ*/ppm: 2.08 (s, 3H, CH_3_), 3.25 (d, 2H, CH_2_), 6.47–8.06 (m, 8H, Ar–H), 10.94 (s, 1H, NH). ^13^C NMR (100 MHz, DMSO-d_6_) *δ*/ppm: 180.0, 170.8, 157.0, 154.0, 146.8, 143.6, 125.8, 121.1, 118.3, 117.8, 114.9, 108.7, 102.9, 71.8, 29.2. Anal. calcd for C_17_H_14_N_2_O_2_S, % C, 65.79; H, 4.55; N, 9.03. Found, %: C, 65.75; H, 4.54; N, 9.02.

#### 3′-(4-Methyl)-5-methylspiro[3*H*-indole-3,2′-thiazolidine]-2,4′-(1*H*)-dione (4o)

Mp 187–189 °C (MeOH). ^1^H NMR (300 MHz, DMSO-d_6_) *δ*/ppm: 1.68 (s, 3H, CH_3_), 2.48 (s, 3H, CH_3_), 3.76 (d, 2H, CH_2_), 6.56–7.41 (m, 7H, Ar–H), 11.08 (s, 1H, NH). ^13^C NMR (100 MHz, DMSO-d_6_) *δ*/ppm: 177.4, 165.9, 152.4, 150.4, 148.4, 141.7, 134.1, 133.0, 130.0, 124.8, 124.2, 121.6, 117.3, 116.6, 71.7, 36.5, 31.0. Anal. calcd for C_18_H_16_N_2_O_2_S, % C, 66.64; H, 4.97; N, 8.64. Found, %: C, 66.65; H, 4.82; N, 8.62.

#### 3′-(4-Fluorophenyl)-5-methylspiro[3*H*-indole-3,2′-thiazolidine]-2,4′-(1*H*)-dione (4p)

Mp 190–192 °C (MeOH). ^1^H NMR (300 MHz, DMSO-d_6_) *δ*/ppm: 1.68 (s, 3H, CH_3_), 2.68 (d, 2H, CH_2_), 6.71–7.28 (m, 7H, ArH), 11.66 (s, 1H, NH). ^13^C NMR (100 MHz, DMSO-d_6_) *δ*/ppm: 20.9, 24.6, 32.6, 73.1, 116.08–140.34, 150.8, 167.2, 170.5, 118.02, 63.05. Anal. calcd for C_17_H_13_FN_2_O_2_S, % C, 62.18; H, 3.99; N, 8.53. Found, %: C, 62.15; H, 3.88; N, 8.52.

#### 3′-(2,3-Dihydro-1,5-dimethyl-3-oxo-2-phenyl-1*H*-pyrazol-4-yl)-spiro[3*H*-indole-3,2′-thiazolidine]-2,4′-(1*H*) dione (4q)

Mp 320 °C (MeOH). ^1^H NMR (300 MHz, DMSO-d_6_) *δ*/ppm: 2.50 (s, 3H, CH_3_), 3.41 (s, 3H, N–CH_3_), 4.05 (d, 2H, CH_2_), 6.70–8.12 (m, 9H, Ar–H), 10.63 (s, 1H, NH). ^13^C NMR (100 MHz, DMSO-d_6_) *δ*/ppm: 175.8, 173.8, 165.6, 155.8, 148.4, 144.3, 143.8, 133.2, 131.7, 121.9, 121.0, 115, 9, 108.8, 72.4, 29.6, 22.0, 15.5. Anal. calcd for C_21_H_18_N_4_O_3_S; % C, 62.05; H, 4.46; N, 13.78. Found % C, 62.09; H, 4.45; N, 13.79.

#### 5-Bromo-3′-(2,3-dihydro-1,5-dimethyl-3-oxo-2-phenyl-1*H*-pyrazol-4-yl)-spiro[3*H*-indole-3,2′-thiazolidine]-2,4′-(1*H*) dione (4r)

Mp 333 °C (MeOH). ^1^H NMR (300 MHz, DMSO-d_6_) *δ*/ppm: 2.50 (s, 3H, CH_3_), 3.04 (s, 3H, N–CH_3_), 3.99 (d, 2H, CH_2_), 6.76–7.79 (m, 8H, Ar–H), 10.93 (s, 1H, NH). ^13^C NMR (100 MHz, DMSO-d_6_) *δ*/ppm: 178.2, 173.7, 169.9, 163.8, 162.3, 159.4, 157.1, 154.7, 148.5, 146.8, 131.1, 129.8, 128.3, 126.6, 124.3, 123.2, 70.1, 37.4, 17.0, 16.7. Anal. calcd for C_21_H_17_BrN_4_O_3_S; % C, 51.97; H, 3.53; N, 11.54. Found % C, 51.89; H, 3.56; N, 11.59.

#### 5-Methyl-3′-(2,3-dihydro-1,5-dimethyl-3-oxo-2-phenyl-1*H*-pyrazol-4-yl)-spiro[3*H*-indole-3,2′-thiazolidine]-2,4′-(1*H*) dione (4s)

Mp 328–330 °C (MeOH). ^1^H NMR (300 MHz, DMSO-d_6_) *δ*/ppm: 2.67 (s, 3H, CH_3_), 2.73 (s, 3H, N–CH_3_), 4.44 (d, 2H, CH_2_), 6.74–7.97 (m, 8H, Ar–H), 10.65 (s, 1H, NH). ^13^C NMR (100 MHz, DMSO-d_6_) *δ*/ppm: 177.7, 173.6, 165.4, 161.7, 157.8, 152.0, 149.3, 132.2, 131.9, 129.8, 129.7, 129.2, 124.8, 116.0, 115.6, 97.9, 69.5, 49.0, 31.1, 18.1, 14.2. Anal. calcd for C_22_H_20_N_4_O_3_S; % C, 62.84; H, 4.79; N, 13.32. Found % C, 62.85; H, 4.76; N, 13.39.

#### 3′-(1*H*-Benzimidazol-2-yl)-spiro-[3*H*-indole-3,2′-thiazolidine]-2,4′-(1*H*)-dione (4t)

Mp 250 °C (MeOH). ^1^H NMR (300 MHz, DMSO-d_6_) *δ*/ppm: 4.81 (d, 2H, CH_2_), 6.72–8.35 (m, 8H, Ar–H), 10.75 (s, 1H, NH), 10.89 (s, 1H, NH). ^13^C NMR (100 MHz, DMSO-d_6_) *δ*/ppm: 177.6, 167.6, 157.8, 146.6, 141.5, 137.6, 131.0, 129.5, 129.3, 128.4, 126.0, 120.9, 104.9, 72.9, 32.9. Anal. calcd for C_17_H_12_N_4_O_2_S; % C, 60.70; H, 3.60; N, 16.66. Found C, 60.71; H, 3.55; N, 16.69.

#### 5-Chloro-3′-(1*H*-benzimidazol-2-yl)-spiro-[3*H*-indole-3,2′-thiazolidine]-2,4′-(1*H*)-dione (4u)

Mp 265 °C (MeOH). ^1^H NMR (300 MHz, DMSO-d_6_) *δ*/ppm: 3.38 (d, 1H, CH), 4.39 (d, 1H, CH), 6.72–7.78 (m, 7H, Ar–H), 10.12 (s, 1H, NH), 12.89 (s, 1H, NH). ^13^C NMR (100 MHz, DMSO-d_6_) *δ*/ppm: 171.4, 167.6, 150.9, 145.0, 139.2, 132.0, 130.2, 128.0, 127.2, 126.0, 123.8, 122.4, 118.0, 108.0, 65.3, 29.8. Anal. calcd for C_17_H_11_ClN_4_O_2_S; % C, 55.06; H, 2.99; N, 15.11. Found C, 55.01; H, 2.97; N, 15.09.

#### 5-Methyl-3′-(1*H*-benzimidazol-2-yl)-spiro-[3*H*-indole-3,2′-thiazolidine]-2,4′-(1*H*)-dione (4v)

Mp 240 °C (MeOH). ^1^H NMR (300 MHz, DMSO-d_6_) *δ*/ppm: 2.40 (s, 3H, CH_3_), 4.81 (d, 2H, CH_2_), 6.44–8.35 (m, 7H, ArH), 10.74 (s, 1H, NH), 10.98 (s, 1H, NH). ^13^C NMR (100 MHz, DMSO-d_6_) *δ*/ppm: 174.2, 166.2, 164.4, 164.1, 155.7, 155.4, 152.3, 150.5, 132.7, 128.4, 127.7, 124.7, 117.4, 116.5, 115.3, 105.4, 77.5, 35.7, 30.9. Anal. calcd for C_18_H_14_N_4_O_2_S, % C, 61.70; H, 4.03; N, 15.99. Found, %: C, 61.65; H, 4.01; N, 15.92.

#### 3′-(1,3-Benzothiazol-2-yl)-spiro-[3*H*-indole-3,2′-thiazolidine]2,4′-(1*H*)-dione (4w)

Mp 168 °C (MeOH). ^1^H NMR (300 MHz, DMSO-d_6_) *δ*/ppm: 4.41 (d, 1H, CH), 4.86 (d, 1H, CH), 6.97–7.65 (m, 8H, Ar–H), 10.95 (s, 1H, NH). ^13^C NMR (100 MHz, DMSO-d_6_) *δ*/ppm: 175.6, 170.6, 156.8, 143.6, 141.5, 136.6, 130.0, 129.5, 127.3, 126.4, 126.0, 121.9, 108.9, 74.9, 32.1. Anal. calcd for C_17_H_11_N_3_O_2_S_2_; C, 57.77; H, 3.14; N, 11.89. Found C, 57.70; H, 3.13; N, 11.85.

#### 5-Chloro-3′-(1,3-benzothiazol-2-yl)-spiro-[3*H*-indole-3,2′-thiazolidine]-2,4′-(1*H*)-dione (4x)

Mp 171 °C (MeOH). ^1^H NMR (300 MHz, DMSO-d_6_) *δ*/ppm: 4.47 (d, 1H, CH), 4.84 (d, 1H, CH), 6.89–7.75 (m, 7H, Ar–H), 10.59 (s, 1H, NH). ^13^C NMR (100 MHz, DMSO-d_6_) *δ*/ppm: 175.5, 169.3, 159.2, 144.6, 142.2, 137.7, 136.9, 132.8, 129.0, 128.1, 127.0, 123.7, 122.6, 122.3, 107.7, 65.0, 25.7. Anal. calcd for C_17_H_10_ClN_3_O_2_S_2_; % C, 52.64; H, 2.60; N, 10.83. Found % C, 52.70; H, 2.62; N, 10.86.

#### 5-Methyl-3′-(1,3-benzothiazol-2-yl)-spiro-[3*H*-indole-3,2′-thiazolidine]-2,4′-(1*H*)-dione (4y)

Mp 163 °C (MeOH). ^1^H NMR (300 MHz, DMSO-d_6_) *δ*/ppm: 2.24 (s, 3H, CH_3_), 3.87 (d, 1H, CH), 4.51 (d, 1H, CH), 7.06–7.66 (m, 7H, Ar–H), 8.30 (s, 1H, NH). ^13^C NMR (100 MHz, DMSO-d_6_) *δ*/ppm: 178.4, 169.9, 160.6, 141.9, 140.7, 134.7, 132.2, 131.4, 130.6, 129.5, 129.0, 127.7, 128.4, 123.7, 122.2, 66.0, 20.9. Anal. calcd for C_18_H_13_N_3_O_2_S_2_; % C, 58.84; H, 3.57; N, 11.44. Found % C, 58.76; H, 3.55; N, 11.42.

#### 3′-(6-Chloro-1,3-benzothiazol-2-yl)-spiro-[3*H*-indole-3,2′-thiazolidine]-2,4′-(1*H*)-dione (4z)

Mp 170 °C (MeOH). ^1^H NMR (300 MHz, DMSO-d_6_) *δ*/ppm: 3.43 (d, 2H, CH_2_), 6.93–8.21 (m, 7H, Ar–H), 10.87 (s, 1H, NH). ^13^C NMR (100 MHz, DMSO-d_6_) *δ*/ppm: 175.8, 171.7, 157.2, 154.2, 147.8, 143.1, 127.8, 125.1, 120.7, 118.2, 117.8, 112.1, 102.7, 70.0, 21.2. Anal. calcd for C_17_H_10_ClN_3_O_2_S_2_; % C, 52.64; H, 2.60; N, 10.83. Found % C, 52.70; H, 2.62; N, 10.86.

## 4. Conclusions

In this study, a nanoporous poly (ionic liquid) is proposed to facilitate the synthesis of the pharmaceutical spiro-4-thiazolidinone library. In practice, two different methods were applied to study the effect of polymerization protocol on morphology and efficiency of prepared sonocatalyst. According to the obtained results, hydrothermal precipitation polymerization provided a higher degree of cross linking and as a result achieved more thermal stability, and porous structure (smaller pore size distribution) which are known as the key characteristic parameters in the catalysis field. On the other hand, the synergistic effect between US irradiation and magnetically separable catalyst play a vital role in the straightforward synthesis of spiro-4-thiazolidinones.

## Conflicts of interest

There are no conflicts to declare.

## Supplementary Material

RA-010-D0RA08647A-s001
