# Supplementary material for: The influence of the polymerization approach on the catalytic performance of novel porous poly (ionic liquid)s for green synthesis of pharmaceutical spiro-4-thiazolidinones
Source: RSC Adv. 2020 Dec 15;10(72):44159–70. doi: 10.1039/d0ra08647a (PMC9058518; doi:10.1039/d0ra08647a)
Supplement: RA-010-D0RA08647A-s001 [file RA-010-D0RA08647A-s001.pdf]

Electronic Supplementary Material (ESI) for RSC Advances.
This journal is © The Royal Society of Chemistry 2020

The influence of polymerization approach on the catalytic performance of novel porous poly (ionic liquid) for green synthesis of pharmaceutical spiro-4-thiazolidinones

Zahra Elyasi[a], Javad Safaei Ghomi*[a,b], Gholam Reza Najafi[a], Mohammad Reza Zand Monfared[a]

[a] Department of Chemistry, Qom branch, Islamic azad University, Qom, I. R. Iran
[b] Department of Organic Chemistry, Faculty of Chemistry, University of Kashan
* Corresponding author: +98 31 55912385; fax: +98 31 55552935; *E-mail: safaei@kashanu.ac.ir

Table of Contents

Part 1. Characterization of ionic monomer S2

Part 2. Characterization of fabricated PIL:

EDX analysis and elemental mapping images S3

VSM analysis S4

Part 3. Characterization of spiro-4-thiazolidinones: ^{1}H NMR, ^{13}C NMR,

and elemental analysis S5-S32

Part 1. Characterization of ionic monomer

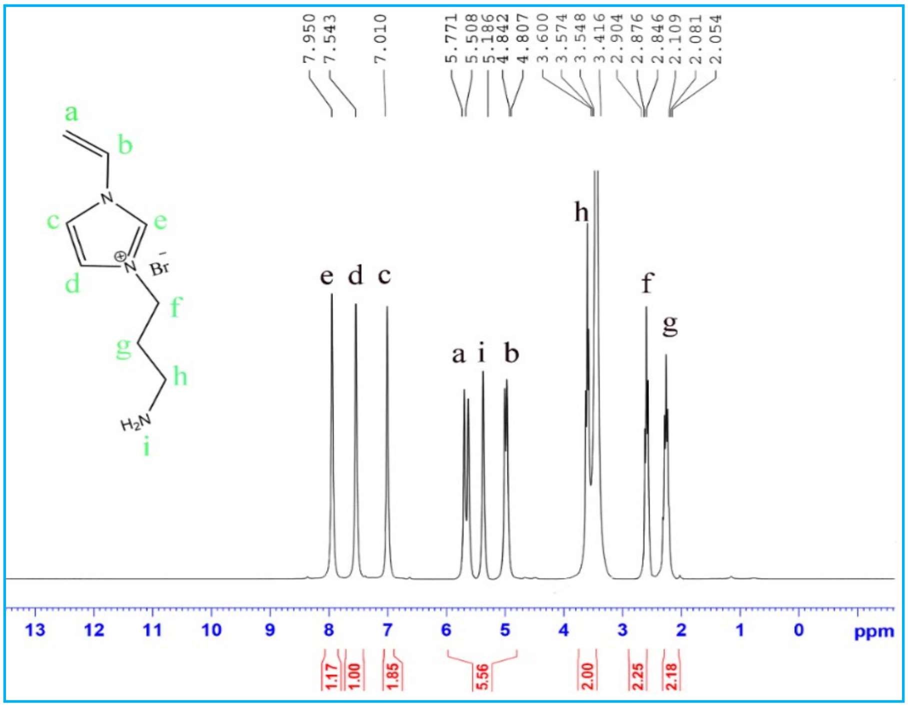

Figure S1. ^{1}H NMR spectra of ionic monomer

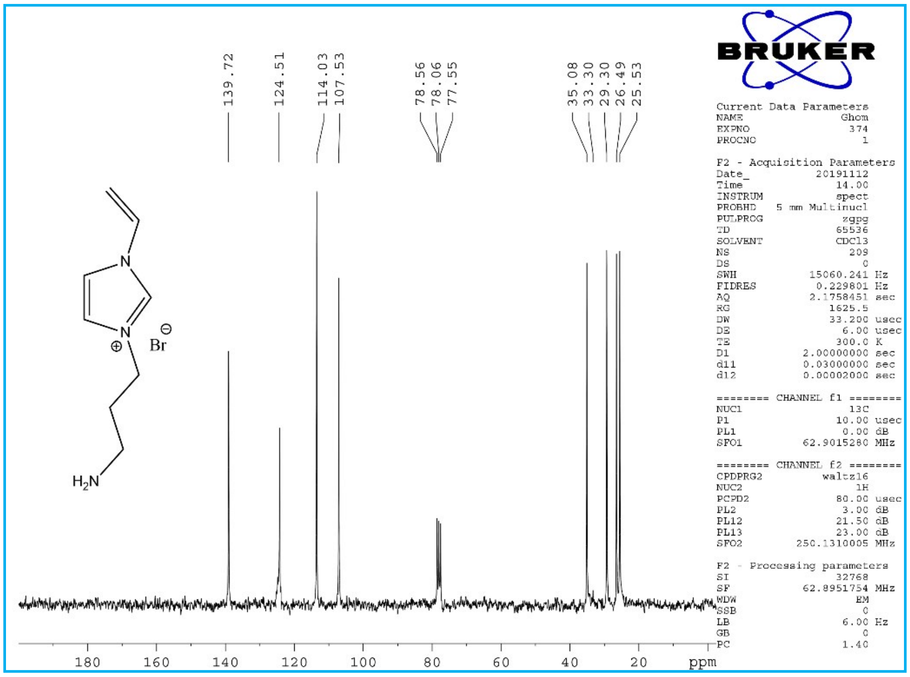

Figure S2. ^{13}C NMR spectra of ionic monomer

Part 2. EDX analysis and elemental mapping images

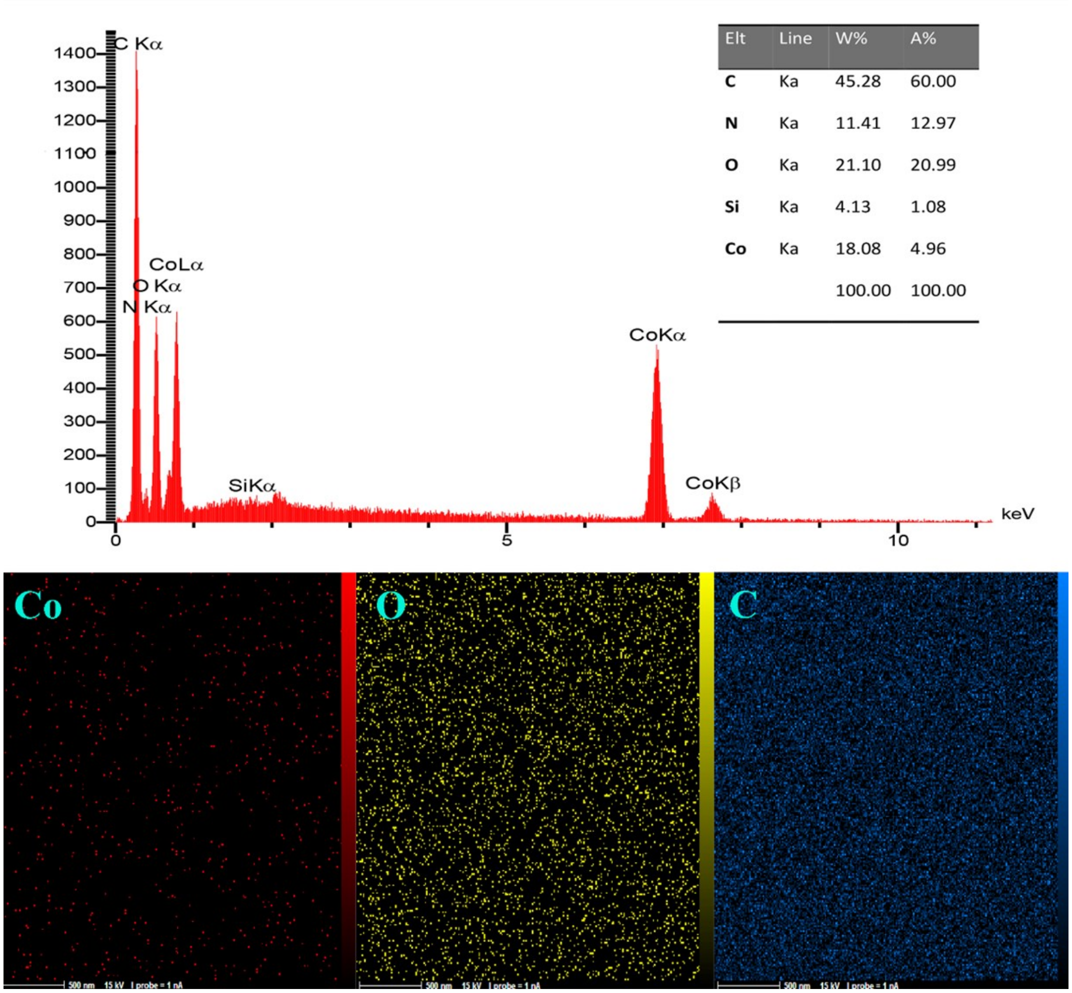

Figure S3. EDX analysis and elemental mapping images of Co_3O_4@crosslinked p[AVIM]Br

Part 2. VSM analysis

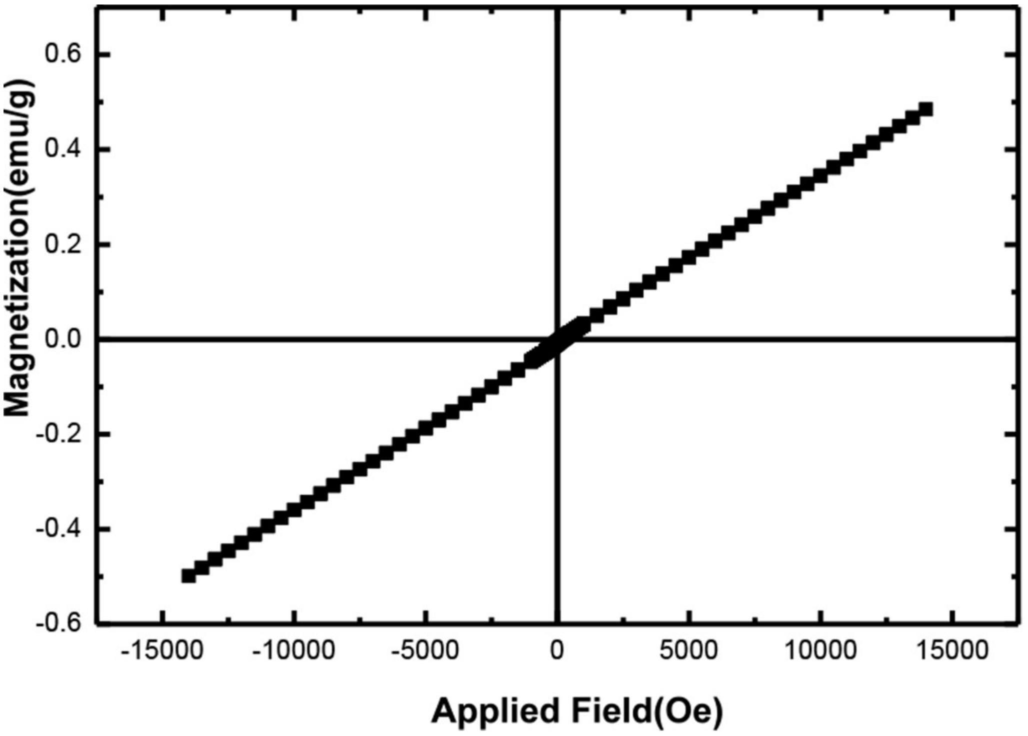

Figure S4. VSM curve of sonocatalyst

Part 3. Characterization of spiro-4-thiazolidinones

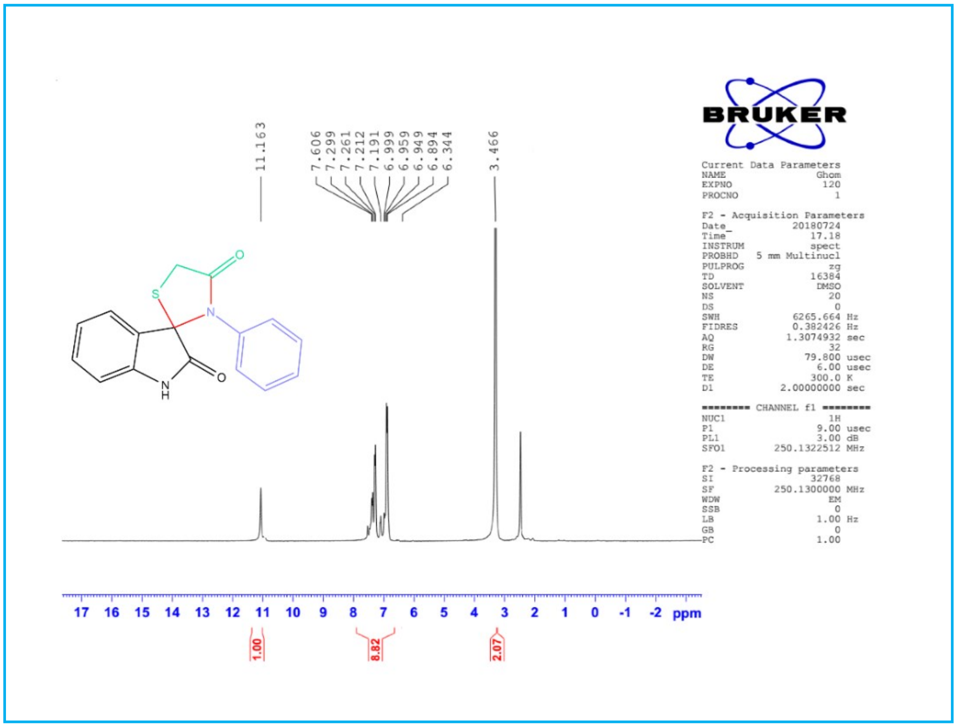

Figure S5. ^{1}H NMR spectrum of 3`-Phenyl-spiro[3H-indole-3,2`-thiazolidine]-2,4`(1H)-dione (**4a**)

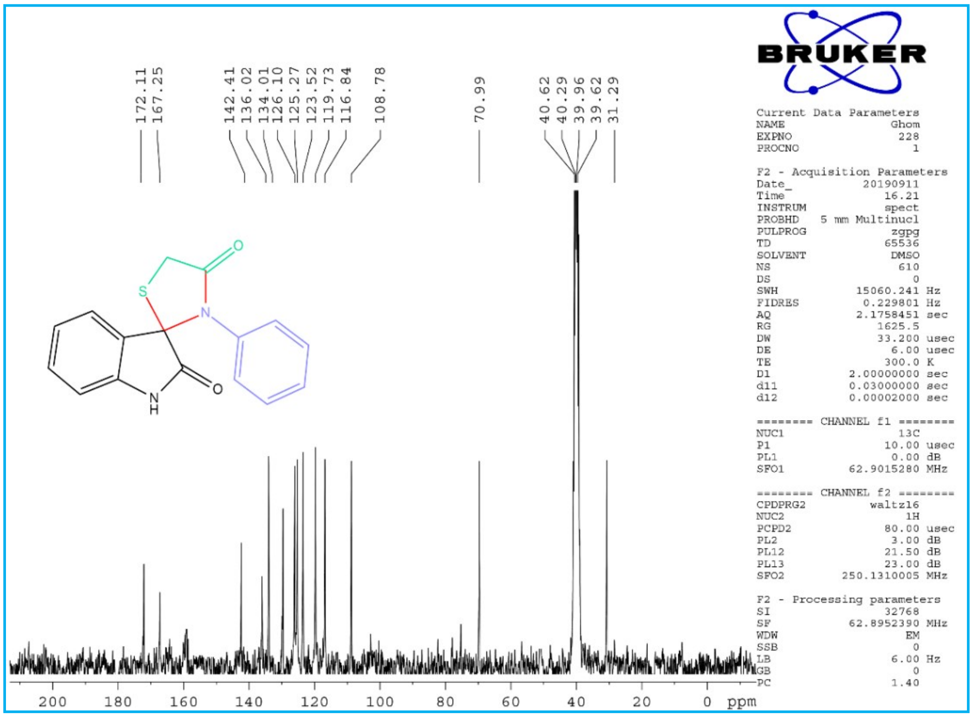

Figure S6. ^{13}C NMR spectrum of 3`-Phenyl-spiro[3H-indole-3,2`-thiazolidine]-2,4`(1H)-dione (**4a**)

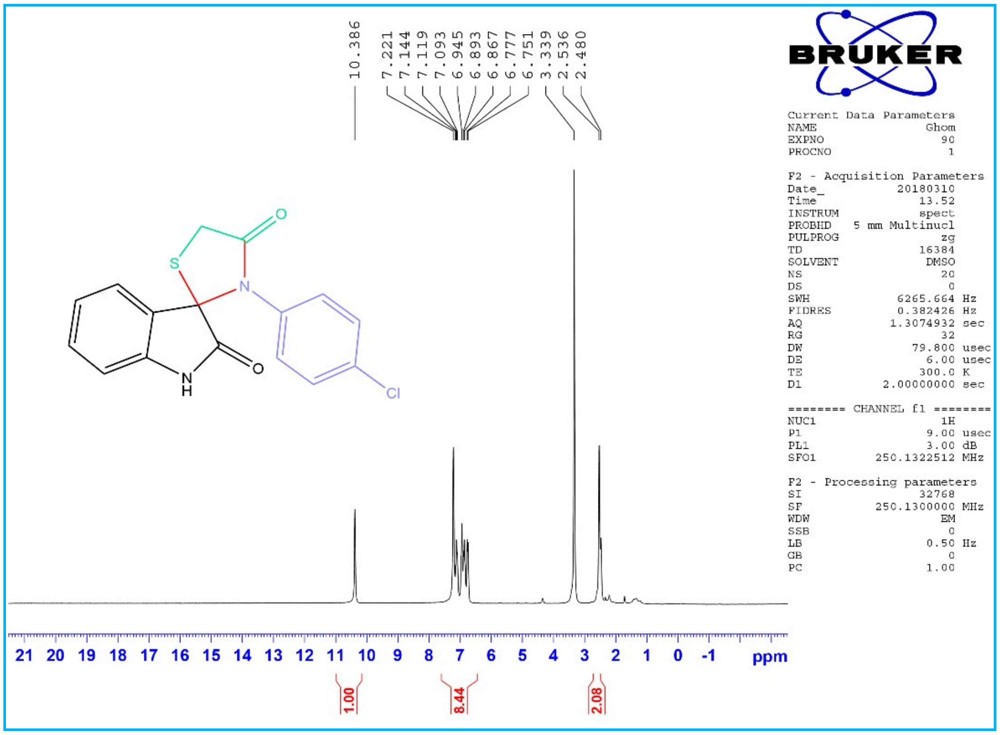

Figure S7. ^{1}H NMR spectrum of 3’-(4-Chlorophenyl)-spiro[3H-indole-3,2’-thiazolidine]-2,4'(1H)-dione (**4b**)

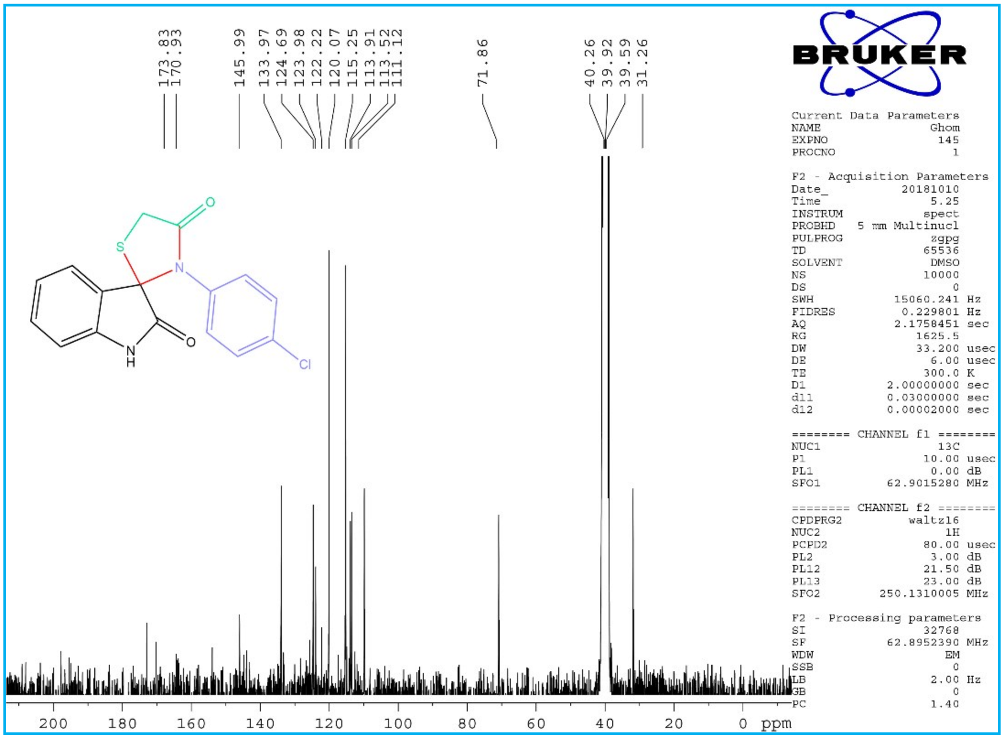

Figure S8. ^{13}C NMR spectrum of 3’-(4-Chlorophenyl)-spiro[3H-indole-3,2’-thiazolidine]-2,4'(1H)-dione (**4b**)

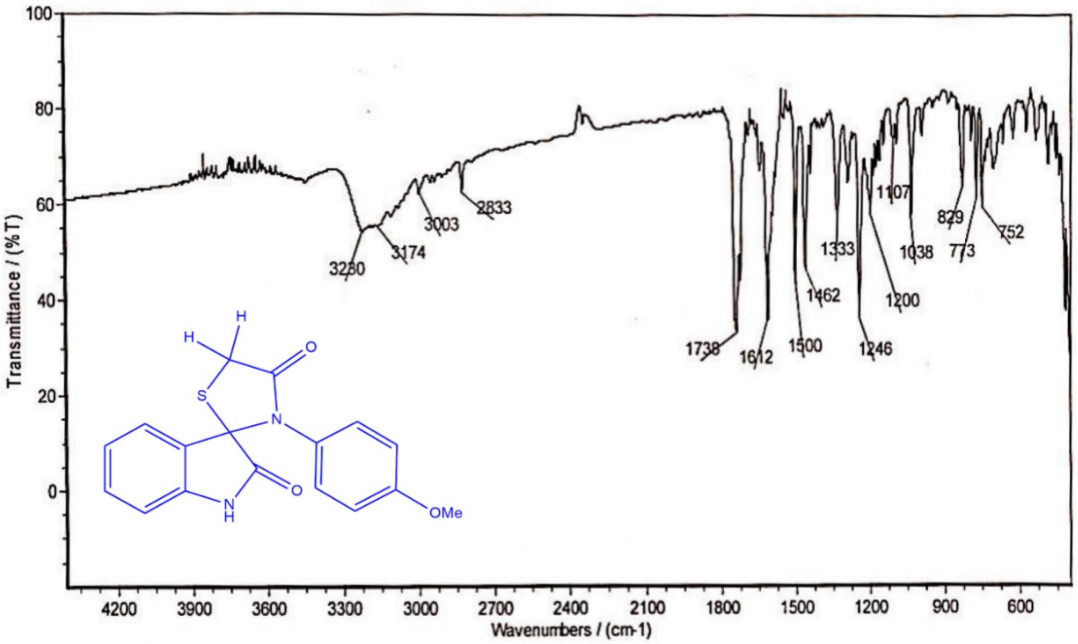

New compound, Figure S9: FT-IR (KBr) spectrum of 3’-(4-Methoxyphenyl)-spiro[3H-indole-3,2’-thiazolidine]-2,4'(1H)-dione (**4c**)

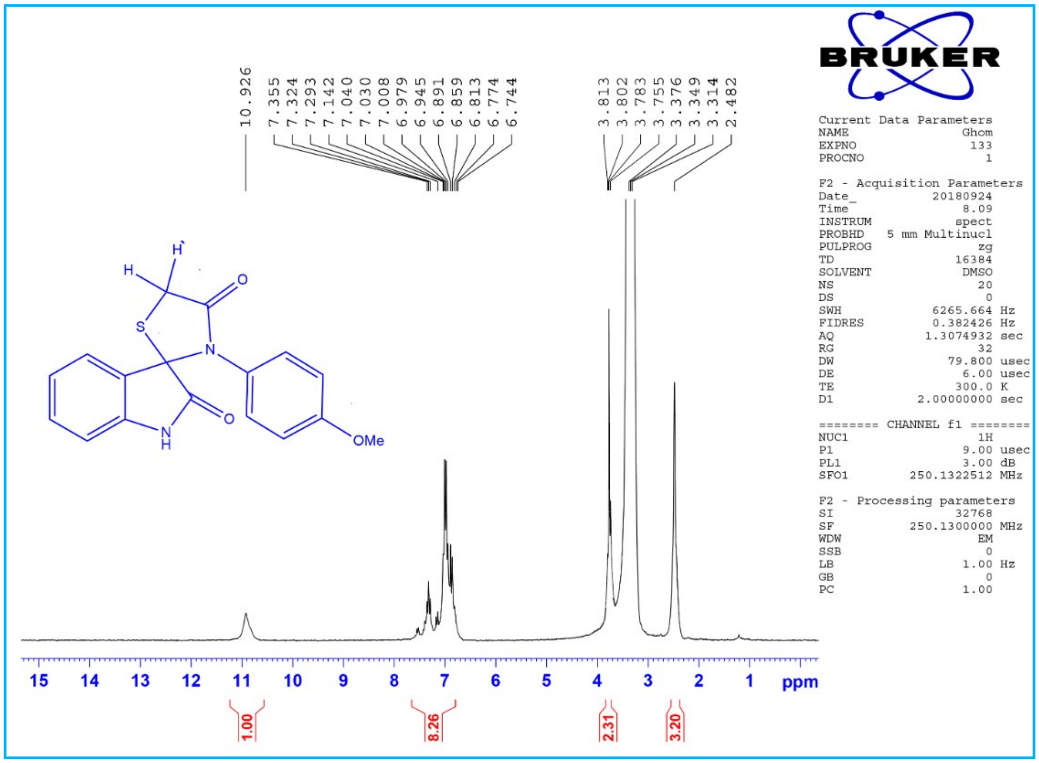

New compound, Figure S10: ^{1}H NMR spectrum of 3’-(4-Methoxyphenyl)-spiro[3H-indole-3,2’-thiazolidine]-2,4'(1H)-dione (**4c**)

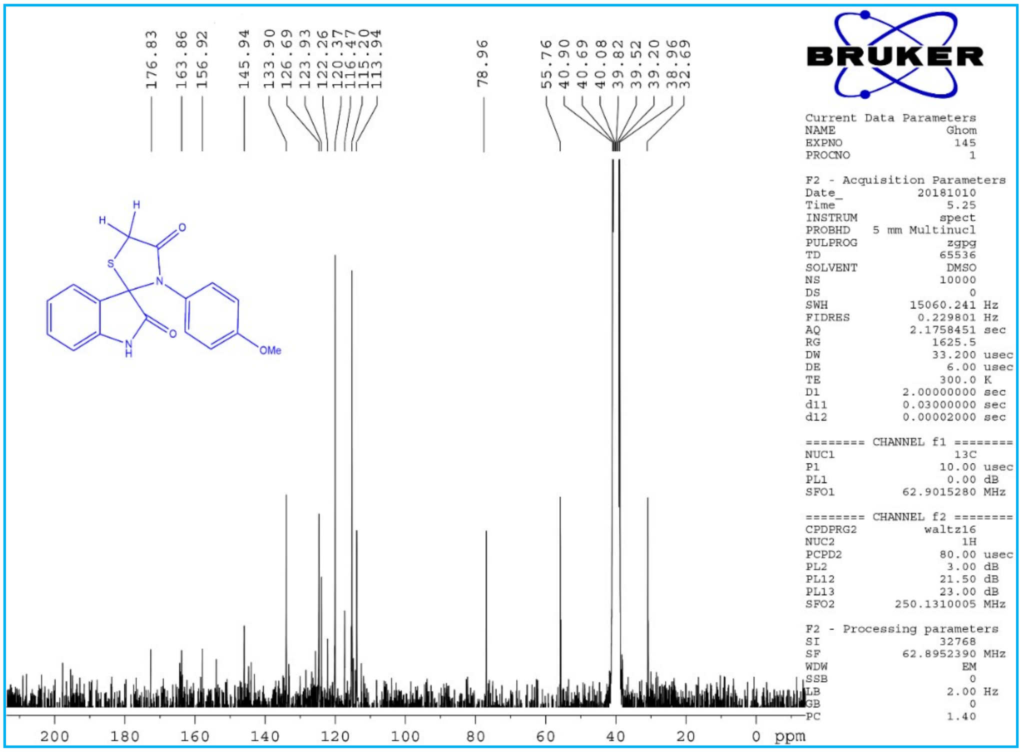

New compound, Figure S11: ^{13}C NMR spectrum of 3’-(4-Methoxyphenyl)-spiro[3H-indole-3,2’-thiazolidine]-2,4'(1H)-dione (**4c**)

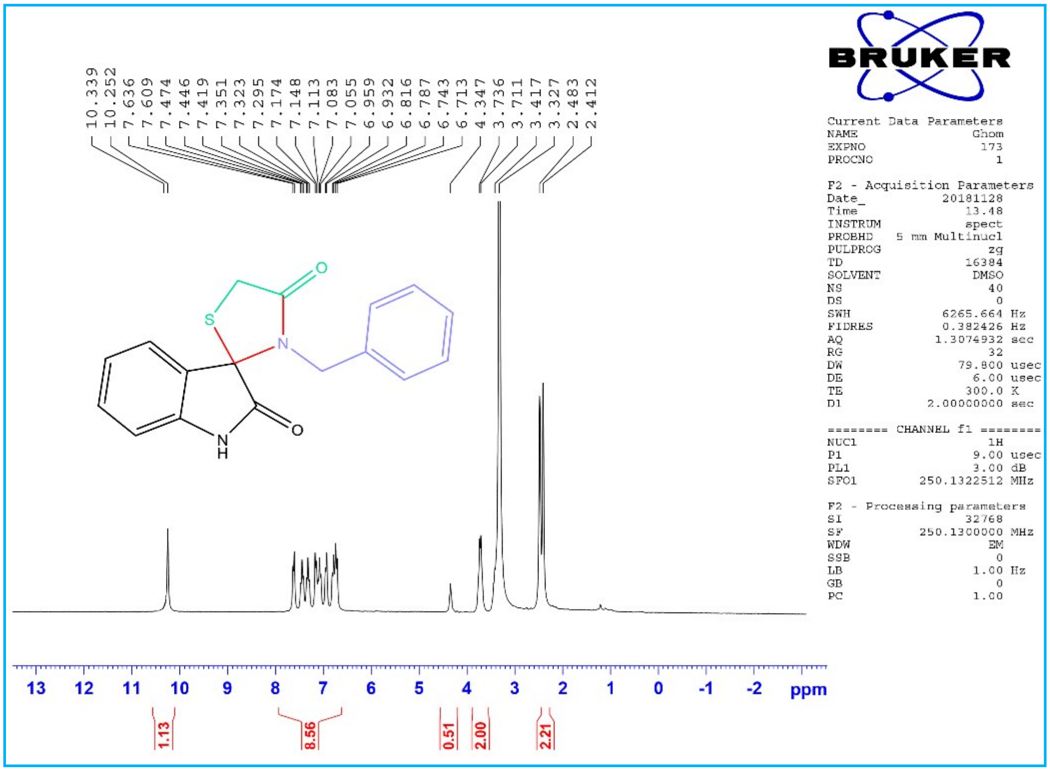

Figure S12: ^{1}H NMR spectrum of 3′-Benzyl-spiro[indoline-3,2′-thiazolidine]-2,4′-dione (**4d**)

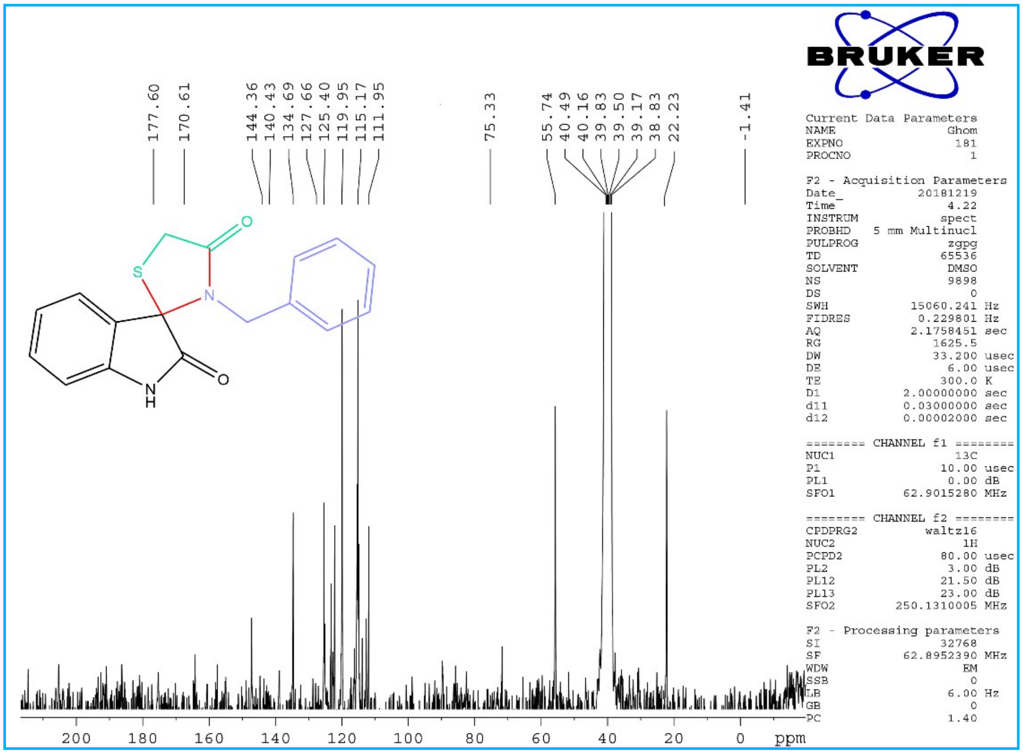

Figure S13: ^{13}C NMR spectrum of 3′-Benzyl-spiro[indoline-3,2′-thiazolidine]-2,4′-dione (**4d**)

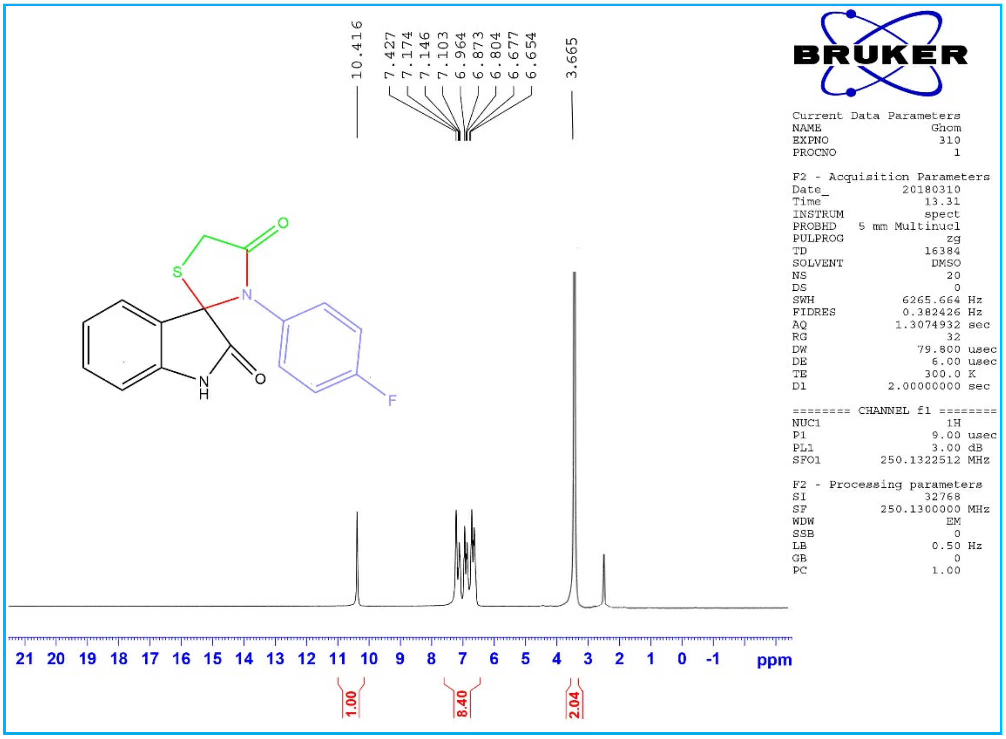

Figure S14: ^{1}H NMR spectrum of 3`-(4-Fluorophenyl)-spiro[3H-indole-3,2`-thiazolidine]-2,4`(1H) dione (**4e**)

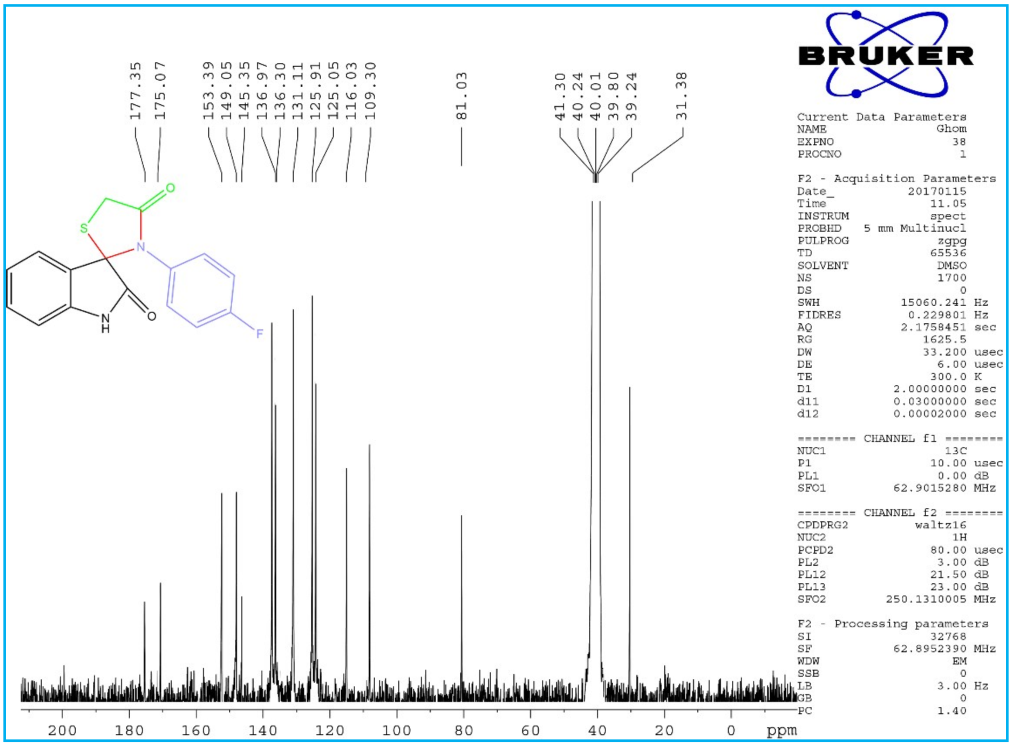

Figure S15: ^{13}C NMR spectrum of 3`-(4-Fluorophenyl)-spiro[3H-indole-3,2`-thiazolidine]-2,4`(1H) dione (**4e**)

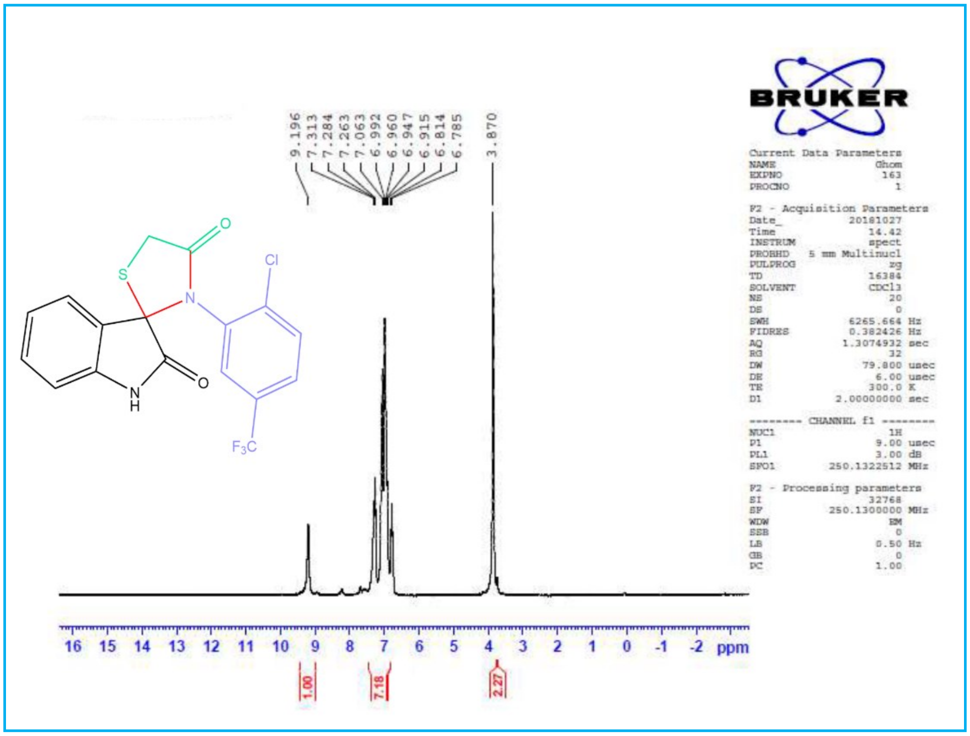

Figure S16: ^{1}H NMR spectrum of 3`-(5-Trifluoromethyl-2-chlorophenyl)-spiro-[3H-indole3,2`-thiazolidine]-2,4`(1H)-dione (**4f**)

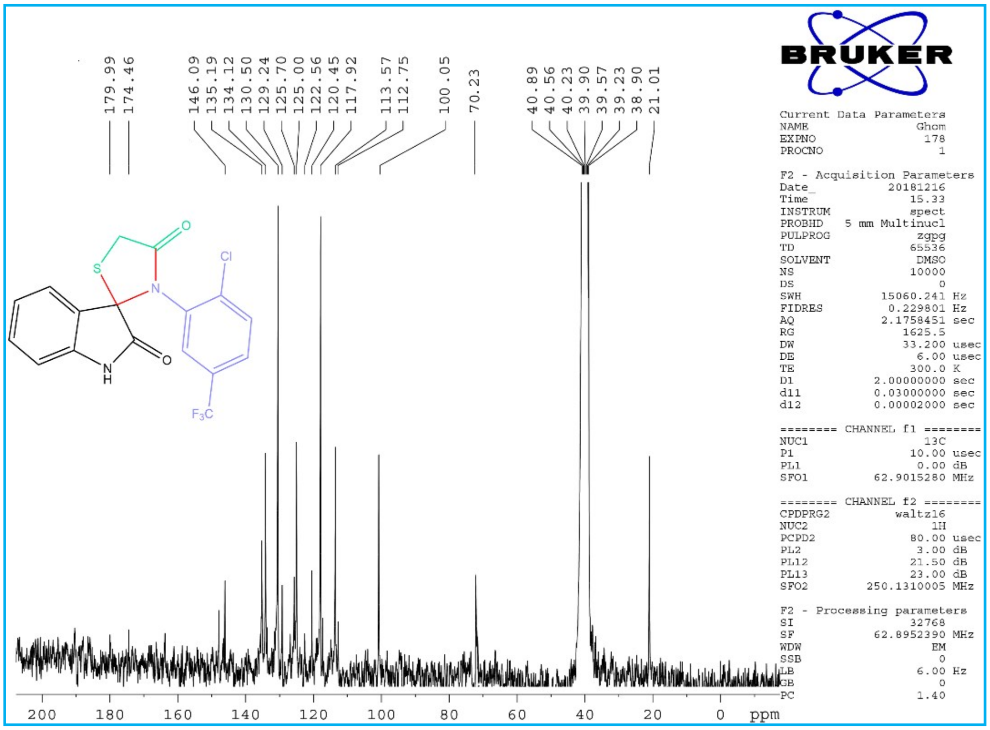

Figure S17: ^{13}C NMR spectrum of 3`-(5-Trifluoromethyl-2-chlorophenyl)-spiro-[3H-indole3,2`-thiazolidine]-2,4`(1H)-dione (**4f**)

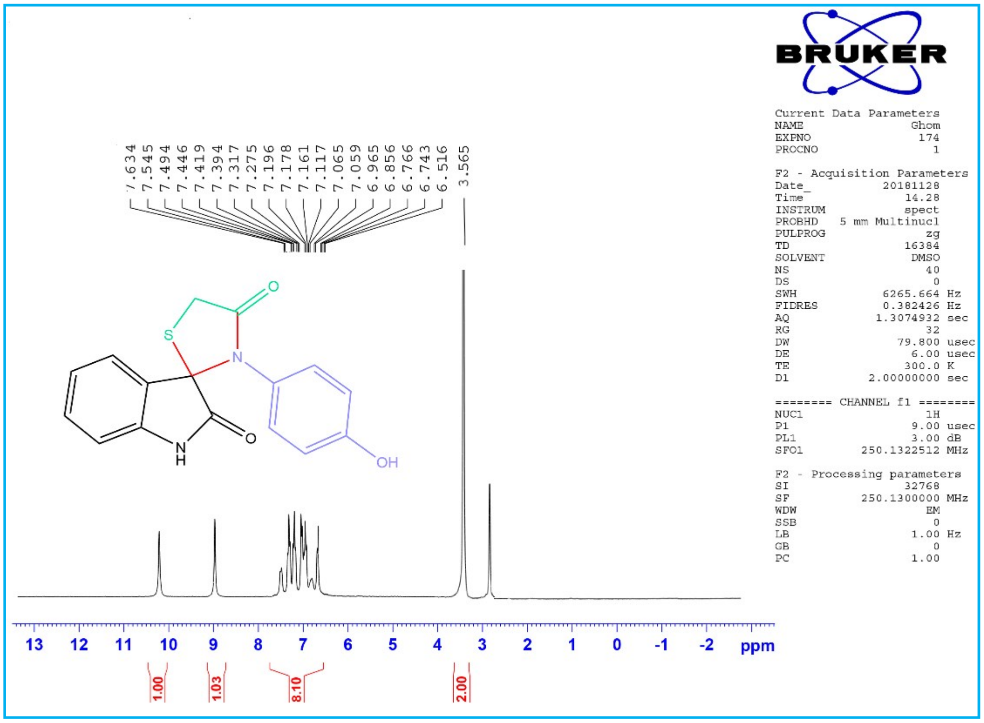

Figure S18: ^{1}H NMR spectrum of 3’-(4-Hydroxyphenyl)-spiro[3H-indole-3,2’-thiazolidine]-2,4'(1H)-dione (**4g**)

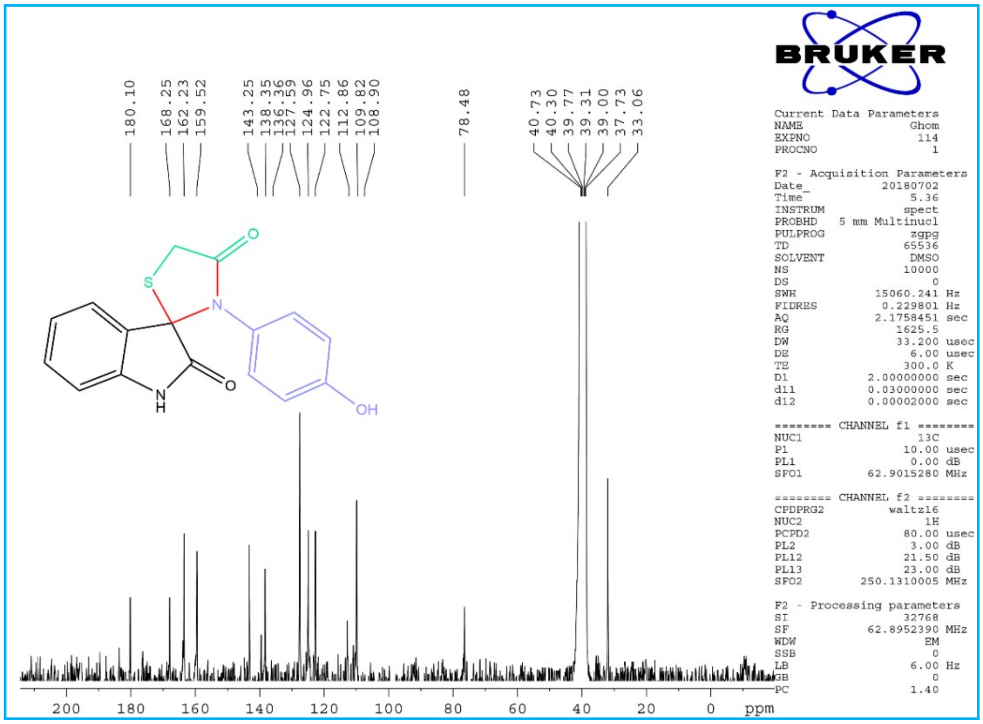

Figure S19: ^{13}C NMR spectrum of 3’-(4-Hydroxyphenyl)-spiro[3H-indole-3,2’-thiazolidine]-2,4'(1H)-dione (**4g**)

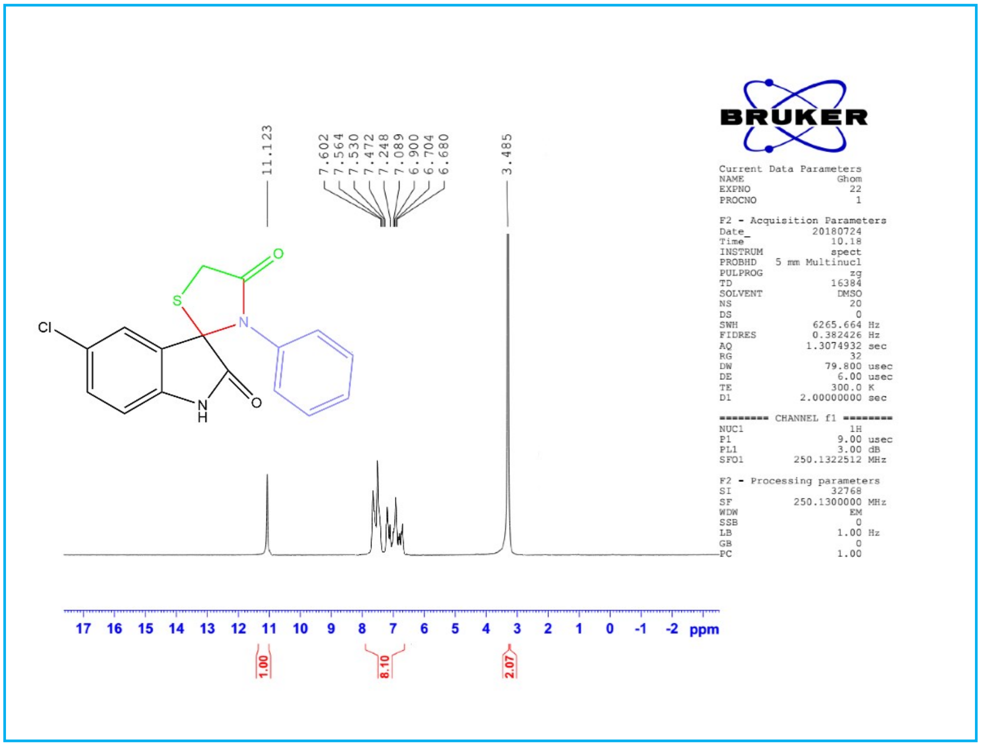

Figure S20: ^{1}H NMR spectrum of 5-Chloro-3'-phenylspiro[indoline-3,2'-thiazolidine]-2,4'-dione (**4h**)

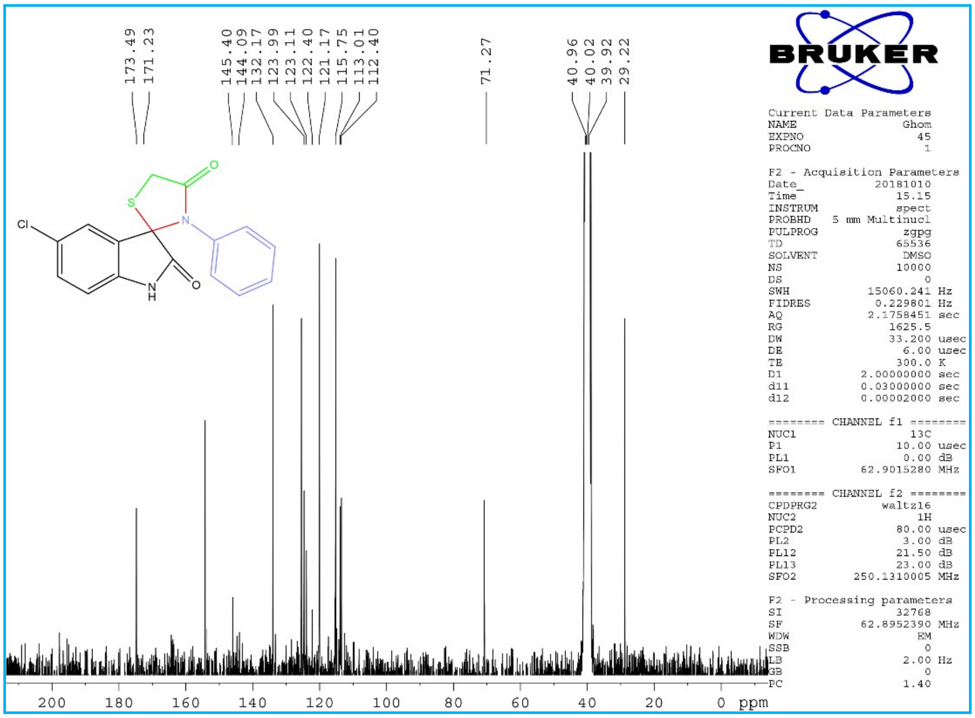

Figure S21: ^{13}C NMR spectrum of 5-Chloro-3'-phenylspiro[indoline-3,2'-thiazolidine]-2,4'-dione (**4h**)

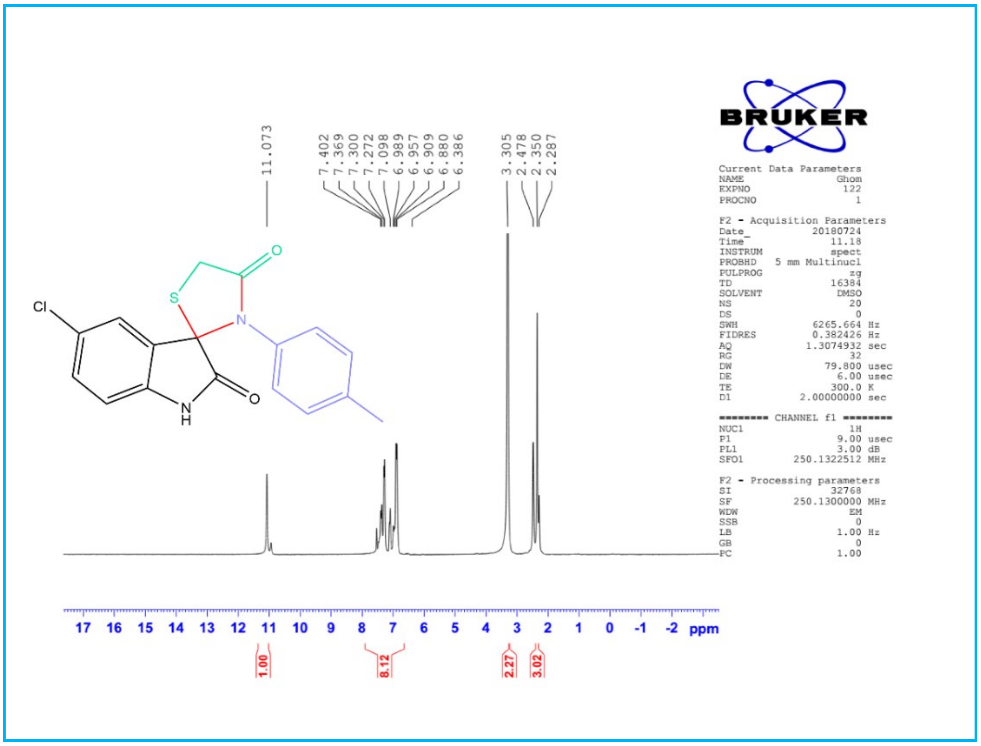

Figure S22: ^{1}H NMR spectrum of 3'-(4-Methyl)- 5-chlorospiro[3H-indole-3,2'-thiazolidine]-2,4'(1H)-dione (**4i**)

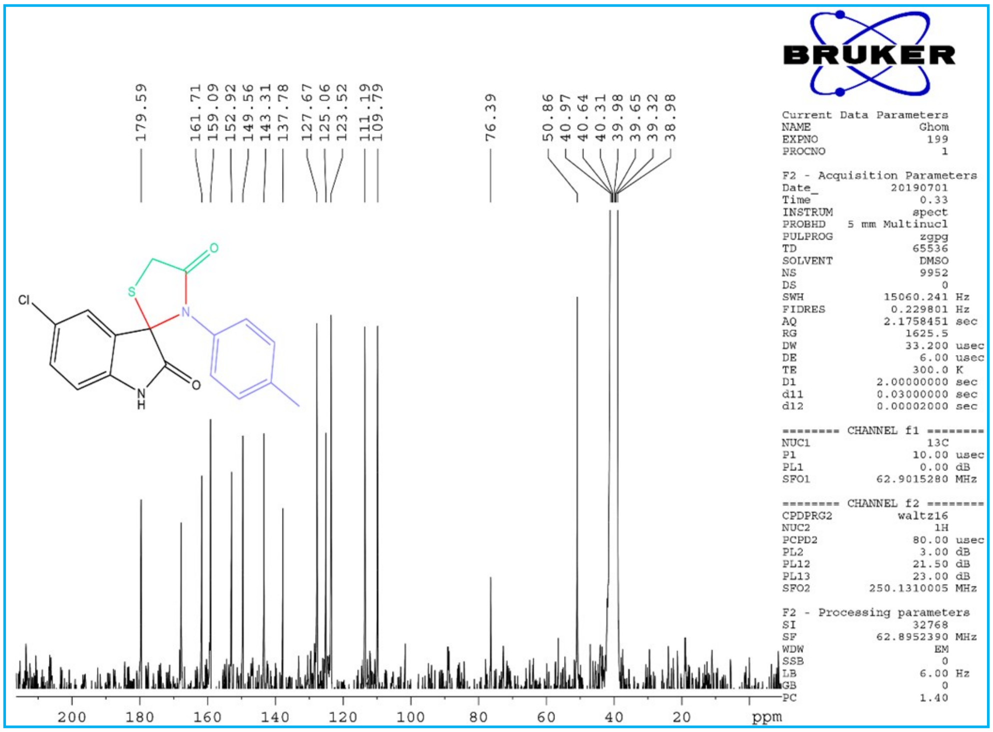

Figure S23: ^{13}C NMR spectrum of 3'-(4-Methyl)- 5-chlorospiro[3H-indole-3,2'-thiazolidine]-2,4'(1H)-dione (**4i**)

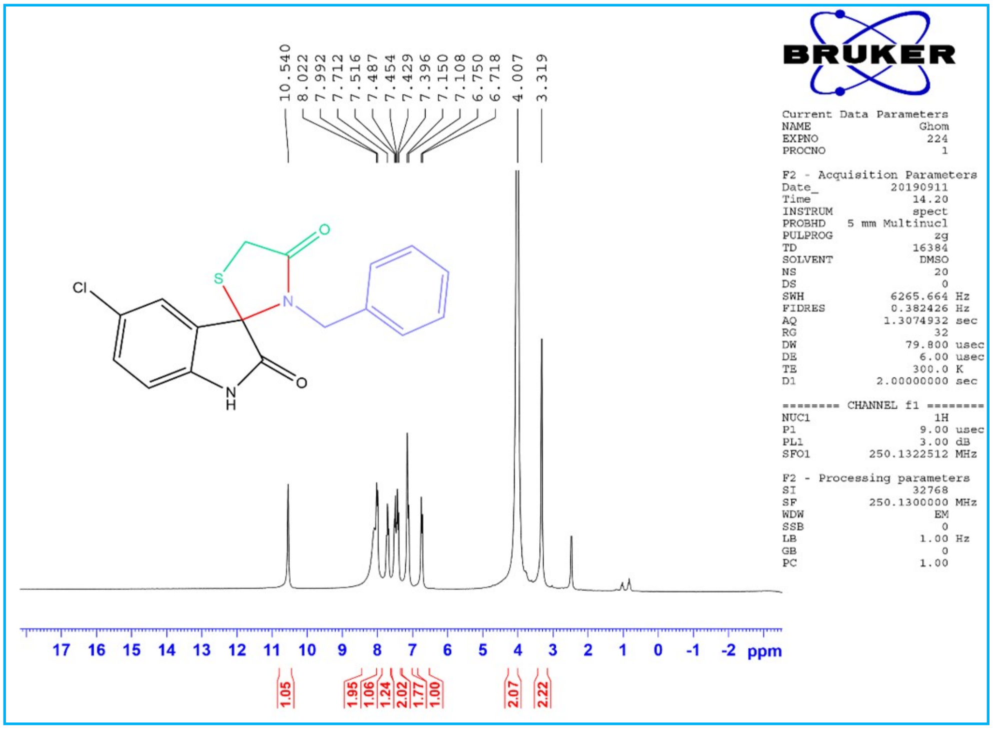

Figure S24: ^{1}H NMR spectrum of 3′-Benzyl-5-chlorospiro[indoline-3,2′-thiazolidine]-2,4′-dione (**4j**)

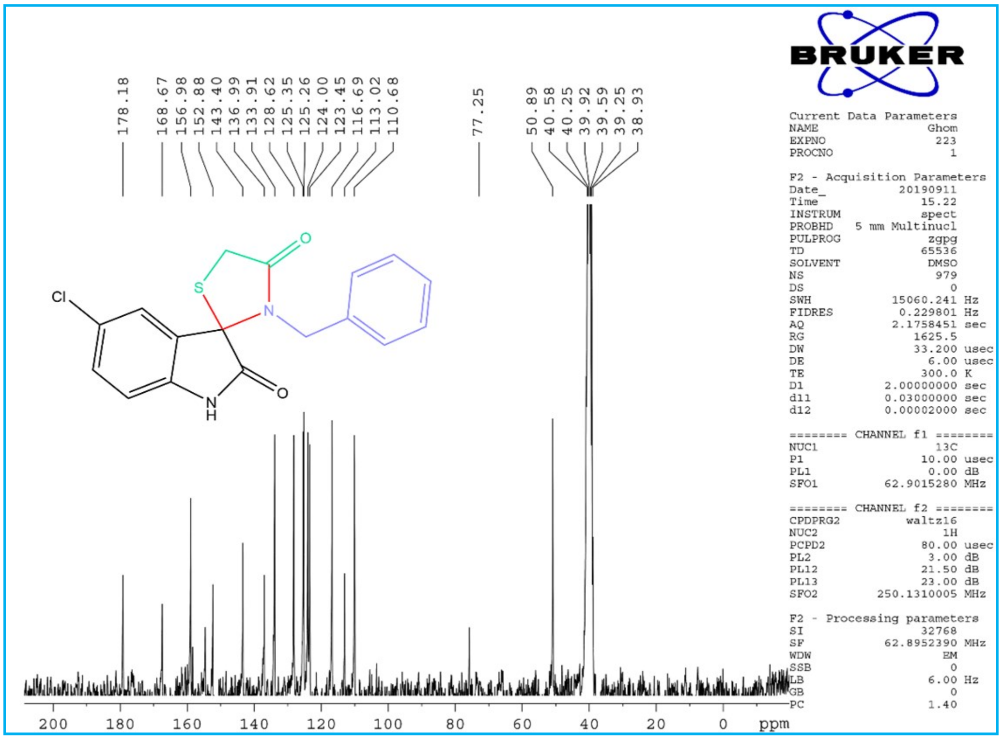

Figure S25: ^{13}C NMR spectrum of 3′-Benzyl-5-chlorospiro[indoline-3,2′-thiazolidine]-2,4′-dione (**4j**)

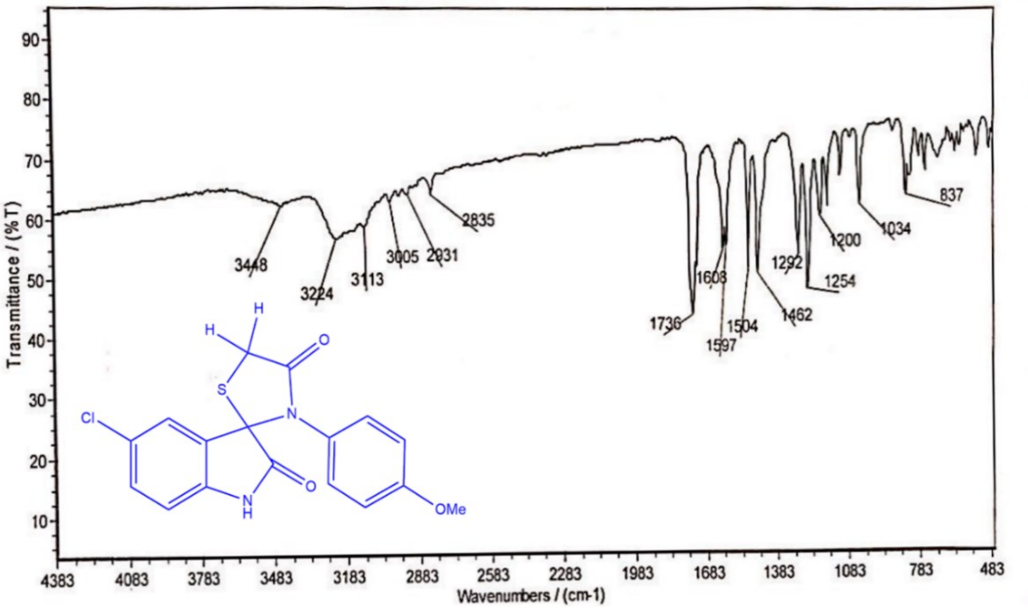

New compound, Figure S26: FT-IR (KBr) spectrum of 3’-(4-Methoxyphenyl)- 5-Chloro-3′-phenylspiro[3H-indole-3,2’-thiazolidine]-2,4'(1H)-dione (**4k**)

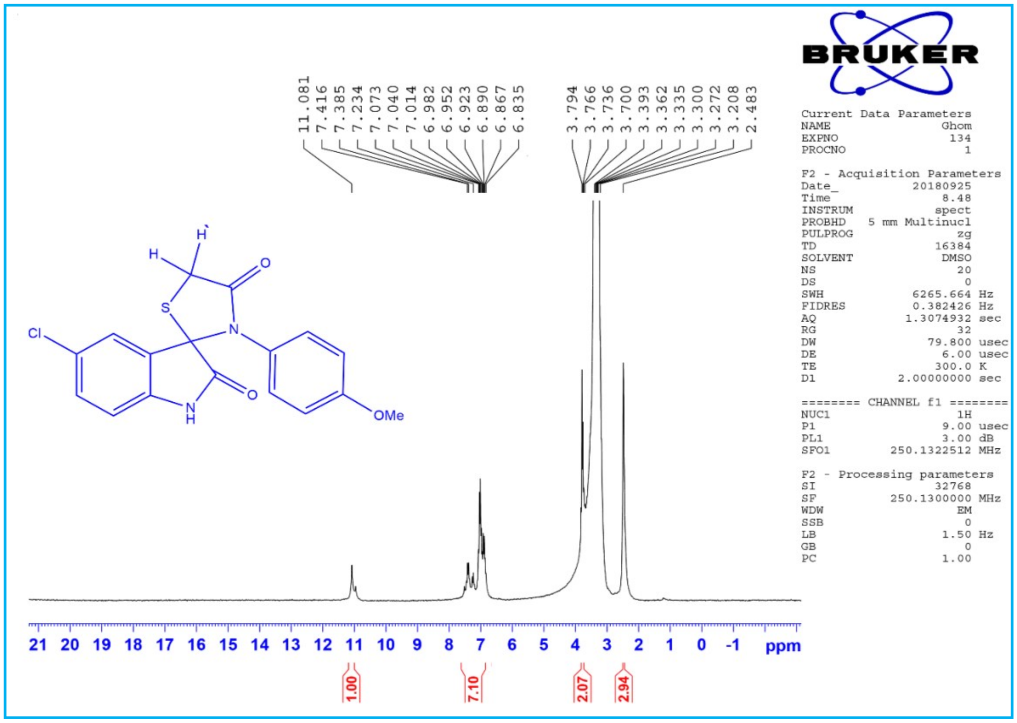

New compound, Figure S27: ^{1}H NMR spectrum of 3’-(4-Methoxyphenyl)- 5-Chloro-3′-phenylspiro[3H-indole-3,2’-thiazolidine]-2,4'(1H)-dione (**4k**)

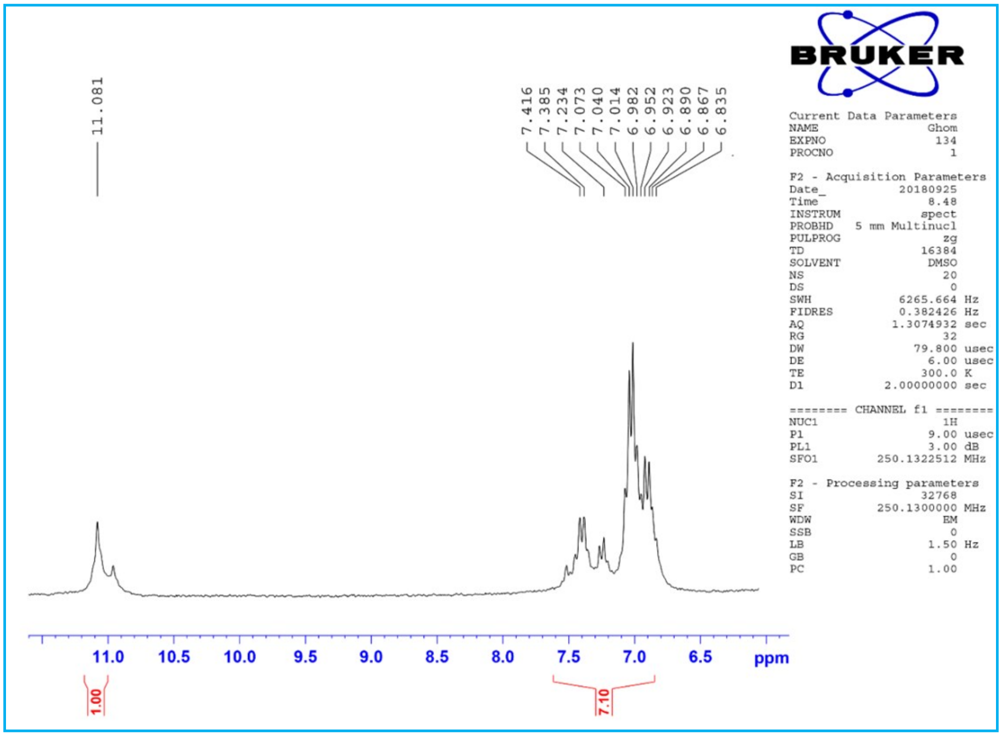

New compound, Figure S28: ^{1}H NMR spectrum of 3’-(4-Methoxyphenyl)- 5-Chloro-3′-phenylspiro[3H-indole-3,2’-thiazolidine]-2,4'(1H)-dione (**4k**)

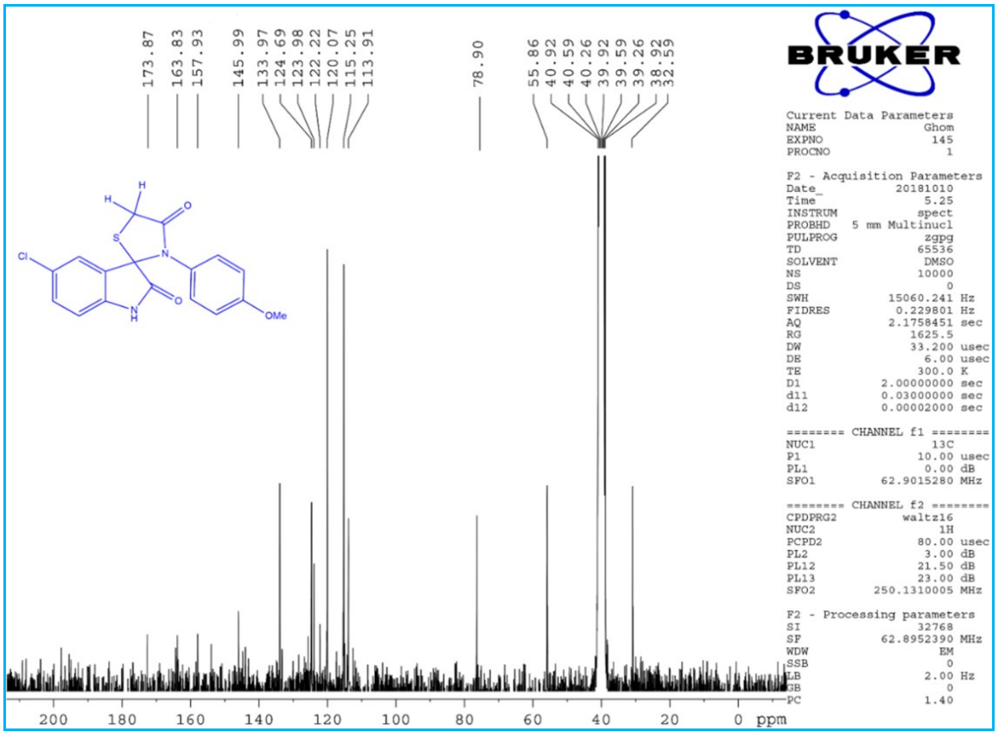

New compound, Figure S29: ^{13}C NMR spectrum of 3’-(4-Methoxyphenyl)- 5-Chloro-3′-phenylspiro[3H-indole-3,2’-thiazolidine]-2,4'(1H)-dione (**4k**)

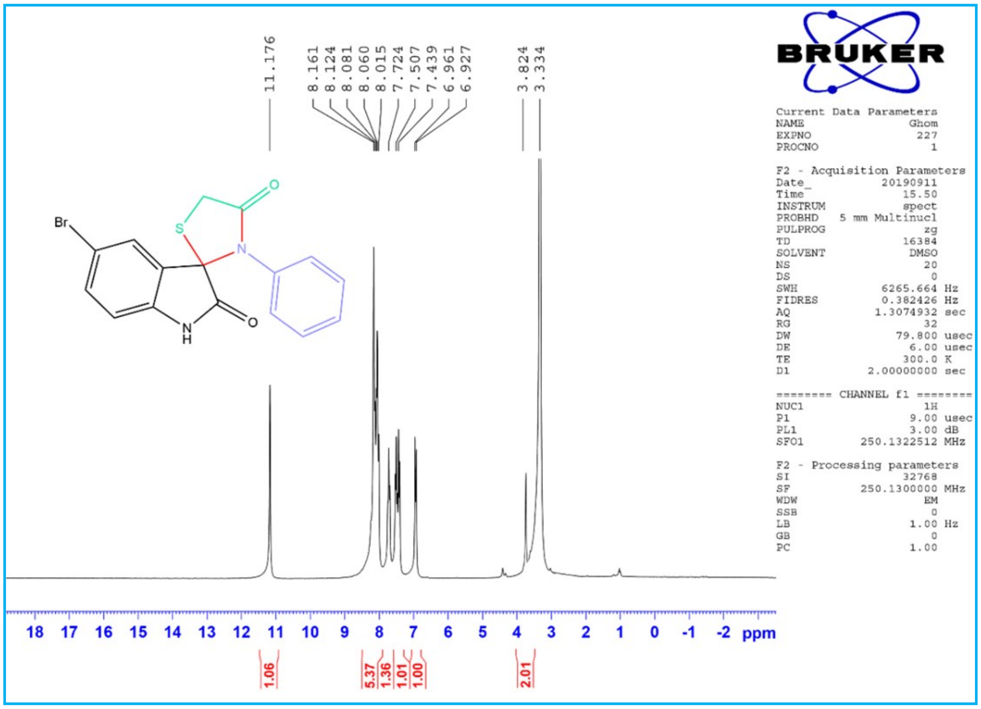

Figure S30: ^{1}H NMR spectrum of 5-Bromo-3′-phenylspiro[indoline-3,2′-thiazolidine]-2,4′-dione (**4l**)

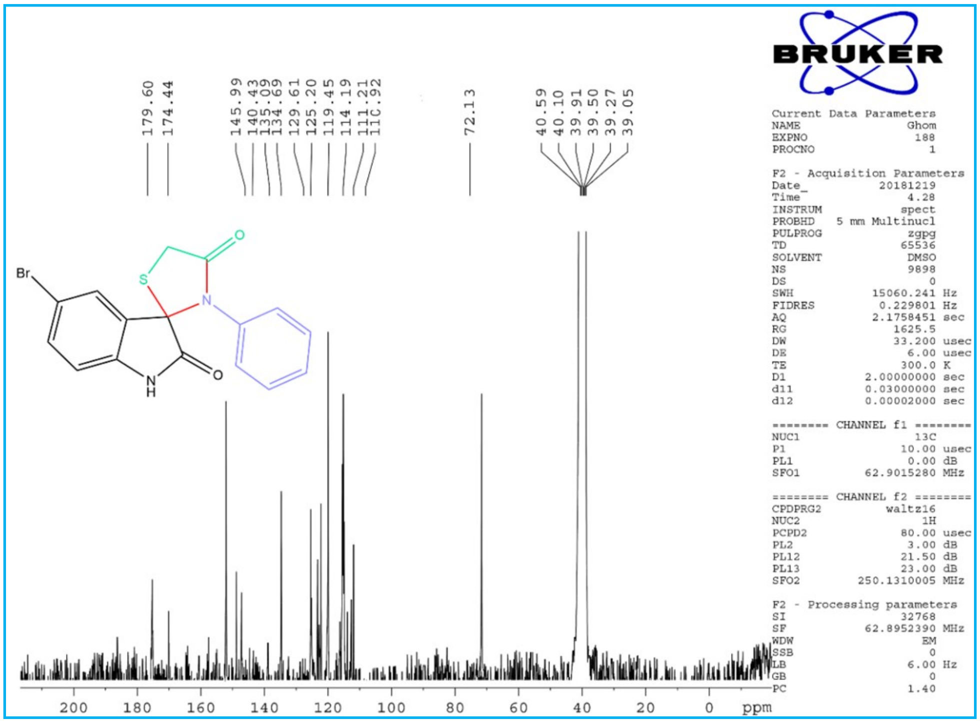

Figure S31: ^{13}C NMR spectrum of 5-Bromo-3′-phenylspiro[indoline-3,2′-thiazolidine]-2,4′-dione (**4l**)

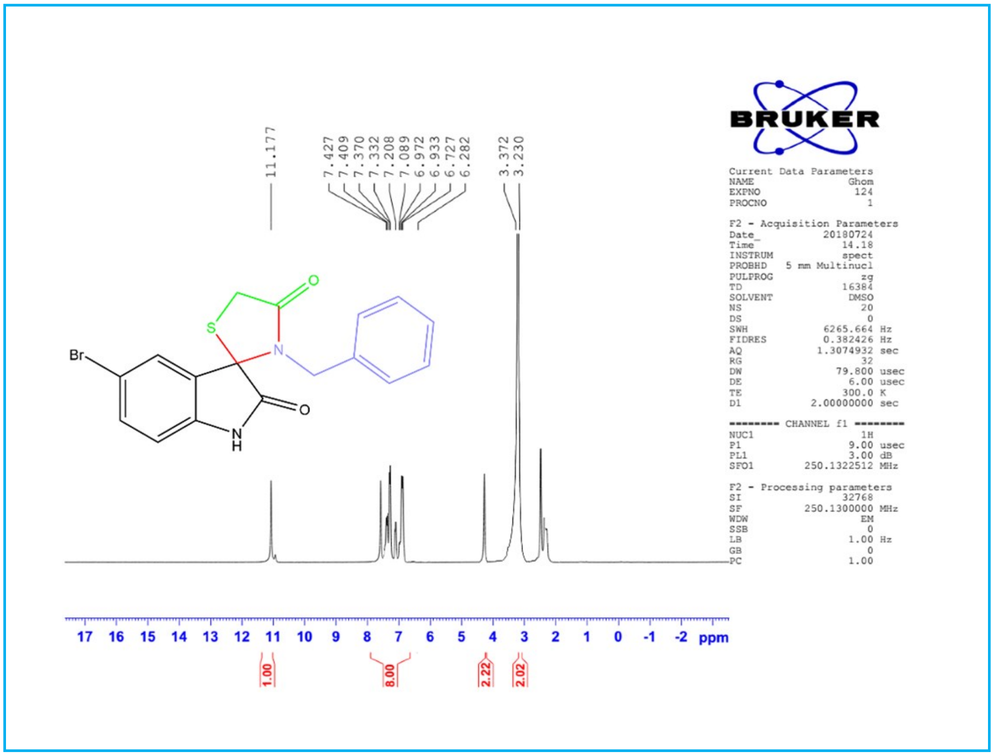

Figure S32: ^{1}H NMR spectrum of 3'-Benzyl-5-bromospiro[indoline-3,2'-thiazolidine]-2,4'-dione (**4m**)

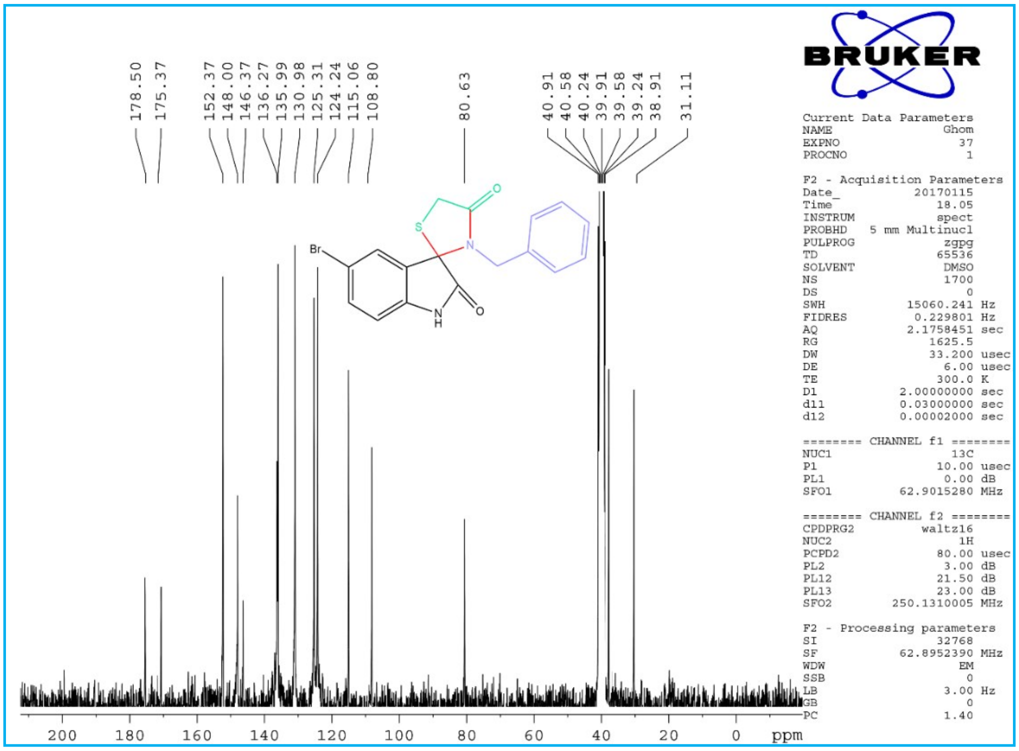

Figure S33: ^{13}C NMR spectrum of 3′-Benzyl-5-bromospiro[indoline-3,2′-thiazolidine]-2,4′-dione (**4m**)

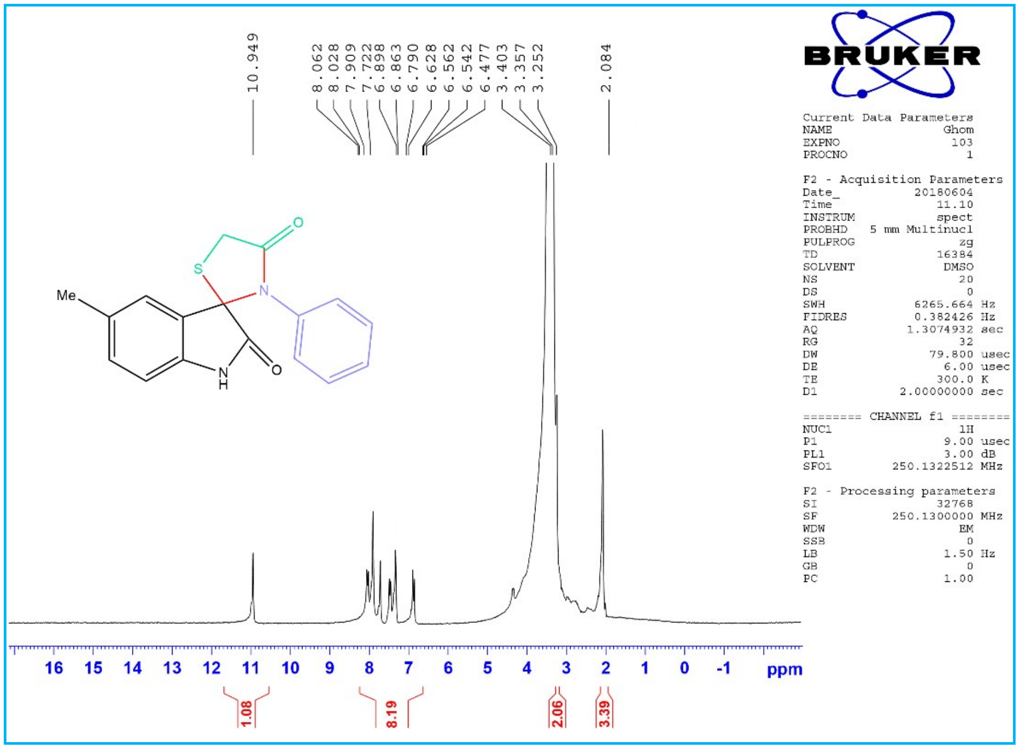

Figure S34: ^{1}H NMR spectrum of 5-Methyl-3′-phenylspiro[indoline-3,2′-thiazolidine]-2,4′-dione (**4n**)

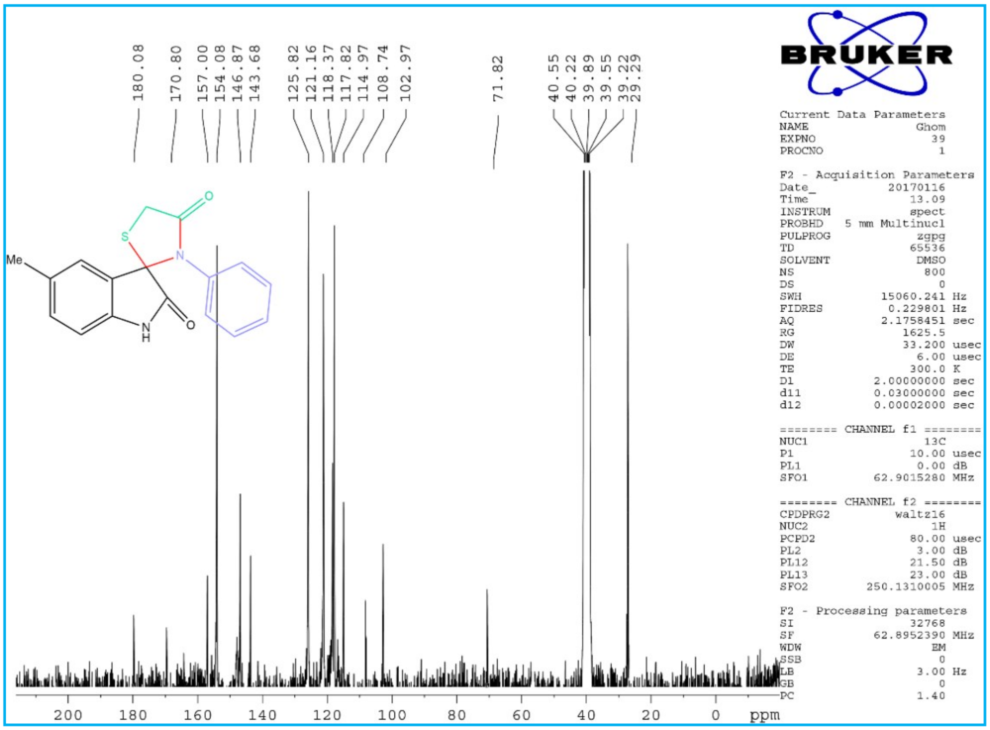

Figure S35: ^{13}C NMR spectrum of 5-Methyl-3′-phenylspiro[indoline-3,2′-thiazolidine]-2,4′-dione (**4n**)

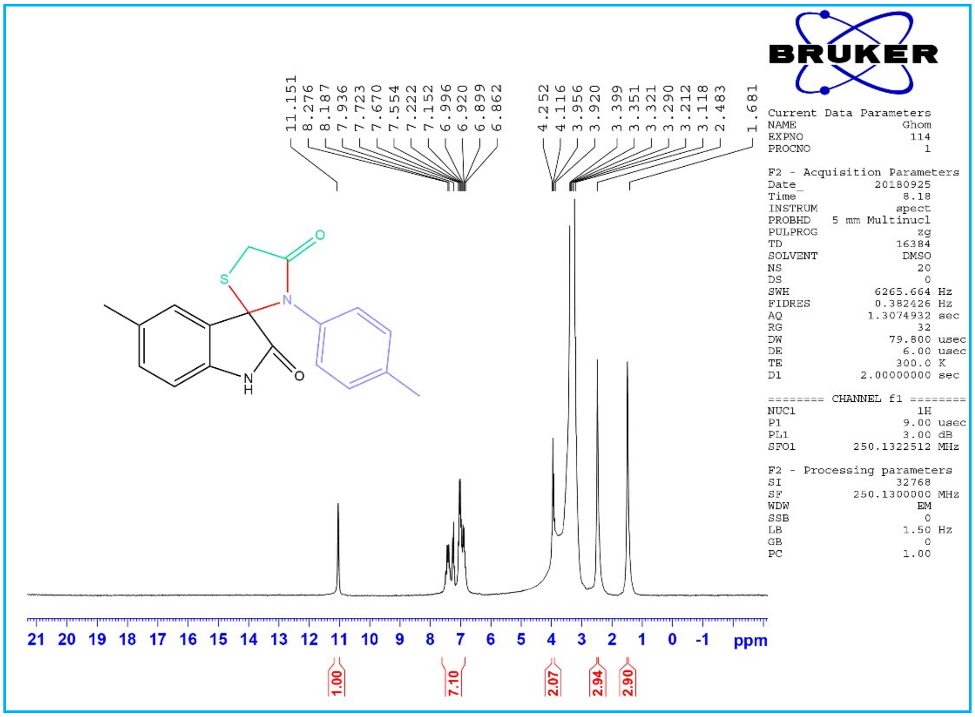

Figure S36: ^{1}H NMR spectrum of 3'-(4-Methyl)- 5-methylspiro[3H-indole-3,2'-thiazolidine]-2,4'(1H)-dione (**4o**)

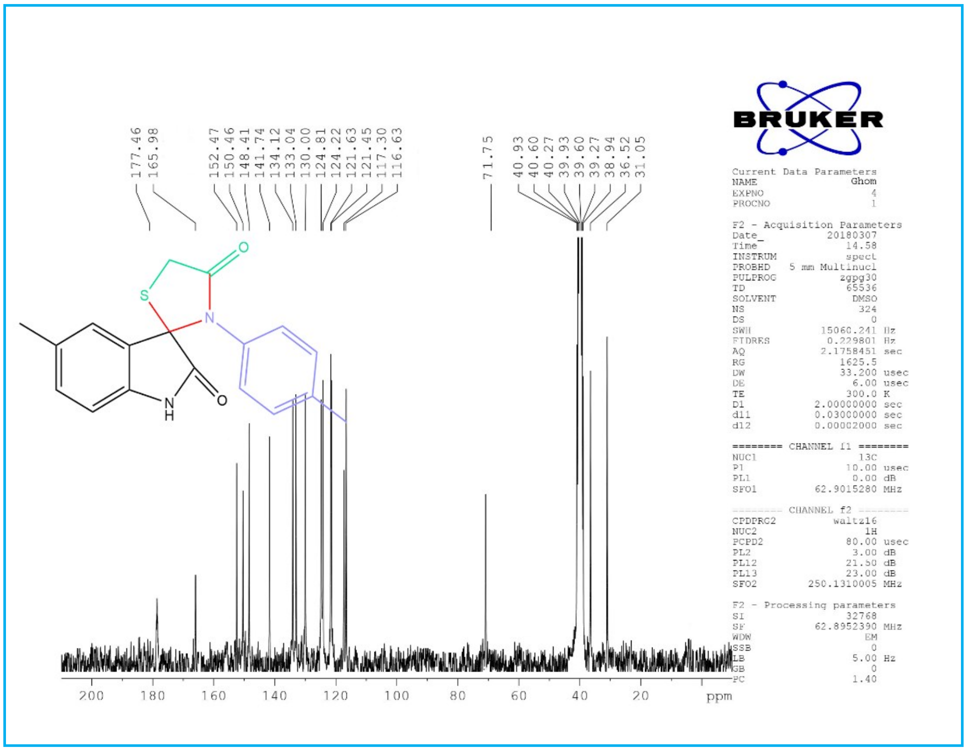

Figure S37: ^{13}C NMR spectrum of 3'-(4-Methyl)- 5-methylspiro[3H-indole-3,2'-thiazolidine]-2,4'(1H)-dione (**4o**)

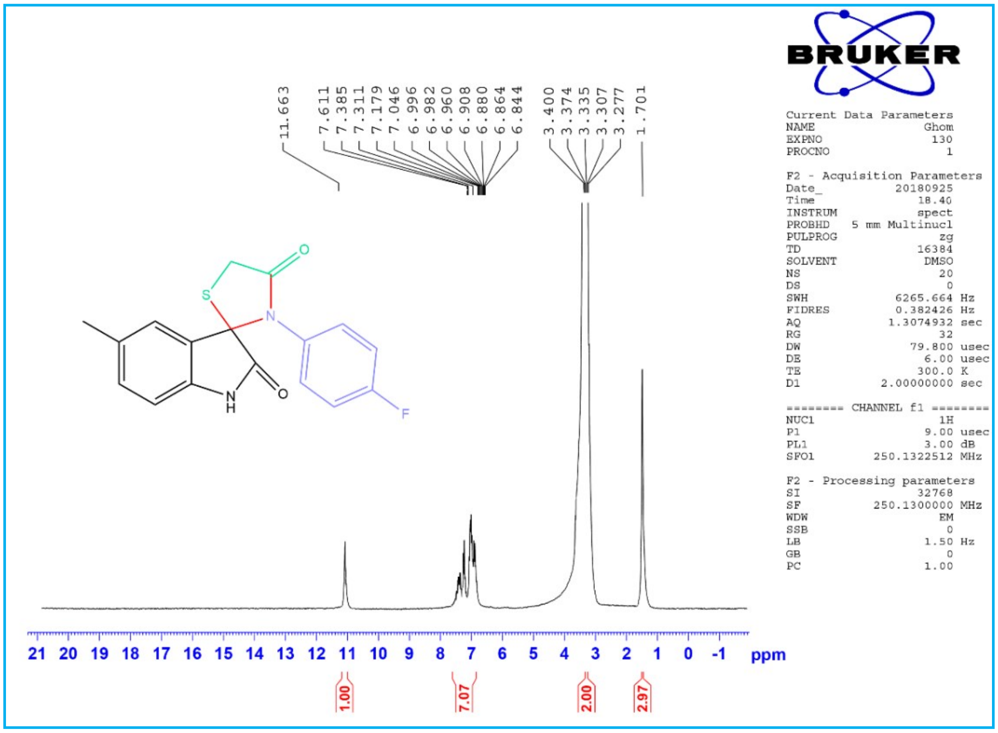

Figure S38: ^{1}H NMR spectrum of 3’-(4-Fluorophenyl)- 5-methylspiro[3H-indole-3,2’-thiazolidine]-2,4'(1H)-dione (**4p**)

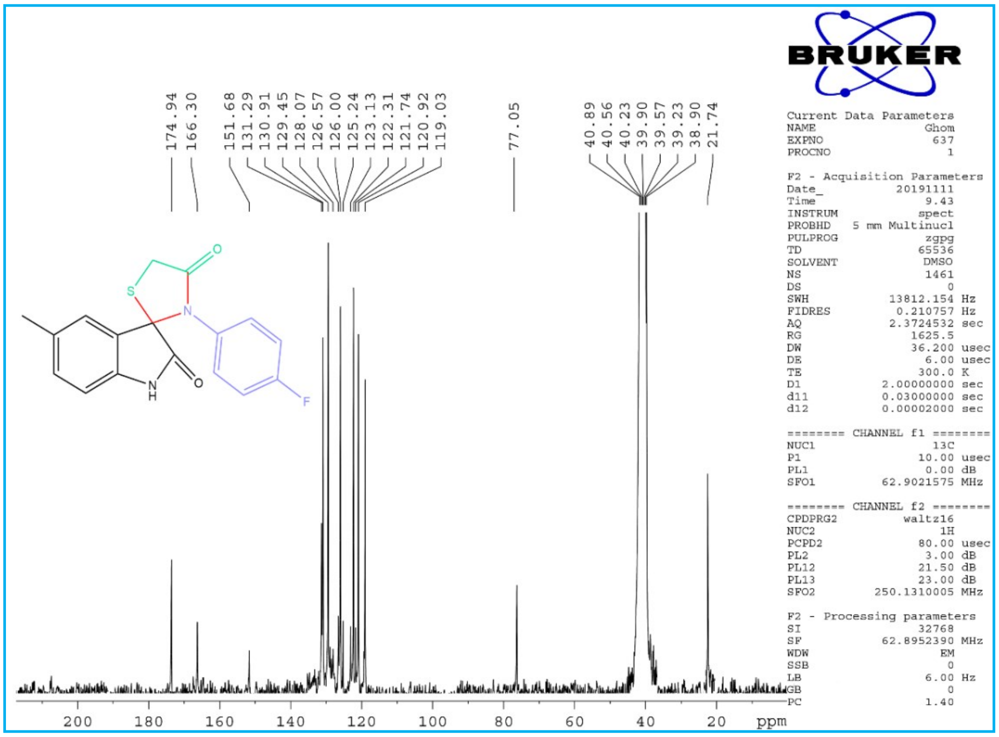

Figure S39: ^{13}C NMR spectrum of 3’-(4-Fluorophenyl)- 5-methylspiro[3H-indole-3,2’-thiazolidine]-2,4'(1H)-dione (**4p**)

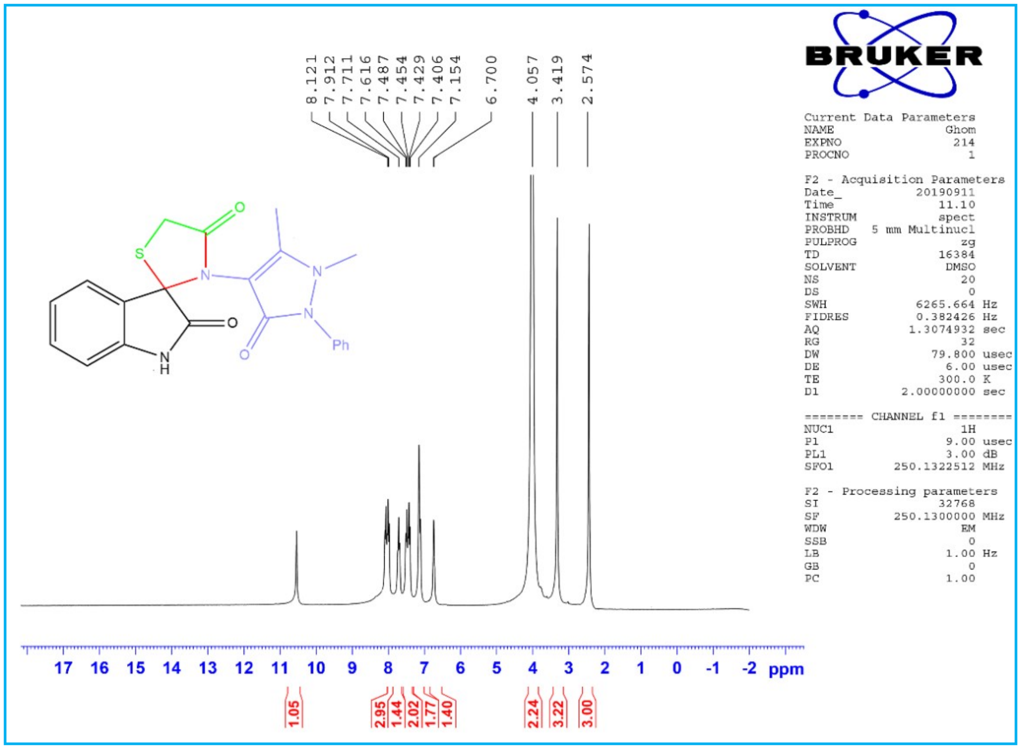

Figure S40: ^{1}H NMR spectrum of 3`-(2,3-dihydro-1,5-dimethyl-3-oxo-2-phenyl-1Hpyrazol-4-yl)-spiro[3H-indole-3,2`-thiazolidine]-2,4`(1H) dione (**4q**)

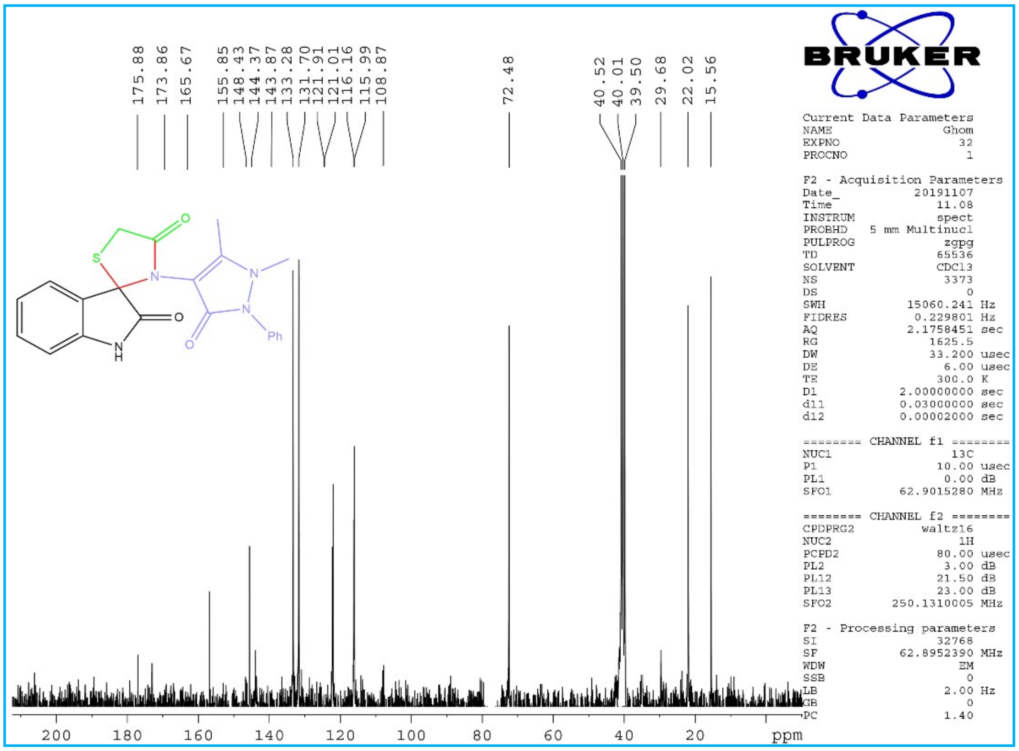

Figure S41: ^{13}C NMR spectrum of 3`-(2,3-dihydro-1,5-dimethyl-3-oxo-2-phenyl-1Hpyrazol-4-yl)-spiro[3H-indole-3,2`-thiazolidine]-2,4`(1H) dione (**4q**)

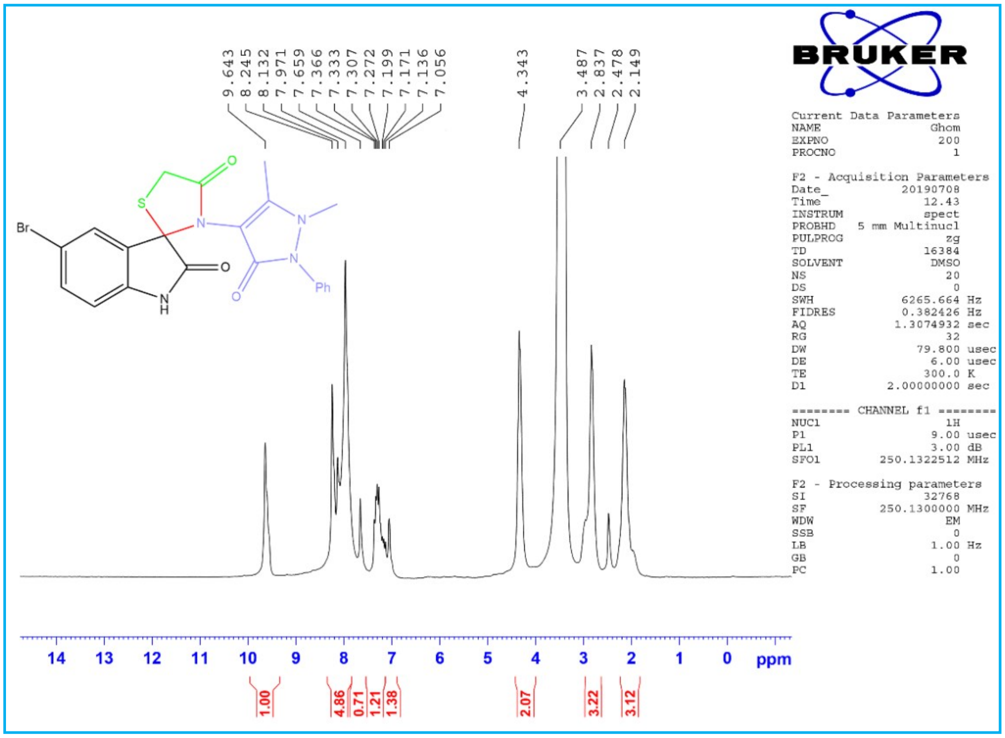

Figure S42: ^{1}H NMR spectrum of 5-Bromo-3`-(2,3-dihydro-1,5-dimethyl-3-oxo-2-phenyl-1Hpyrazol-4-yl)-spiro[3H-indole-3,2`-thiazolidine]-2,4`(1H) dione (**4r**)

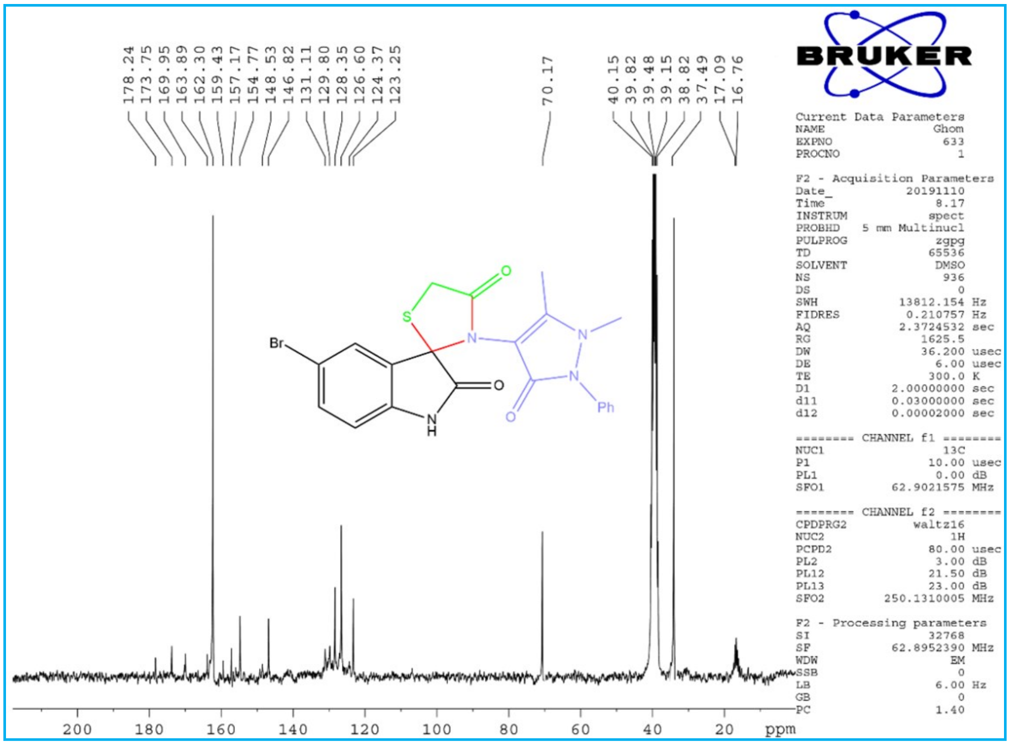

Figure S43: ^{13}C NMR spectrum of 5-Bromo-3`-(2,3-dihydro-1,5-dimethyl-3-oxo-2-phenyl-1Hpyrazol-4-yl)-spiro[3H-indole-3,2`-thiazolidine]-2,4`(1H) dione (**4r**)

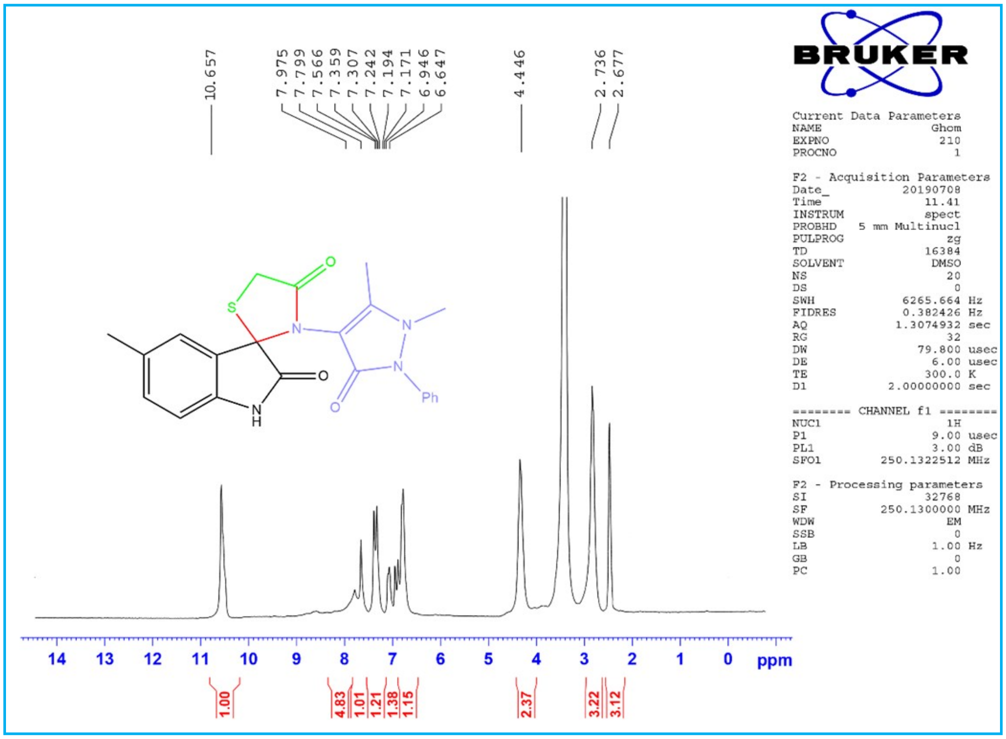

Figure S44: 1H NMR spectrum of 5-Methyl-3`-(2,3-dihydro-1,5-dimethyl-3-oxo-2-phenyl-1Hpyrazol-4-yl)-spiro[3H-indole-3,2`-thiazolidine]-2,4`(1H) dione (**4s**)

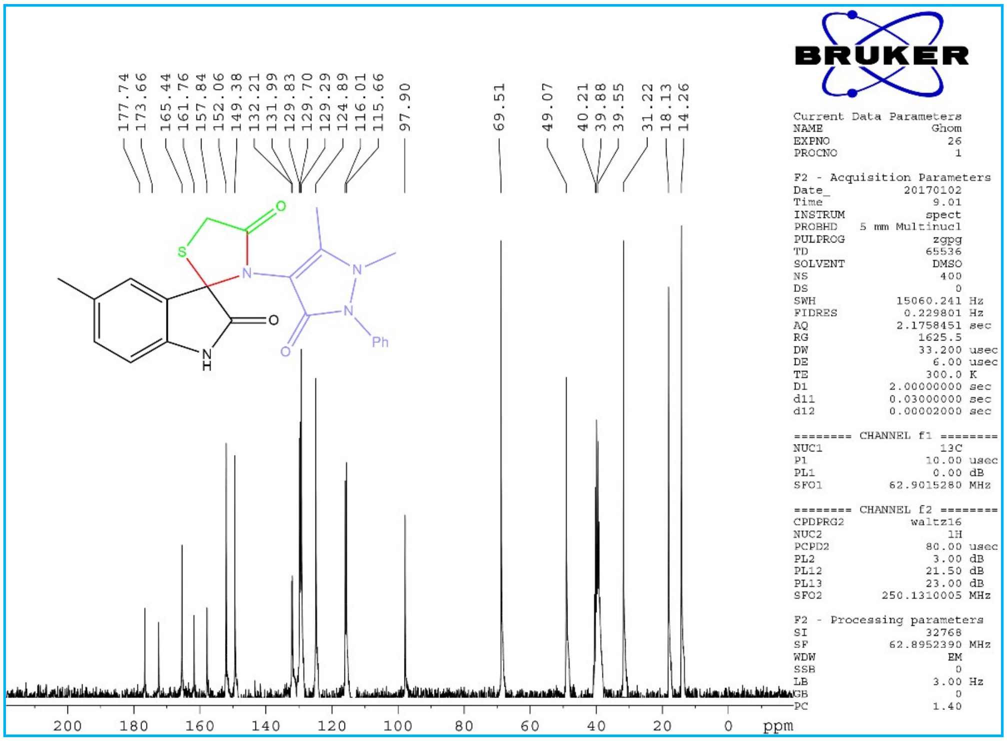

Figure S45: ^{13}C NMR spectrum of 5-Methyl-3`-(2,3-dihydro-1,5-dimethyl-3-oxo-2-phenyl-1Hpyrazol-4-yl)-spiro[3H-indole-3,2`-thiazolidine]-2,4`(1H) dione (**4s**)

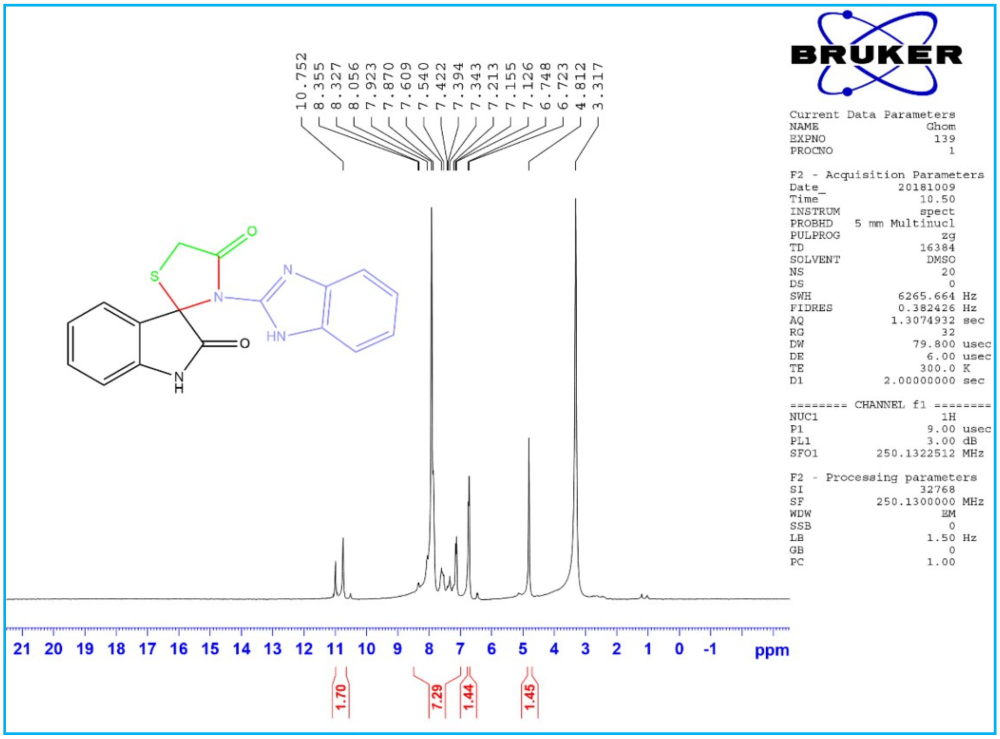

Figure S46: ^{1}H NMR spectrum of 3`-(1H-benzimidazol-2-yl)-spiro-[3H-indole-3,2`thiazolidine]-2,4`(1H)-dione (**4t**)

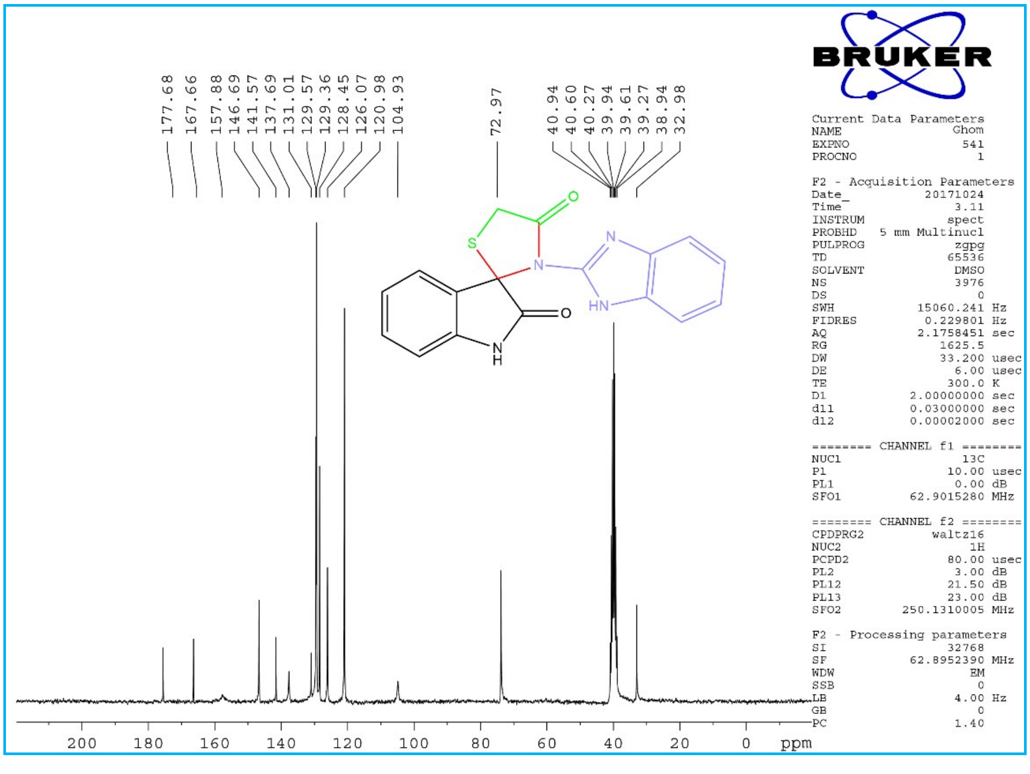

Figure S47: ^{13}C NMR spectrum of 3`-(1H-benzimidazol-2-yl)-spiro-[3H-indole-3,2`thiazolidine]-2,4`(1H)-dione (**4t**)

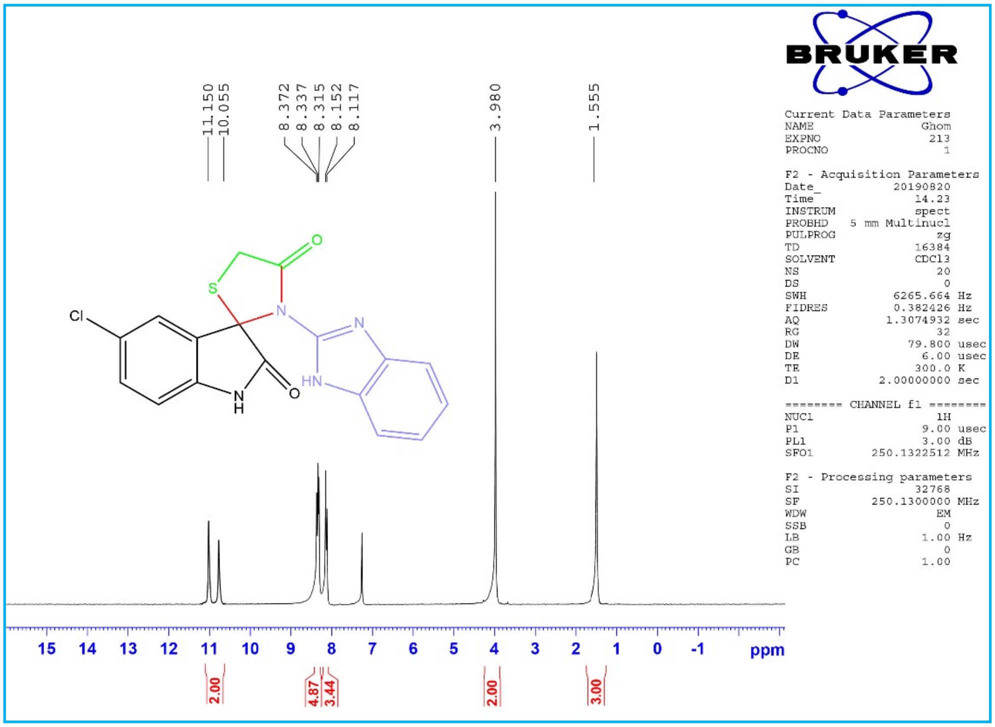

Figure S48: ^{1}H NMR spectrum of 5-Chloro-3`-(1H-benzimidazol-2-yl)-spiro-[3H-indole 3,2`thiazolidine]-2,4`(1H)-dione (**4u**)

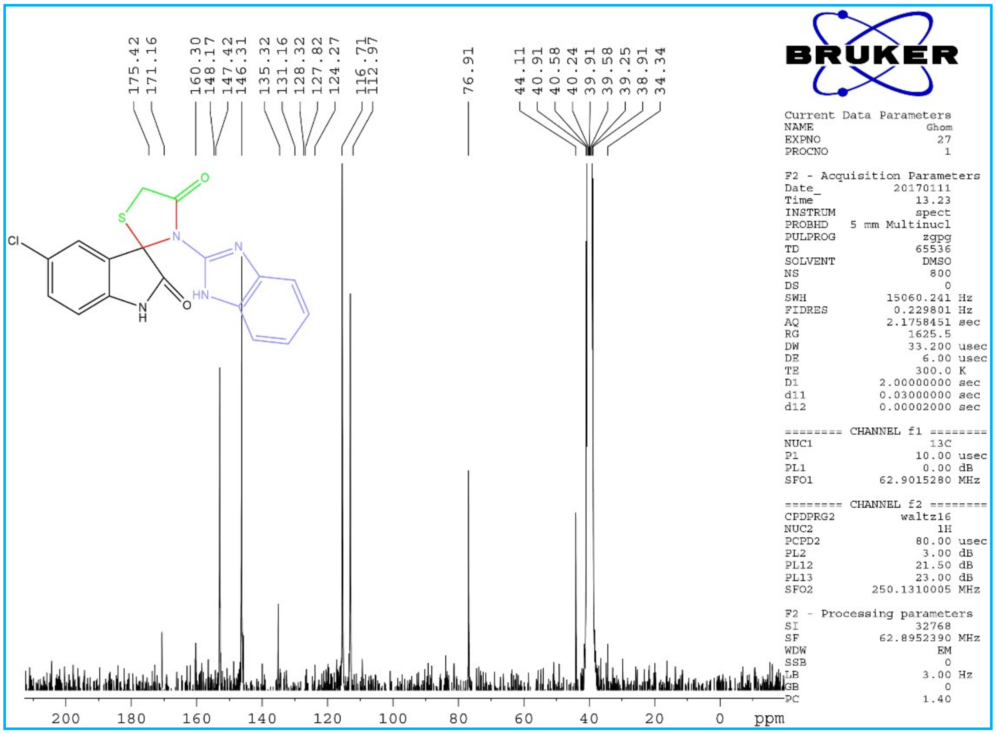

Figure S49: ^{13}C NMR spectrum of 5-Chloro-3`-(1H-benzimidazol-2-yl)-spiro-[3H-indole 3,2`thiazolidine]-2,4`(1H)-dione (**4u**)

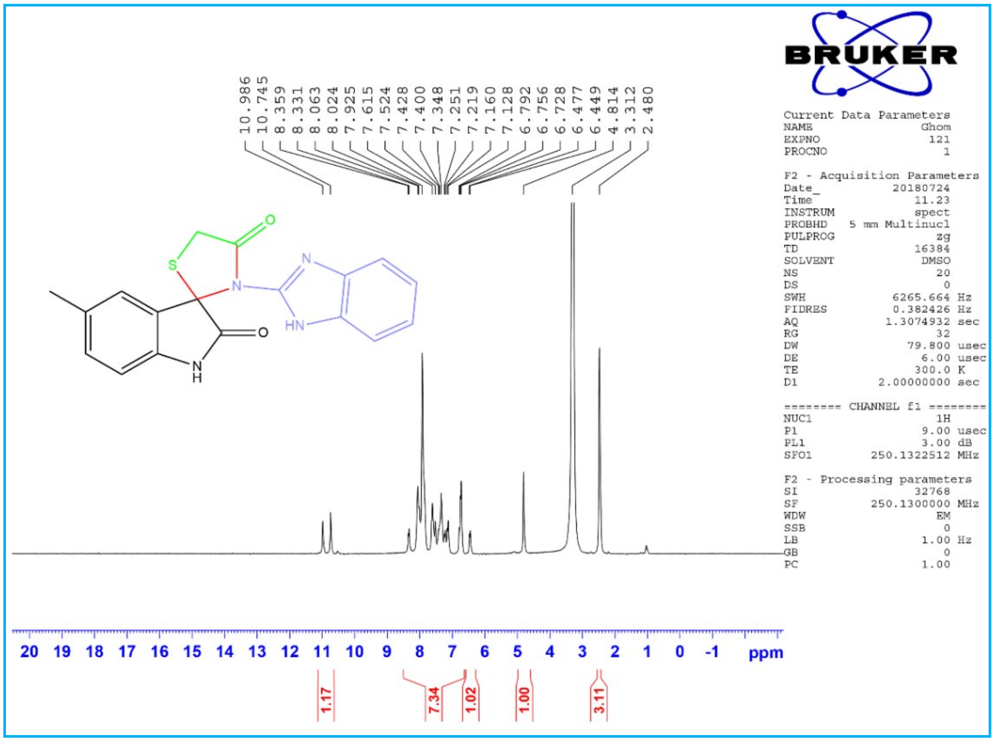

Figure S50: ^{1}H NMR spectrum of 5-Methyl-3`-(1H-benzimidazol-2-yl)-spiro-[3H-indole3,2`thiazolidine]-2,4`(1H)-dione (**4v**)

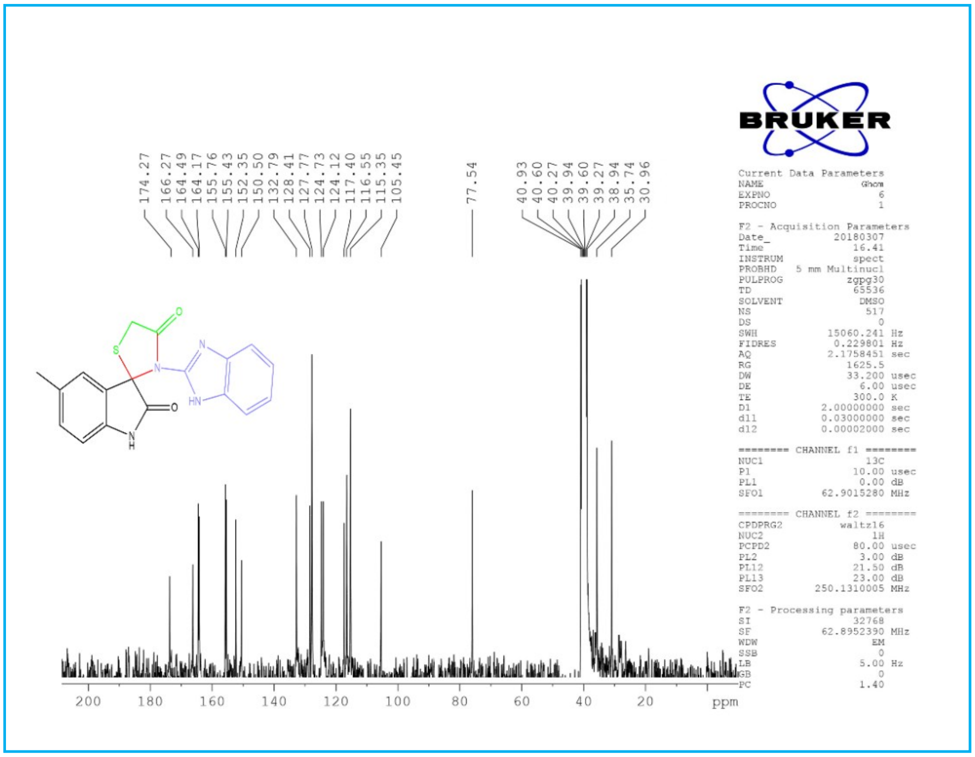

Figure S51: ^{13}C NMR spectrum of 5-Methyl-3`-(1H-benzimidazol-2-yl)-spiro-[3H-indole3,2`thiazolidine]-2,4`(1H)-dione (**4v**)

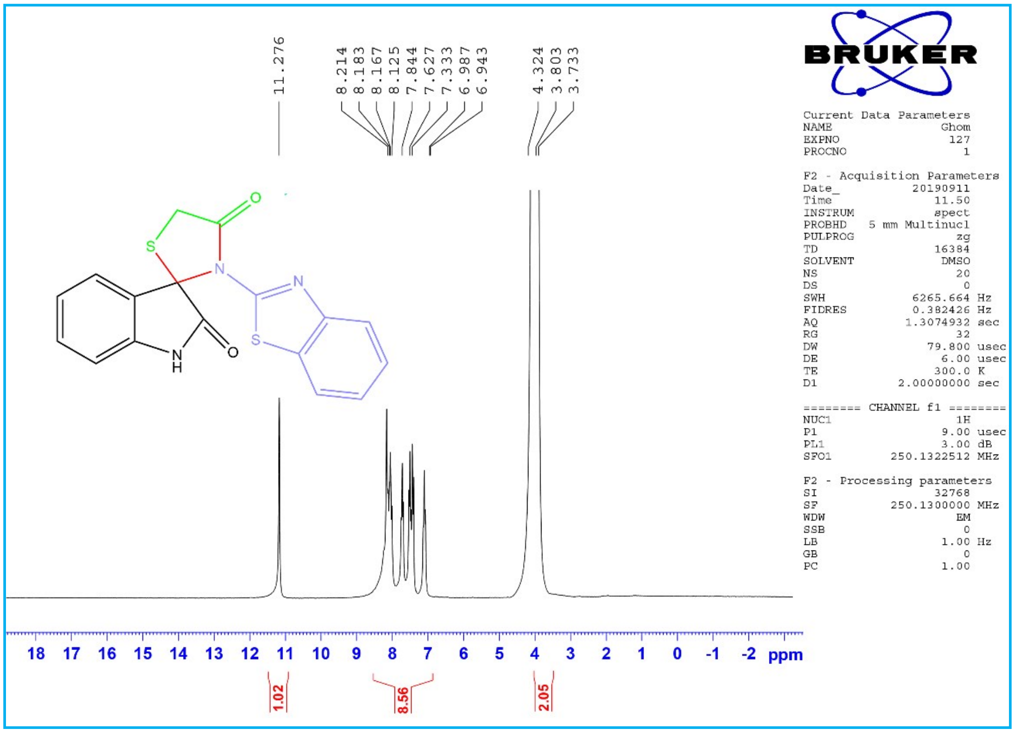

Figure S52: ^{1}H NMR spectrum of 3`-(1,3-Benzothiazol-2-yl)-spiro-[3H-indole-3,2`thiazolidine]2,4`(1H)-dione (**4w**)

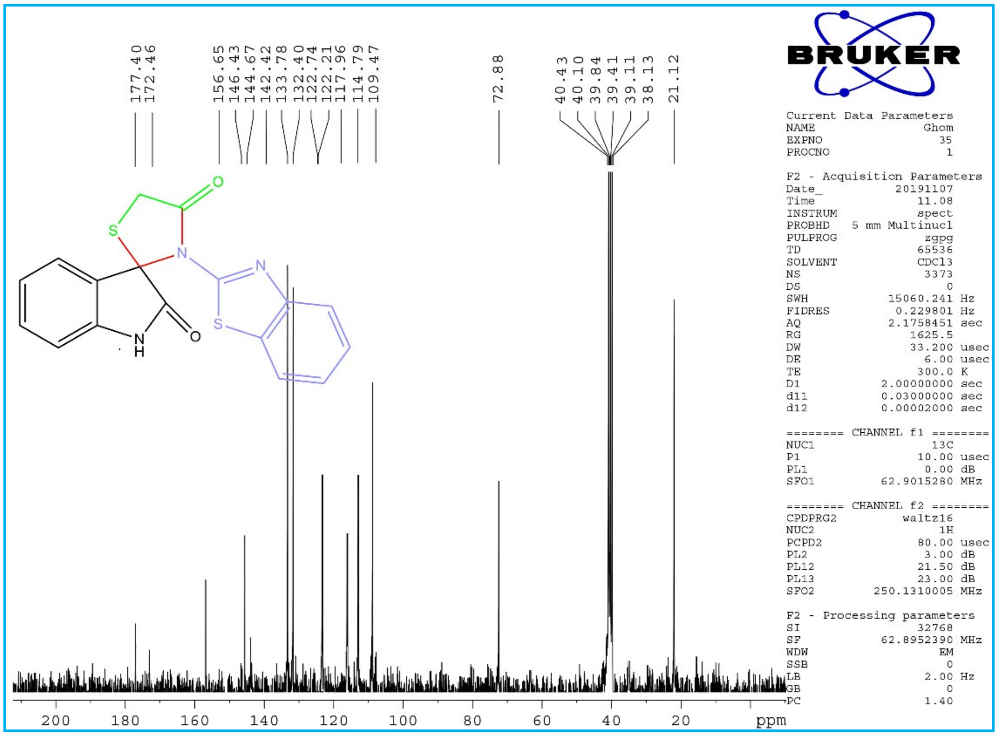

Figure S53: ^{13}C NMR spectrum of 3`-(1,3-Benzothiazol-2-yl)-spiro-[3H-indole-3,2`thiazolidine]2,4`(1H)-dione (**4w**)

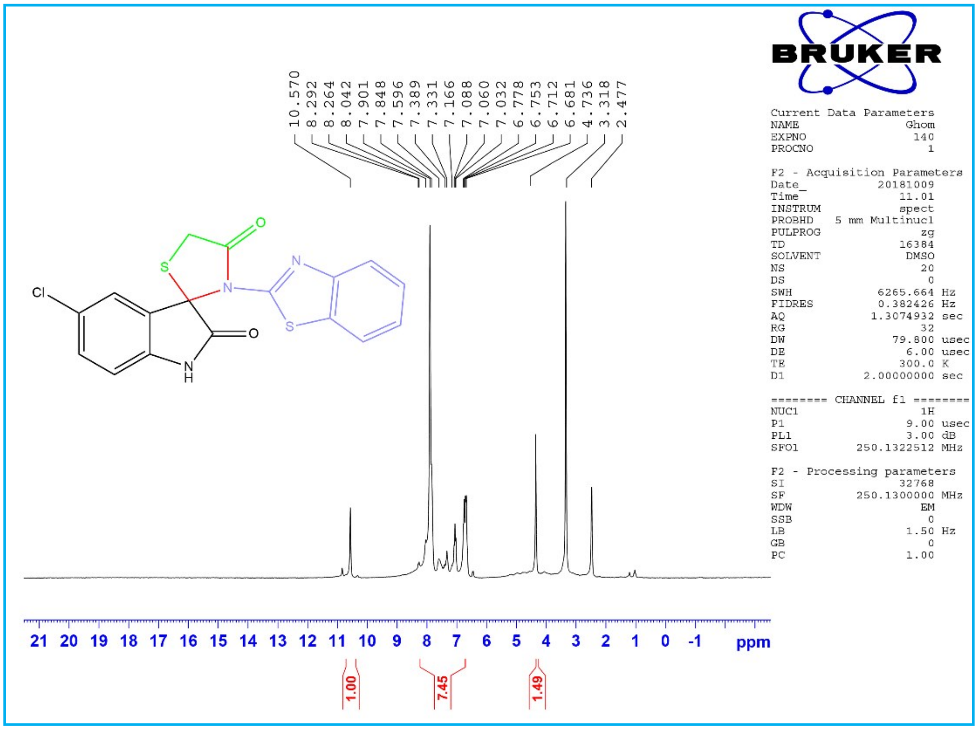

Figure S54: ^{1}H NMR spectrum of 5-Chloro-3`-(1,3-benzothiazol-2-yl)-spiro-[3H-indole-3,2`thiazolidine]-2,4`(1H)-dione (**4x**)

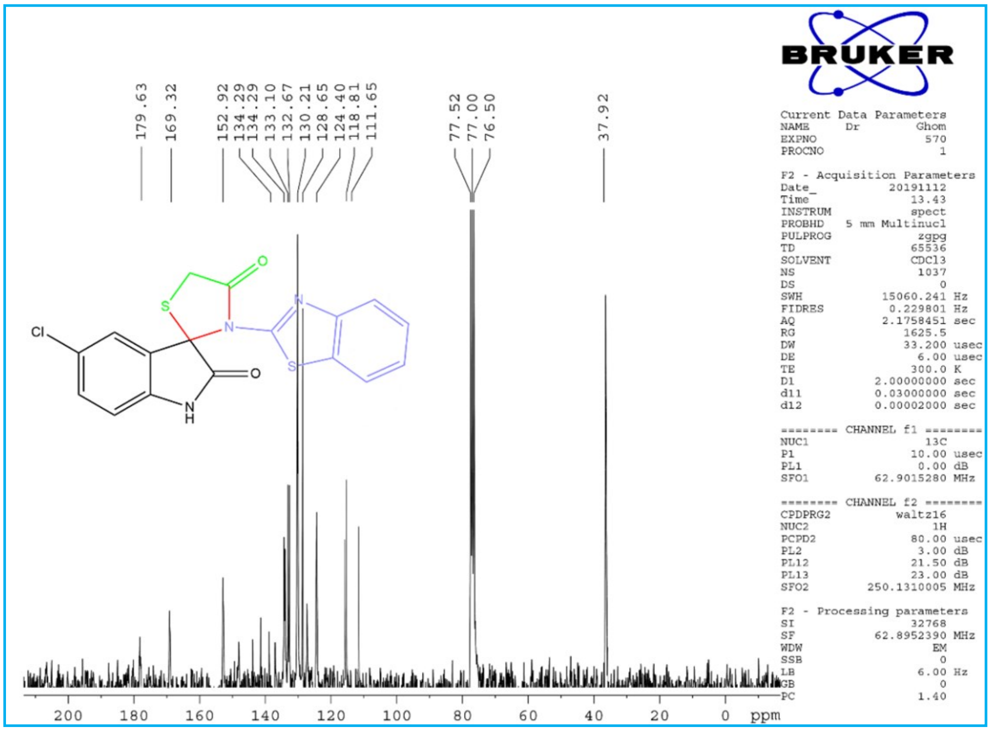

Figure S55: ^{13}C NMR spectrum of 5-Chloro-3`-(1,3-benzothiazol-2-yl)-spiro-[3H-indole-3,2`thiazolidine]-2,4`(1H)-dione (**4x**)

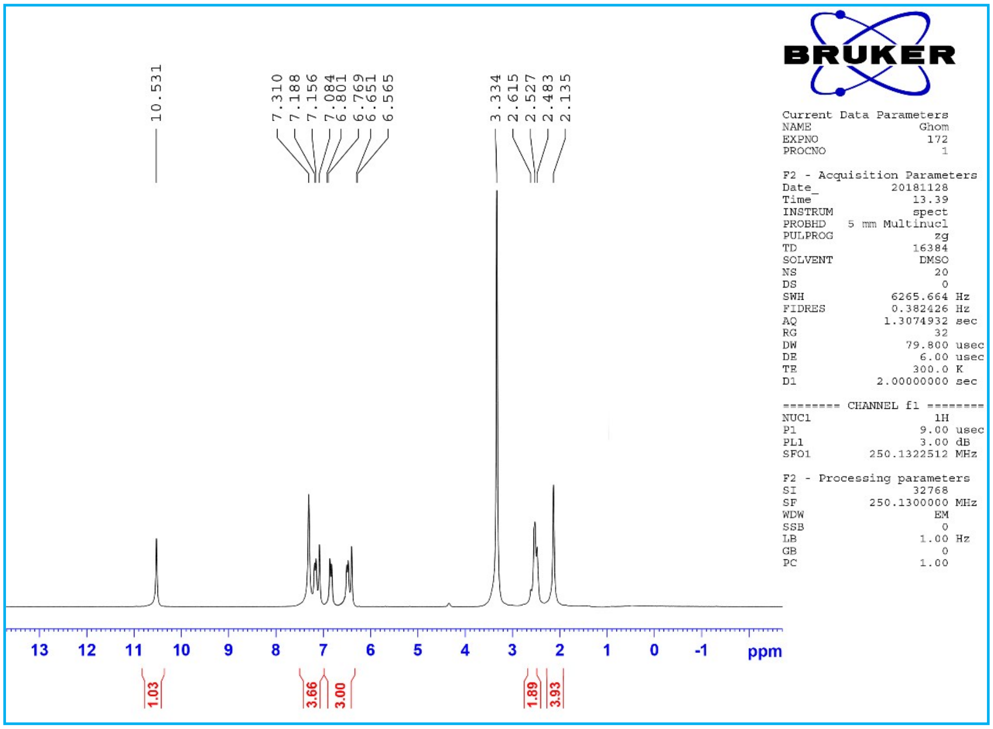

Figure S56: ^{1}H NMR spectrum of 5-Methyl-3`-(1,3-benzothiazol-2-yl)-spiro-[3H-indole3,2`-thiazolidine]-2,4`(1H)-dione (**4y**)

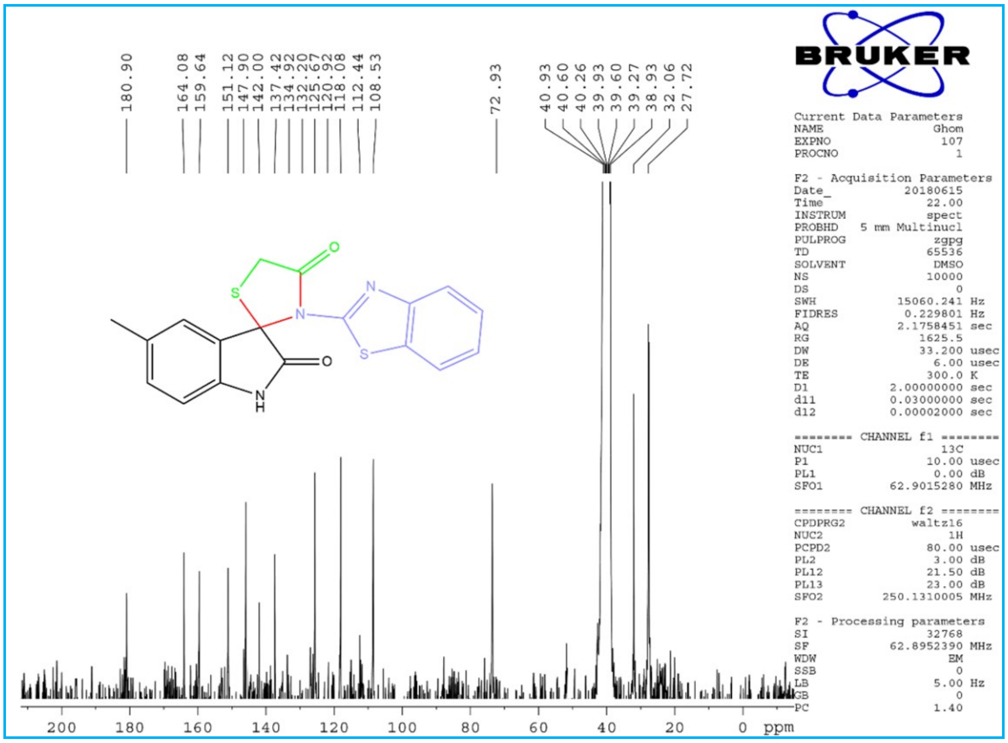

Figure S57: ^{13}C NMR spectrum of 5-Methyl-3`-(1,3-benzothiazol-2-yl)-spiro-[3H-indole3,2`-thiazolidine]-2,4`(1H)-dione (**4y**)

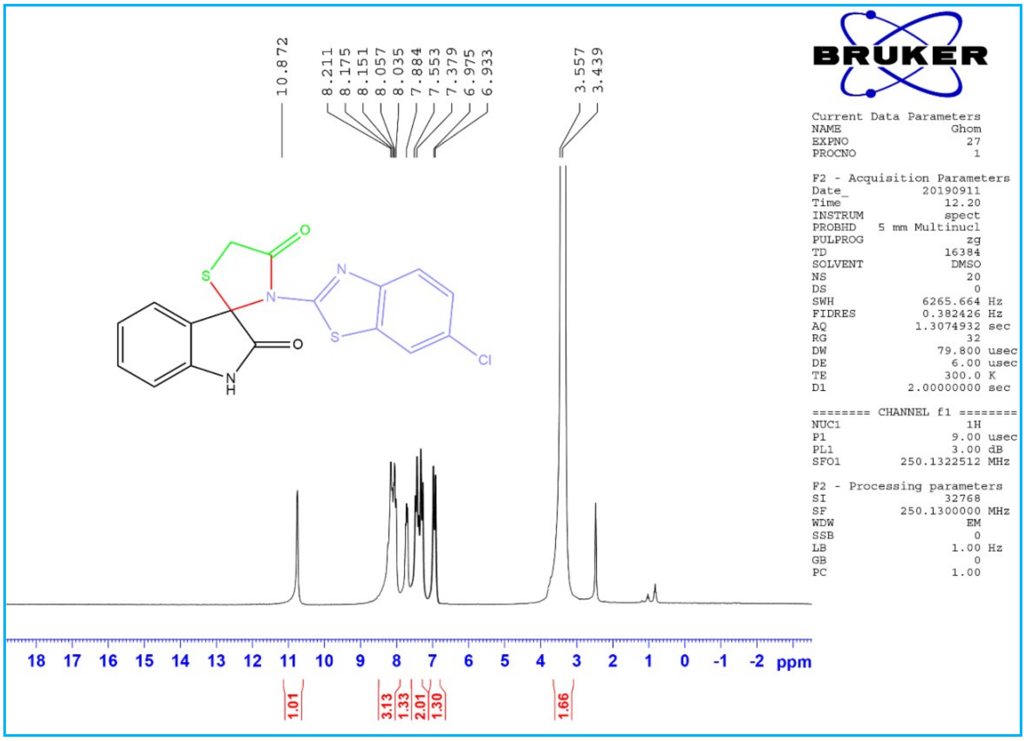

Figure S58: ¹H NMR spectrum of 3`-(6-chloro-1,3-benzothiazol-2-yl)-spiro-[3H-indole3,2`-thiazolidine]-2,4`(1H)-dione (**4z**)

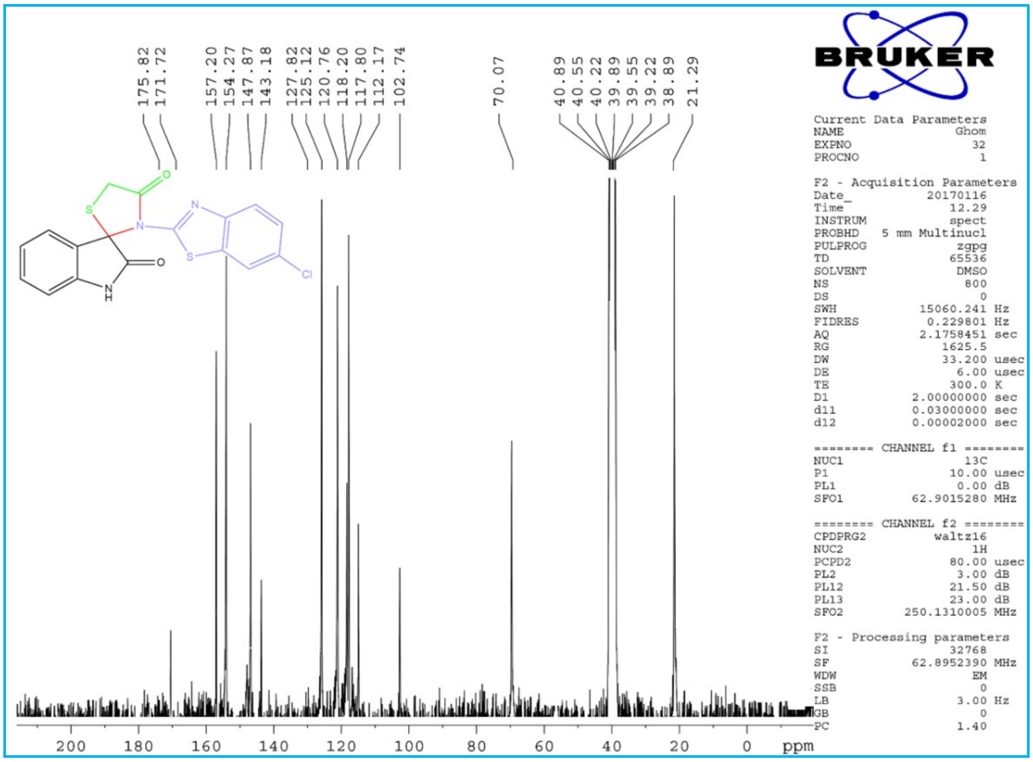

Figure S59: ¹³C NMR spectrum of 3`-(6-chloro-1,3-benzothiazol-2-yl)-spiro-[3H-indole3,2`-thiazolidine]-2,4`(1H)-dione (**4z**)
